# A functional screen identifies miRNAs that inhibit DNA repair and sensitize prostate cancer cells to ionizing radiation

**DOI:** 10.1093/nar/gkv273

**Published:** 2015-04-06

**Authors:** Koji Hatano, Binod Kumar, Yonggang Zhang, Jonathan B. Coulter, Mohammad Hedayati, Brian Mears, Xiaohua Ni, Tarana A. Kudrolli, Wasim H. Chowdhury, Ronald Rodriguez, Theodore L. DeWeese, Shawn E. Lupold

**Affiliations:** 1 The James Buchanan Brady Urological Institute and Department of Urology, The Johns Hopkins University School of Medicine, Baltimore, MD, USA; 2 The Department of Radiation Oncology and Molecular Radiation Sciences, The Johns Hopkins University School of Medicine, Baltimore, MD, USA; 3 Shanghai Institute of Planned Parenthood Research, National Population & Family Planning Key Laboratory of Contraceptive Drugs and Devices. 2140 Xietu Rd., Shanghai 200032, China; 4 The Department of Urology, University of Texas Health Science Center at San Antonio, San Antonio, TX, USA; 5 The Sidney Kimmel Comprehensive Cancer Center, The Johns Hopkins University School of Medicine, Baltimore, MD, USA

## Abstract

MicroRNAs (miRNAs) have been implicated in DNA repair pathways through transcriptional responses to DNA damaging agents or through predicted miRNA regulation of DNA repair genes. We hypothesized that additional DNA damage regulating miRNAs could be identified by screening a library of 810 miRNA mimetics for the ability to alter cellular sensitivity to ionizing radiation (IR). A prostate cancer *Metridia* luciferase cell model was applied to examine the effects of individual miRNAs on IR sensitivity. A large percentage of miRNA mimetics were found to increase cellular sensitivity to IR, while a smaller percentage were protective. Two of the most potent IR sensitizing miRNAs, miR-890 and miR-744–3p, significantly delayed IR induced DNA damage repair. Both miRNAs inhibited the expression of multiple components of DNA damage response and DNA repair. miR-890 directly targeted MAD2L2, as well as WEE1 and XPC, where miR-744–3p directly targeted RAD23B. Knock-down of individual miR-890 targets by siRNA was not sufficient to ablate miR-890 radiosensitization, signifying that miR-890 functions by regulating multiple DNA repair genes. Intratumoral delivery of miR-890 mimetics prior to IR therapy significantly enhanced IR therapeutic efficacy. These results reveal novel miRNA regulation of DNA repair and identify miR-890 as a potent IR sensitizing agent.

## INTRODUCTION

Ionizing radiation (IR) is a useful modality to treat multiple types of cancers. The primary cellular injury associated with IR is DNA damage; in particular DNA double strand breaks (DSBs) (1). Activated DNA damage response (DDR) pathways control downstream effectors which can determine cellular fates such as DNA repair, cell cycle arrest or apoptosis. Tumor cells often present radiation protective phenotypes which can lead to IR treatment failure. A number of mechanisms account for this resistance including tumor microenvironment and altered expression of DDR and DNA repair pathway components (1). Thus, a better understanding of DDR and DNA repair pathways in cancer may lead to improved treatment design and efficacy.

In previous studies we have applied siRNA library screening to determine the influence of DNA repair gene knock-down on prostate cancer sensitivity to IR (2). Specifically, individual DNA repair genes were temporarily inhibited by siRNA-mediated knock-down and cells were then treated with low dose IR. Relative changes in cell viability were then used to rank the IR-sensitizing potency of each gene. The most potent target genes identified in this screen were DNA-PK, MAD2L2 and BRCA2, all of which are components of DNA DSB repair. A component of nucleotide excision repair, RAD23B, was also identified as a radiosensitizing target. These studies demonstrated the utility of high-throughput radiation sensitivity screens to identify important genes in DNA repair and radiation response. Here we have applied a similar approach to screen and rank over 800 microRNAs (miRNAs) for IR sensitization potency, anticipating that the most potent IR sensitizing miRNAs may also regulate DNA repair. A novel cell viability reporter assay, based on constitutively expressed and secreted *Metridia* Luciferase (MLuc), was applied for high sensitivity and low cost analysis (3).

miRNAs are small non-coding RNAs which suppress the translation of specific mRNAs by the targeted recruitment and binding of the miRNA-induced silencing complex (miRISC) (4). Target gene specificity is determined through complementary binding of a short miRNA ‘seed sequence’, consisting of only 6–8 nucleotides, to the 3′ Untranslated Region (UTR) of target mRNAs (5). From this it is calculable that a single miRNA can regulate hundreds of different of genes (6). Gene expression and functional studies indicate that miRNAs regulate a variety of normal developmental and physiological processes, as well as pathways involved in human diseases such as cancer (7). For example, individual miRNAs have been shown to possess tumor suppressive and oncogenic properties (8). Growing evidence also supports that miRNA genes are responsive to DNA damage and that they can regulate DDR and DNA repair pathways (9,10). However, the impact of miRNAs on DNA repair and therapeutic resistance has not yet been fully characterized.

In summary, we report the results of a high-throughput screen to analyze the effects of individual miRNA mimetics on cell viability and sensitivity to IR using a prostate cancer MLuc model. A number of IR sensitizing miRNAs were identified and verified in several additional cellular models. Top ranking IR sensitizing miRNAs were found to regulate multiple genes involved in DDR and DNA repair pathways. Moreover, treatment with these miRNA mimetics significantly delayed the repair of IR-induced DNA damage. A single intratumoral administration of the most potent IR-sensitizing miRNA mimetic, miR-890, significantly enhanced tumor response to IR therapy. These results shed new light on the influence of miRNAs on DNA repair and support the potential use of miRNA mimetics as radiation sensitizing agents.

## MATERIALS AND METHODS

### Reagents and antibodies

Anti-human MAD2L2 (612266) and RAD23B (611018) were purchased from BD Biosciences (San Jose, CA, USA). Anti-human WEE1 (#4936), XPC (#12701), KU80 (#2180), XLF (#2854) and MCL1 (#5453) were purchased from Cell Signaling Technology (Danvers, MA, USA). Anti-human ACTB (AC-15) was purchased from Sigma-Aldrich (St. Louis, MO, USA). Anti-human phospho-histone H2AX (05–636) was purchased from EMD Millipore (Billerica, MA, USA). miRNA library and control miRNA (miR-cel-239b) were purchased from Thermo Scientific (Waltham, MA, USA). DNA-PK, MAD2L2 and WEE1 siRNA was purchased from Qiagen (Germantown, MD, USA) as previously described (2) and shown in Supplementary Table S1.

### Cell culture

The human prostate cancer cell lines LNCaP, C4–2, PC3 and DU145 were purchased from ATCC. LNCaP-MLuc and PC3-MLuc cells (stably transfected with the pDonor-hβ-Actin-hMLuc vector) were previously developed (3) and were maintained in RPMI 1640 containing 10% FBS and 5 μg/ml Blasticidin at 37°C and 5% CO_2_. Parental cell lines were authenticated by the DNA Diagnostics Center (Fairfield, OH) and confirmed mycoplasma free (November 2014).

### High-throughput functional screening using miRNA library

MLuc cell viability assays were completed as previously described (3). Two days before IR, 2×10^3^ LNCaP-MLuc cells were separately transfected with 20 nM of 810 different miRNA mimetics using Lipofectamine 2000 (Invitrogen, Grand Island, NY) in individual wells of 96 well plates. Each miRNA was transfected in quadruplicate and control miRNA and DNA-PK siRNA were each included in three wells of each 96 well plate. Half of the plates were irradiated 48 h after transfection (4 Gy in a Gammacell 40 [Nordion] 137Cs radiator at approximately 0.5 Gy/min) or untreated, while the other half remained untreated. The IR dose (4 Gy) was selected in assay-development studies (2–8 Gy) for optimum radiation-sensitization by DNA-PK siRNA. Seven days after IR, 50 μl of media was removed and replaced with 50 μl of fresh media. Eleven days after IR, MLuc activity was examined to quantify viable cell density, normalizing to the control miRNA.

### MLuc viability assay

Two days before IR, LNCaP-MLuc and PC3-MLuc cells were transfected with miRNA mimetics and/or siRNAs at indicated doses using Lipofectamine 2000 or RNAiMax (Invitrogen). On day 0, the cells were irradiated (4 Gy). On day 11, the cell culture media was then assayed for MLuc activity to quantify the relative cell density.

### Clonogenic assay

DU145 and PC3 cells were transfected with control, candidate miRNAs or siRNAs and grown for 48 h, after which cell dilutions were plated and irradiated immediately at different doses. The cells were grown for 14 days, fixed and stained with crystal violet; colonies with greater than 30 cells were scored, and surviving fraction was calculated as previously described (2). Survival curves were fitted by GraphPad Prism software by nonlinear regression analysis and a user defined equation for cell survival analysis due to radiation injury.

### γ-H2AX foci formation

DU145 and PC3 cells were transfected with 20 nM of control or candidate miRNAs and were seeded on glass slides. The cells were incubated for 48 h and then irradiated (4 Gy) or untreated. The γ-H2AX foci formation was evaluated at 1, 4, 8, 12 and 24 h after IR. The cells were fixed with 4% formaldehyde for 15 min, followed by treatment with 0.2% Triton X-100 for 10 min. The cells were blocked with 1% BSA for 1 h and then incubated with γ-H2AX antibody (1:1000) for 30 min. The cells were labeled with Alexa Fluor antibody (Invitrogen) for 30 min and counterstained with Prolong Gold with DAPI (Invitrogen). Foci were visualized with a TE2000-E immunofluorescent microscope and analyzed by NIS-Elements AR software (Nikon, Tokyo, Japan). Images were photographed at the same exposure time under a ×20 objective. Over 300 cells were counted from more than three random fields under a ×20 objective for each experiment, and the percentage of cells containing >10 fluorescent foci was calculated as previously reported (11).

### Comet assay

DU145 and PC3 cells were transfected with 20 nM of control or radiation sensitizing miRNAs and then treated with 4 Gy of IR 48 h post transfection. At 4 h after IR, the CometAssay® from Trevigen (Gaithersburg, MD, USA) was performed according to manufacturer instructions. Briefly, cells were mixed with low melting point agarose and plated on microscope slides and allowed to gel. Cells were then lysed under neutral buffer followed by rinse in TBE buffer (10.8% [w/v] tris base, 5.5% [w/v] boric acid, 0.93% [w/v] EDTA). After electrophoresis in TBE buffer for 40 min at 1 V/cm, slides were washed in water and dehydrated with ethanol, air dried overnight and then treated with SYBR Green for DNA staining. Comets were imaged by Imager.Z1 fluorescent microscopy (Carl Zeiss AG, Oberkochen, Germany) and analyzed using CometScore. The tail moment was calculated as the average of at least 50 comets.

### 3′UTR dual luciferase enzyme assay

3′UTR target regions were amplified from 293T genomic DNA (human embryonic kidney cells) by polymerase chain reaction (PCR). Primer sequences were designed to incorporate *Spe*I and *Hind*III restriction enzyme sites (Supplementary Table S2). PCR products were subcloned into pMIR-REPORT luciferase expression reporter vector (Ambion, Grand Island, NY) to generate pMIR-3′UTR reporter. Point mutations were generated by QuikChange II XL Site-Directed Mutagenesis Kit (Agilent Technologies, Columbia, MD, USA) according to the manufacturer's instructions (Supplementary Table S2). LNCaP or 293T cells were transfected with 20 nM of control or miRNA mimetic, 80 ng of pMIR-3′UTR reporter vector and 20 ng of reference pRL-CMV *Renilla* reporter vector. After 48 h, the reporter activity was assessed using a dual luciferase enzyme assay as previously described (12). Firefly luciferase signal was normalized against the internal control *Renilla* luciferase.

### Western blot analysis

Cells were transfected with miRNA mimetics or siRNAs at indicated doses and lysed in RIPA lysis buffer 48 h later. Protein samples were separated by sodium dodecyl sulfate polyacrylamide gel electrophoresis and transferred onto polyvinylidene fluoride membranes. Membranes were blocked with odyssey blocking buffer and incubated overnight at 4°C with anti-MAD2L2 (1:1000), anti-RAD23B (1:500), anti-WEE1 (1:1000), anti-XPC (1:1000), anti-KU80 (1:1000), anti-XLF (1:1000), anti-MCL1 (1:1000) or anti-ACTB (1:10 000). After washing, proteins were detected by IRDye secondary antibody (LI-COR, Lincoln, NE, USA) at room temperature for approximately 1 h. Images were analyzed with the Odyssey infrared imaging system (LI-COR). Signals for each protein were normalized to ACTB.

### *In vivo* tumor models

Animal studies were performed according to the protocols approved by the Animal Care and Use Committee at Johns Hopkins University. Athymic nude mice (nu/nu) aged 6 weeks (Harlan Laboratories Inc, Indianapolis, IN, USA) were inoculated with 5 × 10^6^ DU145 cells subcutaneously in the left thigh. Treatments started when tumors reached 0.1–0.3 cm^3^ in diameter. Tumors were randomized into five groups. Two animals were eliminated from the study due to illness and failure of progressive tumor growth. Neutral lipid emulsions of control miRNA mimetic, or miR-890 mimetic was prepared using the MaxSuppressor™ In Vivo RNA-LANCEr II kit (Bioo Scientific, Austin, TX, USA) according to the manufacturer's instructions. Mice were anesthetized with isoflurane and tumors were directly injected with 50 μl of reagents (PBS, 12.5 μg of liposomal control miRNA mimetic, or liposomal miR-890 mimetic). IR dose (6 Gy) was applied as previously reported (2). Two days after injection (day 0), radiation groups received 6 Gy local IR (7.14 Gy/min) using a J.L. Shepherd Mark 137Cs irradiator with the body shielded from the source. Tumors were measured every 2 days and volume (cm^3^) was calculated by length × width × height × 0.52. Tumor response was determined as reaching four times its original volume as previously reported (2).

### Statistical analyses

The results are reported as the mean ± standard error (SE) or standard deviation (SD). The differences between groups were evaluated by two-tailed, unpaired Student's *t*-test. The IC_50_ value was calculated using GraphPad Prism software. Tumor volume was evaluated by two-way ANOVA. For the extension of tumor quadrupling experiments, events (animals whose tumor volume was not yet 4-fold the size at injection) were plotted on a Kaplan-Meier curve and analyzed by log-rank (Mantel-Cox) test as previously reported (2). *P* < 0.05 was considered statistically significant.

## RESULTS

### High-throughput screening identifies miRNAs which modulate prostate cancer growth, survival and radiosensitivity

**Figure 1.**
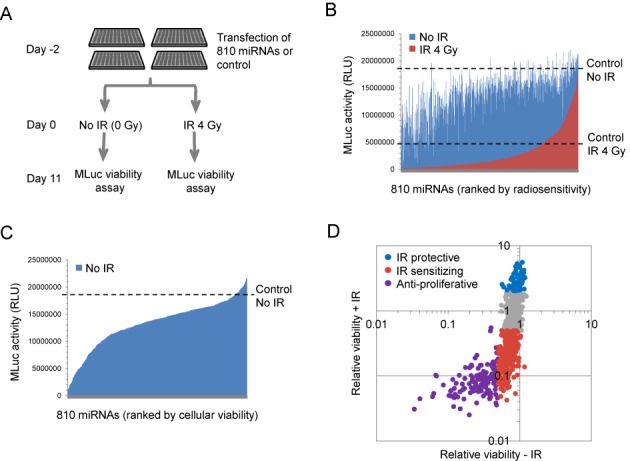
High-throughput functional screening for miRNAs which modulate prostate cancer viability and radiosensitivity. (**A**) A schematic representation of the high-throughput miRNA functional screening. (**B**) The average viable cell density (represented by MLuc activity) of irradiated (red) and non-irradiated (blue) LNCaP-MLuc cells on day 11. The data are organized as a bar graph waterfall plot for each miRNA and ranked by sensitivity to IR. Average cell response to control miRNAs, with and without IR, are represented by the two dashed lines. RLU; Relative Light Units. (**C**) Average LNCaP-MLuc viable cell density (represented by MLuc activity) after treatment with 810 different miRNAs, in the absence of IR. The results of the non-irradiated cells are represented again as a separate bar graph waterfall plot, with miRNAs ranked by viable cell density. Average signal with a control miRNA is represented as a dashed line. (**D**) Categories of miRNA responses based on cell viability and radiation sensitivity modulated by 810 miRNAs. The MLuc activity for each miRNA was normalized to control miRNAs and quantified as relative cell viability with or without IR.

A schematic representation of the experimental miRNA screen is shown in Figure 1A. A previously developed bioluminescent MLuc viability assay (3) was applied to measure prostate cancer cellular viability and sensitivity to IR following the transfection of 810 human miRNA mimetics. LNCaP-MLuc cells were separately transfected with individual miRNA mimetics, or control miRNAs, in replicates in multiwell plate format. After 48 h, one group of cells was irradiated (4 Gy), while the other group remained untreated. The secreted MLuc activity was then quantified on the eleventh day to measure relative cell viability and therapeutic effect. An siRNA targeting DNA-PK was utilized as a positive control for radiation sensitization (Supplementary Figure S1). Duplicate samples for each miRNA were also applied to analyze assay reproducibility (Supplementary Figure S2).

The resulting MLuc activity, representing viable cell density over eleven days, is shown in Figure 1B for both irradiated cells (red) and non-irradiated cells (blue). The data are organized as a bar graph waterfall plot for each miRNA, based on radiosensitivity, with the most radiation sensitizing miRNAs on the left. The results indicate that over half of the miRNA mimetics enhanced sensitivity to IR, when compared to a control miRNA, while a much smaller percentage of miRNAs was radiation protective (Figure 1B, red bars below ‘Control IR 4 Gy’, versus red bars above, respectively). Many miRNAs belonging to the same families produced matching IR sensitization phenotypes. For example, miRNAs from the miR-15/16 and miR-1/133 families were each observed to be IR sensitizing, whereas miRNAs from the miR-106b family were found to be radioprotective (Supplementary Figure S3). The relative radiosensitization results for all applied miRNA mimetics are available in Supplementary Table S3.

The experimental design also provided data on cellular responses to miRNA in the absence of irradiation (Figure 1C). It is notable that the majority of miRNAs reduced viable cell density, when compared to the control miRNA. These results are consistent with the common global miRNA down-regulation observed in human cancers (13). Cellular responses from this screen were separated into three categories: miRNAs which reduced growth or viability by over 50% in the absence of IR were considered anti-proliferative miRNAs; miRNAs which were not anti-proliferative, but enhanced radiation induced cell death by over 50%, were considered radiation sensitizing miRNAs; and miRNAs which increased cell survival by over 2-fold after irradiation were considered radioprotective (Figure 1D and Supplementary Table S3). Among 810 miRNAs, 127 (15.7%) were considered anti-proliferative, 75 (9.3%) were identified as radioprotective, and 324 (40.0%) were identified as radiosensitizing.

### Verification of candidate radiation sensitizing miRNAs using multiple cell lines

**Figure 2.**
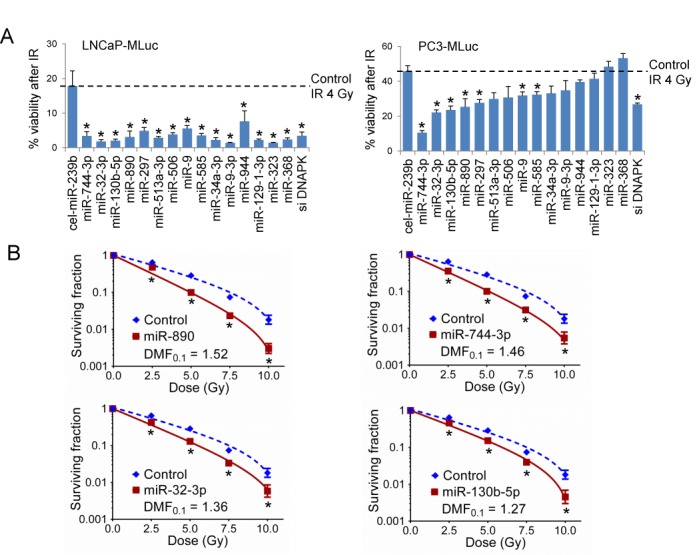
Candidate radiation sensitizing miRNA verification by MLuc assay and clonogenic survival assay. (**A**) LNCaP-MLuc (left) or PC3-MLuc (right) cell radiosensitivity following transfection with 20 nM of candidate miRNAs, negative control miRNA (cel-miR-239b) or positive control DNA-PK siRNA (siDNAPK). The% cell viability (mean ± SE, *n* = 3) following IR (4 Gy) represents the ratio of MLuc activity for irradiated cells to non-irradiated cells for each miRNA on day 11. Control miRNA is noted by the dash line. *, *P* < 0.05. The miRNAs were organized based on the PC3-MLuc cell radiosensitivity. (**B**) Clonogenic survival assay of DU145 cells transfected with 20 nM of candidate radiation sensitizing miRNAs or control miRNA. The surviving fraction is reported (mean ± SD, *n* = 3) following the indicated doses of IR. *, *P* < 0.05. DMF, Dose Modifying Factor.

The top 15 candidate radiation sensitizing miRNAs were selected for repeat assays using two prostate cancer cell lines which stably expressed the MLuc cell viability reporter, LNCaP-MLuc and PC3-MLuc (3) (Figure 2A). All 15 miRNA candidates were verified as IR sensitizing in LNCaP-MLuc cells, and 7 miRNAs were confirmed to also sensitize PC3-MLuc cells. Four of these miRNAs (miR-890, miR-744–3p, miR-32–3p and miR-130b-5p) demonstrated equal or greater radiosensitization efficiency when compared to the DNA-PK siRNA positive control, in both cells (Figure 2A). The IR sensitizing potential of these miRNAs was further quantified by clonogenic survival assays in DU145 prostate cancer cells. The dose-modifying factor (DMF_0.1_) for each miRNA was calculated as the ratio of IR dose required to cause 90% cell death by control miRNA versus the dose required to cause 90% cell death by the candidate miRNAs. miR-890 was identified as the most potent IR sensitizer, with a DMF_0.1_ of 1.52 (Figure 2B). miR-744–3p was similarly potent with a DMF_0.1_ of 1.46. Both miRNA mimetics were confirmed as IR sensitizers in PC3 cells by clonogenic survival assays (Supplementary Figure S4). We next anticipated that these miRNAs may function through targeted regulation of DNA repair processes.

### miR-890 and miR-744–3p radiosensitize cancer cells through inhibition of DNA repair

**Figure 3.**
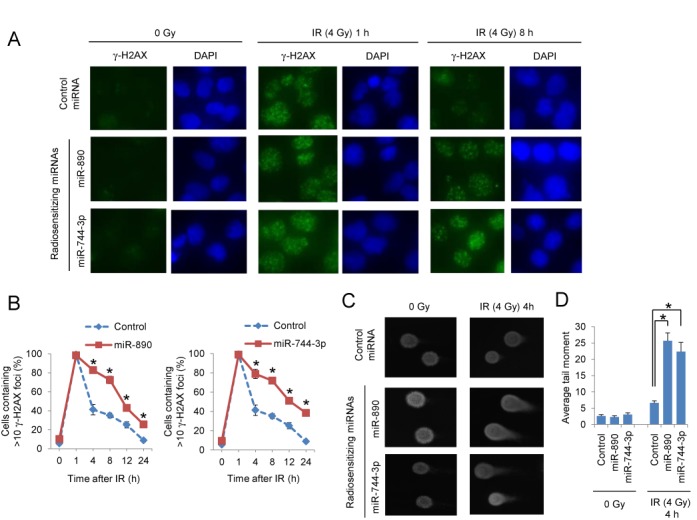
DSB repair delay by radiation sensitizing miRNAs. (**A**) Immunofluorescent staining of γ-H2AX foci (green) in DU145 cells transfected with control, miR-890 or miR-744–3p miRNAs in untreated (0 Gy) or 1 and 8 h after IR (4 Gy) treatment. Nuclei were stained with DAPI (blue). (**B**) Quantification of γ-H2AX foci. The percentage of cells containing >10 γ-H2AX foci (mean ± SE, *n* = 3) is reported for each time point and treatment group. *, *P* < 0.05. (**C**) Comet assay of DU145 cells transfected with control, miR-890 or miR-744–3p miRNAs which were either untreated (0 Gy) or 4 h after IR (4 Gy) treatment. (**D**) Quantification of the average tail moment (mean ± SE, *n* = 50) is reported for each miRNA and treatment condition. *, *P* < 0.05.

DNA repair pathways play an important role in the cellular response to IR. A hallmark of DNA DSB detection and repair is phosphorylation of H2AX. Following DNA repair, these γ-H2AX foci are resolved by phosphatase activity (14). The influence of miR-890 and miR-744–3p on the formation and resolution of γ-H2AX foci was therefore examined over 24 h using irradiated DU145 and PC3 cells. Cells were transfected with control or IR sensitizing miRNAs and irradiated (4 Gy) 48 h later. Within 1 h of IR, γ-H2AX foci formation was visible in all treatment groups (Figure 3A and Supplementary Figure S5A). By 8 h, the control treated cells had resolved most of the γ-H2AX foci. However, the radiation sensitizing miRNAs, miR-890 and miR-744–3p, significantly delayed γ-H2AX resolution over the 24 h time period (Figure 3A, B and Supplementary Figure S5A, B). To more directly study DNA damage and repair, miRNA transfected DU145 and PC3 cells were irradiated and examined by the single cell gel electrophoresis comet assay. Four hours following IR treatment, DNA comet tails moments were significantly extended in cells transfected with miR-890 and miR-744–3p, when compared to cells transfected with control miRNA (Figure 3C, D and Supplementary Figure S5C, D). Collectively these data support that miR-890 and miR-744–3p inhibited DNA repair processes, which likely contributed to the observed IR sensitization phenotype.

We have further examined the impact of radiation sensitizing miRNAs on IR-induced cell cycle checkpoint control using DU145 and PC3 cells (Supplementary Figure S6). In all samples, IR (4 Gy) induced the expected G2 cell cycle arrest, which peaked at 16 h after treatment. There were no significant differences in cell cycle in miR-890 or miR-744–3p transfected cells, relative to controls, suggesting that altered cell cycle regulation was not responsible for the observed differences in DNA repair.

### miR-890 and miR-744–3p regulate multiple components of DDR and DNA repair

**Figure 4.**
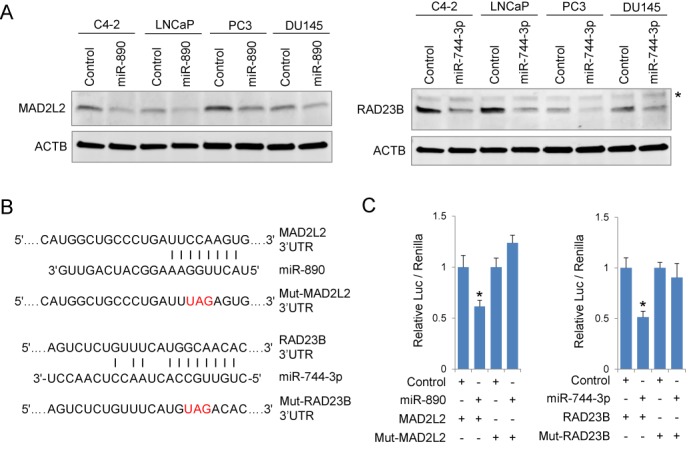
miR-890 and miR-744–3p directly target MAD2L2 and RAD23B. (**A**) Western blot assays of four prostate cancer cell lines, C4–2, LNCaP, PC3 and DU145, for MAD2L2 (left) and RAD23B (right) 48 h after 20 nM of miRNA mimetic transfection. Asterisk represents a nonspecific band. (**B**) Schematic of MAD2L2 and RAD23B 3′UTR miRNA binding sites. Mutated (Mut) MAD2L2 and RAD23B seed sequences are indicated (red). (**C**) Luciferase activities following transfection with indicated 3′UTR reporters and miRNA mimetics in LNCaP cells. Relative luciferase activity (mean ± SE, *n* = 4) is normalized to control miRNA for each reporter. *, *P* < 0.05 relative to control miRNAs.

We have previously characterized several DNA repair genes as potent IR sensitizing targets including DNA-PK, MAD2L2, BRCA2, NBN, RAD23B and RAD54L (2). These genes were examined to determine whether they may be direct targets of the identified IR sensitizing miRNAs by *in silico* analysis (Supplementary Table S4). miR-890 and miR-744–3p were predicted to target two of these genes, MAD2L2 and RAD23B, respectively, with mirSVR Scores < −0.5, indicating a high probability of target gene down-regulation (15). To verify this prediction, miRNA mimetics and controls were transfected into multiple prostate cancer cell lines and target proteins were evaluated by western blotting. miR-890 transfection significantly reduced MAD2L2 protein levels (Figure 4A, left), and miR-744–3p transfection resulted in significantly reduced RAD23B protein levels (Figure 4A, right). The 3′UTR regions encompassing the predicted miRNA target binding sites were then subcloned from each gene and placed downstream of the firefly luciferase gene in 3′UTR reporter vectors (Figure 4B). MAD2L2 and RAD23B 3′UTR reporter activities were significantly down-regulated by miR-890 and miR-744–3p transfection, respectively (Figure 4C). Mutation of the corresponding miRNA seed binding sites ablated miRNA mediated suppression (Figure 4B and C), indicating direct miRNA regulation of these genes.

**Figure 5.**
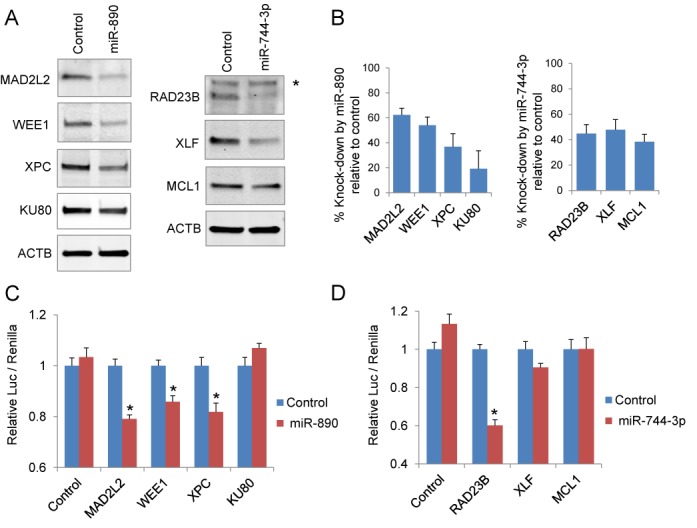
Multiple DDR and DNA repair pathway genes targeted by radiation sensitizing miRNAs. (**A**) Western blot analyses of MAD2L2, WEE1, XPC, KU80, RAD23B, XLF and MCL1 48 h after LNCaP transfection with 20 nM of miR-890 (left) and miR-744–3p (right). Asterisk represents a nonspecific band. ACTB was utilized as a loading reference. (**B**) Normalized percent protein knock-down (mean ± SE, *n* = 3) by miR-890 (left) and miR-744–3p (right), relative to control miRNA (from three separate experiments). (**C**) Luciferase activities following transfection with indicated 3′UTR reporters and miR-890 or control mimetics in 293T cells. (**D**) Luciferase activities following transfection with indicated 3′UTR reporters and miR-744–3p or control mimetics in 293T cells. Relative luciferase activity (mean ± SE, *n* =12) is normalized to control miRNA for each reporter. *, *P* < 0.05 relative to control miRNAs.

A single miRNA has the potential to regulate the expression of multiple genes (6). We therefore anticipated that miR-890 and miR-744–3p may regulate additional DDR pathway components. *In silico* analysis using microRNA.org predicted several additional DDR pathway targets for miR-890 and miR-744–3p, each with high ranking mirSVR scores (Supplementary Table S5). Western blot analyses confirmed that miR-890 transfection also reduced the predicted target genes WEE1, XPC and KU80 in LNCaP prostate cancer cells (Figure 5A and B). 3′UTR luciferase assays further support direct regulation of WEE1 and XPC, but not KU80, by miR-890 transfection (Figure 5C). miR-744–3p transfection was also found to reduce the levels of the predicted target proteins XLF and MCL1 (Figure 5A and B); however, these may not be through direct 3′UTR regulation (Figure 5D).

### Analysis of miR-890 and its multiple targets on cellular sensitivity to IR

**Figure 6.**
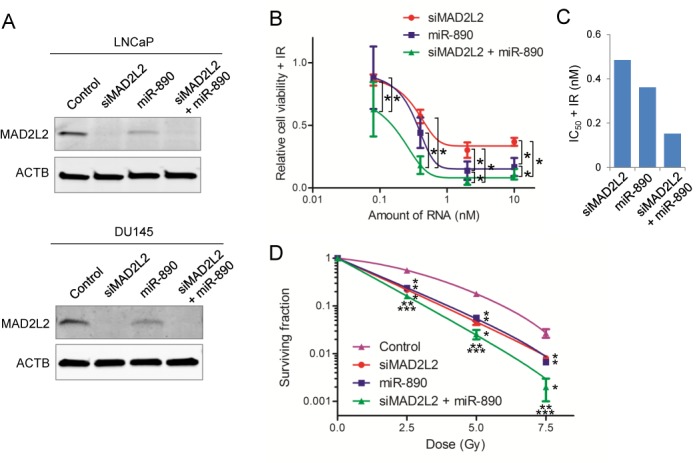
The effect of MAD2L2 siRNA and miR-890, alone or in combination, on IR therapy. (**A**) MAD2L2 knock-down by siRNA (siMAD2L2), miR-890, or combined siRNA and miR-890 in LNCaP and DU145 cells (10 nM). Western blot 48 h after transfection. ACTB was utilized as a loading reference. (**B**) IR sensitization potency of siMAD2L2 and/or miR-890 (0.08–10 nM) in LNCaP-MLuc cells. Relative cell viability (mean ± SE, *n* = 12) is presented as the MLuc activity after IR (4 Gy), as normalized by control miRNA. *, *P* < 0.05. (**C**) The calculated IC_50_ value of each treatment group, based on relative cell viability after IR in LNCaP-MLuc cells. (**D**) Clonogenic survival assay of DU145 cells transfected with control miRNA, siMAD2L2, miR-890, or combined siRNA and miR-890 (10 nM). The surviving fraction is reported (mean ± SD, *n* = 3) following the indicated doses of IR. *, *P* < 0.05 compared with control; **, *P* < 0.05 compared with siMAD2L2; ***, *P* < 0.05 compared with miR-890. DMF_0.1_ of siMAD2L2, miR-890 and combined siRNA and miR-890 is 1.58, 1.49 and 1.90, respectively.

The multifunctional nature of the identified miRNAs suggests that they may be more potent IR sensitizing agents than rationally designed siRNAs engineered to target a single DDR gene. To address this, cells were treated with IR sensitizing miRNAs, siRNAs, or combinations thereof. When LNCaP cells were transfected with an equal dose of MAD2L2-targeting siRNA or miR-890 (10 nM), the expression of MAD2L2 was most strongly down-regulated by the siRNA (Figure 6A). This effect was similarly seen in DU145 cells (Figure 6A). However, miR-890 transfection resulted in a similar or greater IR sensitizing efficacy, when compared to MAD2L2 siRNA (Figure 6B–D). Specifically, in LNCaP cells transfected with 0.08–10 nM of RNA, IR sensitization by miR-890 was observed to be moderately superior to MAD2L2 siRNA at higher transfected doses (Figure 6B and C). However, in DU145 cells transfected with high doses of miRNA or siRNA (10 nM), miR-890 and MAD2L2 siRNA displayed similar IR sensitizing efficiencies by clonogenic survival assays (Figure 6D). Similar studies were performed with siRNA knock-down of a second miR-890 target, WEE1, in LNCaP MLuc cells. The expression of WEE1 was also most strongly down-regulated by siRNA when compared to miR-890 (Supplementary Figure S7A). However, miR-890 and WEE1 siRNA showed similar radiation sensitizing efficacy over several doses (Supplementary Figure S7B and C). Collectively, these results support that miR-890 mimetics can function comparably to single-gene targeted siRNAs in cellular radiation sensitization, while not achieving the same potency of target gene knock-down.

These results may be explained by the ability of miR-890 to target multiple DDR genes. Interestingly, the combination of MAD2L2 siRNA and miR-890 resulted in greater LNCaP IR sensitization at all concentrations studied (Figure 6B and C), including at higher doses where MAD2L2 was maximally knocked down by siRNA (Figure 6A and Supplementary Figure S8). This observation was confirmed by clonogenic assay in DU145 cells transfected with higher doses (10 nM) of MAD2L2 siRNA and miR-890 (Figure 6A and D). Further, the combination of WEE1 siRNA and miR-890 also resulted in greater efficacy of IR therapy, when compared to WEE1 transfection alone (Supplementary Figure S7). Collectively, these data indicate that miR-890 functions by targeting multiple DNA repair genes, including MAD2L2 and WEE1.

### miR-890 mimetic sensitizes established prostate cancer tumors to IR

**Figure 7.**
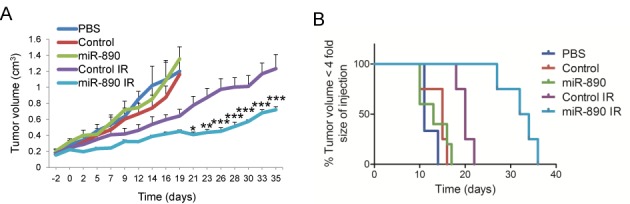
miR-890 mimetic injection enhances IR therapy of established prostate tumors. (**A**) Established subcutaneous DU145 tumors (*n* = 3–5 per group) were directly injected with PBS or miRNA mimetics in neutral lipid emulsions. Two days after injection (day 0) tumors were irradiated with 6 Gy or no IR. miR-890 significantly reduced tumor volume in irradiated groups when compared to controls. ***P < 0.001, **P < 0.01, *P < 0.05; two-way ANOVA. Mean ± SEM. (**B**) Extension of tumor quadrupling for DU145 tumor model. Events (animals whose tumor volume was not yet 4-fold the size at injection) were plotted by Kaplan-Meier curve. miR-890 significantly extended time to tumor quadrupling in irradiated groups when compared to controls. *P* < 0.01; log-rank (Mantel-Cox) test.

In view of the potent and multifunctional nature of miR-890 IR sensitization *in vitro*, we sought to determine whether a miR-890 mimetic could enhance the therapeutic effect of IR *in vivo*. Established subcutaneous DU145 prostate tumors were directly treated with miR-890 or control miRNA mimetics, formulated in neutral lipid emulsions, with a single intratumoral administration. Two days after injection, one cohort of tumors was irradiated with a single non-ablative dose of 6 Gy, while the other cohort was not irradiated. A final control cohort was treated with PBS only. Tumor volume was evaluated over 35 days and the time to tumor volume quadrupling was calculated. In the absence of irradiation, the tumors grew comparably with miR-890, control miRNA, or PBS treatment (Figure 7A and Supplementary Figure S9). In irradiated animals, the tumors that were pre-treated with miR-890 had significantly lower volumes when compared to miRNA control treated tumors. miR-890 pre-treatment significantly delayed the time to tumor quadrupling of irradiated mice by 14 days, when compared to the control miRNA (Figure 7B). These results support the use of miR-890 as a potent radiation sensitizing agent.

## DISCUSSION

DDR and DNA repair pathways influence cellular sensitivity to IR. Growing evidence supports that miRNAs are involved in DDR and DNA repair (9,10). Here we hypothesized that additional DNA repair modifying miRNAs could be identified through a high-throughput screen of miRNA mimetics for the ability to modify cellular survival following IR therapy. The results found that two potent IR sensitizing miRNAs, miR-890 and miR-744–3p, specifically target multiple components of DDR, DNA DSB repair and nucleotide excision repair. These miRNAs were among 324 candidate radiation sensitizing miRNAs and 75 candidate radiation protective miRNAs identified in the screen. Further investigation of these additional candidates is required to verify and characterize their roles in DDR and DNA repair. In lieu of these future studies, the existing data can be evaluated for previously reported DDR and repair modifying miRNAs. In the same way, the results observed in non-irradiated cells could be analyzed for miRNAs reported to regulate cell growth and cell survival pathways.

Multiple miRNAs have been reported to regulate DDR and DNA repair. A number of these miRNAs were found through transcriptional responses to IR treatment or through differential expression in IR-resistant cancer cells (10,16–23). For example, the miR-34 gene family is induced by IR through direct transcriptional activation by p53. Elevated miR-34 expression then results in decreased cellular proliferation and increased apoptosis (24–26). In our screen, miR-34c was found to be the most anti-proliferative miRNA of the 810 mimetics transfected in the absence of irradiation. Candidate DDR regulating miRNAs have also been found through *in silico* analyses of known DNA repair pathway genes. For example, miR-421 and miR-101 were identified as ATM and DNA-PK targeting miRNAs through 3′UTR analyses (27,28). Both of these reported IR-sensitizing miRNAs were found to be strong IR sensitizing miRNAs in our screen (Supplementary Table S3).

Several miRNAs have also been reported to protect cells from IR. Inhibition of these miRNAs may be an attractive treatment strategy to help overcome radiation resistance. Similarly, these miRNAs may serve as biomarkers to predict tumor susceptibility to IR. miR-21 is an oncogenic miRNA that is up-regulated in multiple cancers (29). Previous studies have reported miR-21 to be radiation protective through the regulation of PI3K/AKT signaling and autophagy (30). In another example, the cancer associated miR-106b family (29,31) has been reported to provide a radiation protective phenotype through the regulation of cell cycle progression (21). Both miR-21 and miR-106b were verified as radiation protective miRNAs in our screen. Several additional radiation modifying candidate miRNAs (9,10,16,18) were supported in our study, indicating the reliability of the screen and analysis. However, some contradictory results were also observed. For example, IR sensitivity was not potently influenced by miR-155 in our screens, despite previously reported observations (32). These results may reflect differences in radiation sensitivity or miRNA targeting between cancer cell types or anomalies within the library screen. These differences underscore the need to verify each candidate miRNA for its role in radiation response and DNA repair in specific cell types.

It is notable that the p53 status of cancer cells can influence radiation response due to its role in DDR and repair (33,34). Over half of human cancers have mutated or deleted p53, and p53 is often lost in more advanced disease (35). Here multiple cell lines of differential p53 gene status were applied. Specifically, LNCaP cells are p53 wild-type, where PC3 and DU145 cells are p53 null and mutant, respectively (34). While the functional screen was completed with WT p53 LNCaP cells, some of the candidate miRNAs showed common radiation sensitizing effect in p53 wild type and ablated cell types. We therefore anticipate that many of these miRNAs may be strong agents to use in combination with IR, regardless of the p53 status.

This study ultimately focused on two of the most potent radiation sensitizing miRNAs, miR-890 and miR-744–3p. Mechanistic studies demonstrated that these miRNAs delayed γ-H2AX resolution and DNA repair. Further experiments support that these miRNAs function by targeting multiple genes within DDR and DNA repair pathways. Specifically, miR-890 transfection reduced the protein expression of four DDR and DNA repair genes, MAD2L2, WEE1, XPC and KU80. Suppression of MAD2L2, a mitotic spindle assembly checkpoint protein, is known to cause hypersensitivity to IR and increased γ-H2AX foci formation (11,36). WEE1, a mitotic checkpoint protein and tyrosine kinase, is a component of DDR and inhibition of WEE1 has been reported to increase γ-H2AX foci formation and delay their resolution (37). In our study, miR-890 delayed γ-H2AX resolution and DNA repair without significant effects on cell cycle control, suggesting that miR-890-mediated IR sensitization may involve additional pathways beyond WEE1 cell cycle control (38). XPC is mutated in xeroderma pigmentosum and functions in nucleotide excision repair (39). XPC knock-down has also been reported to inhibit DSB repair and to increase cellular sensitivity to DNA damaging agents (40). KU80, encoded by XRCC5, is also a well characterized DDR and repair protein involved in non-homologous end joining and radiation sensitivity (41). Thus, miR-890 has the potential to enhance cellular sensitivity to DNA damaging agents through a variety of pathway targets. While it has been hypothesized that individual miRNAs may target multiple genes within the same pathway to produce a given phenotype (42), this concept has not been significantly challenged. To investigate the multifunctional nature of miR-890 to mediate radiation sensitization, we used siRNA to knock-down the miR-890 targets, MAD2L2 and WEE1, in order to determine if miR-890 retained IR sensitizing activity in the absence of these primary targets. The results show that miR-890 enhances IR sensitivity even after significant target gene knockdown, supporting the concept that miRNAs can function by targeting multiple genes in related pathways.

The primary transcript for miR-890 is not well characterized, but it is presumed to include miR-888, miR-892a and miR-892b due to their proximity. The additional miRNA mimetics from this cluster were also found to be radiation sensitizing in our screen (Supplementary Table S3), indicating that this gene region may play a specialized role in DNA repair. miR-890 expression is low or absent in most human tissues, including the prostate (43). Interestingly, miRNAs from this gene region are uniquely expressed at high levels in human epididymis which regulates sperm maturation by the secretion of proteins, also suggesting a potential role for these miRNAs in fertility (43,44). The incidence of epididymal tumors is very rare and represents at most 0.03% of all male cancers, in sharp contrast to almost 20% for prostate cancer in Western countries (45). Perhaps the miR-890 gene cluster contributes to this differential cancer susceptibility due to increased cellular sensitivity to DNA damage. Further studies will be required to delineate the natural role of miR-890 in cellular biology, DNA repair, reproductive biology and cancer. These studies may be complicated by the absence of miR-890 in more primitive mammals.

The results presented here support the potential use of miR-890 as a radiation sensitizing agent. miRNA mimetics and inhibitors have shown promise as therapeutic agents in pre-clinical models and a few are being translated for human clinical trials. For example a miR-122 inhibitor, Miravirsen, is currently in clinical trials for the treatment of Hepatitis C (46). Further, a liposome-based miR-34 mimetic (MRX34) entered Phase I clinical trials in patients with advanced hepatocellular carcinoma in 2013 (47). Here we have demonstrated that pre-treatment of established tumors with a single dose of miR-890 mimetic significantly enhanced the therapeutic effect of IR therapy. A non-ablative dose of 6 Gy was applied to allow for proof-of-concept detection of differential tumor response in these studies. IR is a commonly applied treatment for multiple cancer types, including prostate cancer. It can be administered through targeted external beam radiation therapy, localized radioactive seed placement, or systemically targeted radiotherapeutics. Thus the selective delivery of miR-890 to prostate tumors, or activation of endogenous miR-890 in prostate cancer cells, prior to therapy may significantly enhance the therapeutic index of IR or other DNA damaging therapies by preventing DNA repair.

In summary, a series of high-throughput functional screens have identified several miRNAs capable of regulating cancer cell radiation sensitivity and DNA repair. miR-890 sensitizes cancer cells to IR through multiple gene targets, including MAD2L2, supporting the concept that a single miRNA can simultaneously regulate multiple genes within a single pathway. These results suggest that miRNAs may have therapeutic potential in the treatment of cancers with IR.

## SUPPLEMENTARY DATA

Supplementary Data are available at NAR Online.

SUPPLEMENTARY DATA
