# Supplementary material for: A functional screen identifies miRNAs that inhibit DNA repair and sensitize prostate cancer cells to ionizing radiation
Source: Nucleic Acids Res. 2015 Apr 6;43(8):4075–86. doi: 10.1093/nar/gkv273 (PMC4417178; doi:10.1093/nar/gkv273)
Supplement: SUPPLEMENTARY DATA [file supp_43_8_4075__index.html]

A functional screen identifies miRNAs that inhibit DNA repair and sensitize prostate cancer cells to ionizing radiation — A functional screen identifies miRNAs that inhibit DNA repair and sensitize prostate cancer cells to ionizing radiation — SUPPLEMENTARY DATA 

# A functional screen identifies miRNAs that inhibit DNA repair and sensitize prostate cancer cells to ionizing radiation

## SUPPLEMENTARY DATA

**Files in this Data Supplement:**

- SUPPLEMENTARY DATA
