# Supplementary material for: A functional screen identifies miRNAs that inhibit DNA repair and sensitize prostate cancer cells to ionizing radiation
Source: Nucleic Acids Res. 2015 Apr 6;43(8):4075–86. doi: 10.1093/nar/gkv273 (PMC4417178; doi:10.1093/nar/gkv273)
Supplement: SUPPLEMENTARY DATA [file supp_gkv273_nar-03594-d-2014-File009.pdf]

Supporting Information

Methods

Cell cycle analysis

Prostate cancer cells were transfected with 20 nM of control or radiation sensitizing miRNAs and then treated with 4 Gy of IR 48 h post transfection. Cells were harvested 6, 16 or 30 h after IR and suspended in PBS. Ice-cold methanol:acetone (1:1) was added dropwise with intermittent vortexing and cells were stored overnight at −20 °C. Cells were washed with cold PBS and incubated with PI/RNase Staining Buffer (BD Pharmingen, Franklin Lakes, NJ) for 15 min. DNA content was measured using a FACSCalibur flow cytometer and analyzed using Cell Quest software (BD Biosciences, Franklin Lakes, NJ). Doublet discrimination was accomplished by gating on forward scatter-H v. FL2A and FL2A v. FL2-H. At least 12,000 events were collected and analyzed in each sample.

Supplementary Figure 1

The radiation sensitization efficacy of DNAPK siRNA in LNCaP-MLuc cells.

DNAPK siRNA was used as a positive control for each 96 well LNCaP-MLuc plate of the high throughput miRNA radiosensitivity screening. Two days before IR, each plate contained wells

which were transfected with either DNAPK siRNA or control. On day 0, plates were irradiated at 4 Gy or remained untreated. On day 11, the cell viability and radiation sensitivity were determined by the MLuc viability assay (RLU; Relative Light Units). (A) The MLuc activity of each group is presented (mean ± S.E., n=132). (B) Normalized Relative cell viability after IR (mean ± S.E., n=132) is presented as the relative MLuc activity of irradiated cells normalized by that of non-irradiated cells. *, $P < 0.05$.

Supplementary Figure 2

The reproducibility of the high-throughput functional miRNA screening.

The high-throughput miRNA screening for cell growth and IR response was performed with duplicate wells for each miRNA and treatment condition, forming Group 1 and Group 2. The correlation of viable cell number for each miRNA, as measured by relative MLuc activity normalized by control in Group 1 and Group 2, is plotted for the (A) non-irradiated and (B) IR samples. The linear correlation coefficient (R^2) between the two groups is noted.

Supplementary Figure 3

Radiosensitization by miRNAs from individual miRNA families.

The radiation sensitization of LNCaP-MLuc cells from the high-throughput miRNA screen for

the individual miRNA families miR-15/16, miR-1/133, and miR-106b are highlighted. The % cell viability (mean ± S.E., n≧4) following IR (4Gy) is presented by the ratio of MLuc activity for irradiated cells relative to non-irradiated cells for each miRNA on day 11. Control miRNA is noted by the dash line.

Supplementary Figure 4

miRNA mediated radiation sensitization in PC3 cells.

PC3 Clonogenic Assay. PC3 cells were transfected with 20 nM of miR-890, miR-744-3p or control miRNA (cel-miR-239b) and grown for 2 days, after which cells were irradiated at the indicated doses. The cells were grown for 14 days and colonies with greater than 30 cells were scored, and surviving fraction was calculated (mean ± S.D., n=3). *, $P < 0.05$ relative to control. DMF; Dose Modifying Factor.

Supplementary Figure 5

DSB repair delay by radiation sensitizing miRNAs in PC3 cells.

(A) Immunofluorescent staining of γ-H2AX foci in PC3 cells transfected with control, miR-890 or miR-744-3p miRNAs in untreated (0 Gy) or 1 and 8 h after IR (4 Gy) treatment. Nuclei were stained with DAPI. (B) Quantification of γ-H2AX foci. The percentage of cells

containing >10 γ-H2AX foci (mean ± S.E., n≧3) is reported for each time point and treatment group. *, $P < 0.05$. (C) Comet assay of PC3 cells transfected with control, miR-890 or miR-744-3p miRNAs which were either untreated (0 Gy) or 4 h after IR (4 Gy) treatment. (D) Quantification of the average tail moment (mean ± S.E., n≧50) is reported for each miRNA and treatment condition. *, $P < 0.05$.

Supplementary Figure 6

IR-induced cell cycle checkpoint control.

(A) Cell cycle analysis in DU145 and PC3 cells transfected with control, miR-890 or miR-744-3p miRNAs in untreated (0 h) or 6, 16 and 30 h after IR (4 Gy) treatment. (B) The cell cycle distribution (G0/G1, S or G2/M) was analyzed for each treatment.

Supplementary Figure 7

The effect of WEE1 siRNA and miR-890, alone or in combination, on IR therapy.

(A) WEE1 and MAD2L2 knock-down by siRNA for WEE1 (siWEE1), miR-890, or combined siWEE1 and miR-890 in LNCaP cells (10 nM). Western blot of WEE1 and MAD2L2 48 h after transfection. ACTB was used as a control for protein loading (B) IR sensitization potency of siWEE1 and/or miR-890 (0.08-10 nM) in LNCaP-MLuc cells. Relative cell

viability (mean ± S.E., n=6) is presented as the MLuc activity after IR (4Gy), as normalized by control miRNA. (C) The calculated IC_{50} value of each treatment group, based on relative cell viability after IR.

Supplementary Figure 8

The knock-down efficacy of target genes by miR-890 and MAD2L2 siRNA.

LNCaP cells were transfected with 20 nM of control, miR-890 mimetics or serial dilutions of MAD2L2 siRNA and were incubated for 48 h. Western blot analyses were performed to detect MAD2L2 and WEE1. ACTB was used as a control for protein loading.

Supplementary Figure 9

Individual DU145 tumor growth curves.

(A-E), Subcutaneous DU145 tumors were directly injected with PBS, liposomal control miRNA mimetic, or liposomal miR-890 mimetic on day -2. On day 0, animals were divided into groups that received either 6 Gy IR or non-irradiated. Tumors were measured every other day and individual tumor growth curves are reported. (A) PBS (n=3); (B) Control miRNA (n=4); (C) miR-890 (n=5); (D) Control miRNA + IR (n=4); (E) miR-890 + IR (n=4).

Supplementary Table 1

The sequence of siRNAs used in this study.

Supplementary Table 2

The sequence of primers used in this study.

Supplementary Table 3

Summary of the results of the high-throughput miRNA screening for cell growth and IR response.

810 miRNA mimetics were transfected into LNCaP-MLuc cells. After two days, cells were irradiated or non-irradiated, and the relative number of surviving cells was quantified by MLuc assay on day 11 post IR. The MLuc activity, representing relative cell viability, for cells transfected with each miRNA was normalized to those transfected with a control miRNA. Results are categorized as cell growth, for non-irradiated samples, and radiation sensitization, for irradiated samples. Anti-proliferative miRNAs were defined as those which inhibited cell growth by over 50% without IR. IR sensitizing or IR protective miRNAs were defined as those which increased IR induced-cell death by over 50% or increased cell survival by over 2

fold, respectively, excluding anti-proliferative miRNAs.

Supplementary Table 4

Radiation sensitizing miRNAs predicted to target radiation sensitizing DDR pathway genes using *in silico* analysis, microRNA.org. The highlighted cells corresponding to the miRNAs with a mirSVR Score < -0.5.

Supplementary Table 5

Potential DDR pathway genes predicted to be targeted by miR-890 or miR-744-3p using *in silico* analysis, microRNA.org, and corresponding mirSVR scores.

Supplementary Figure 1

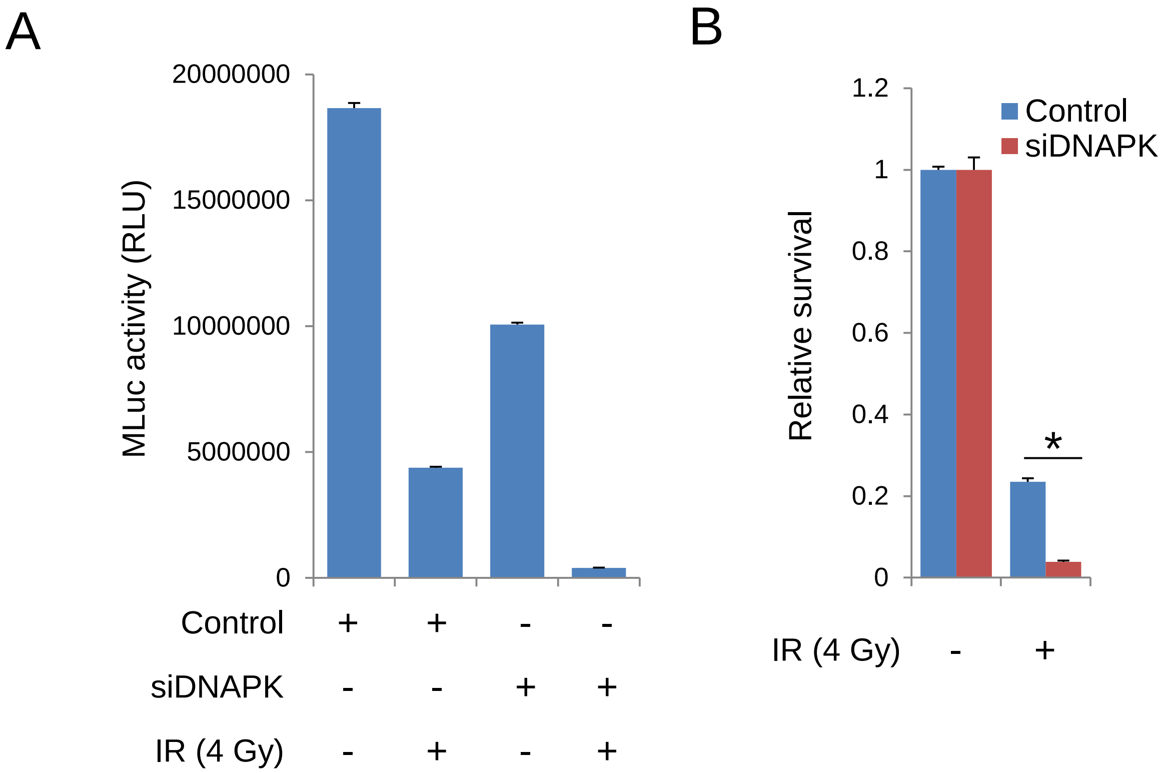

Supplementary Figure 2

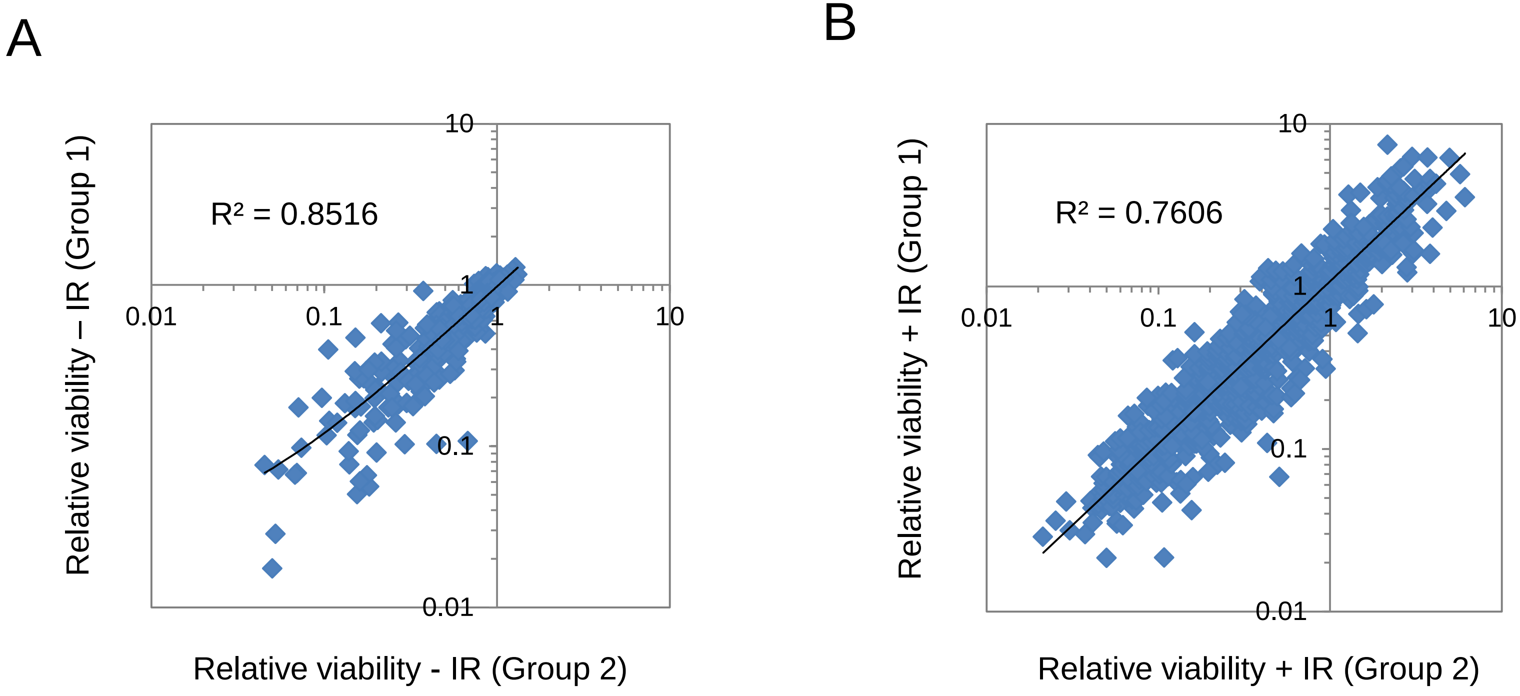

Supplementary Figure 3

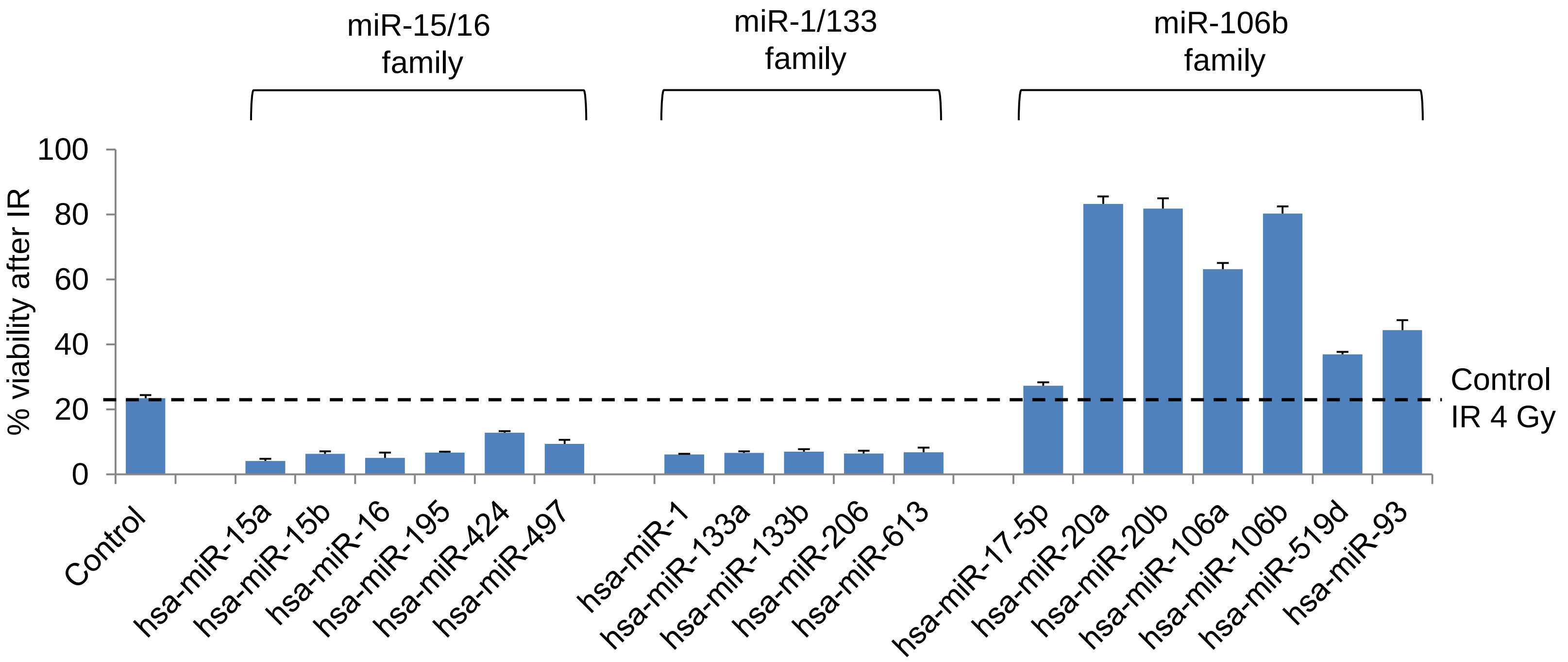

Supplementary Figure 4

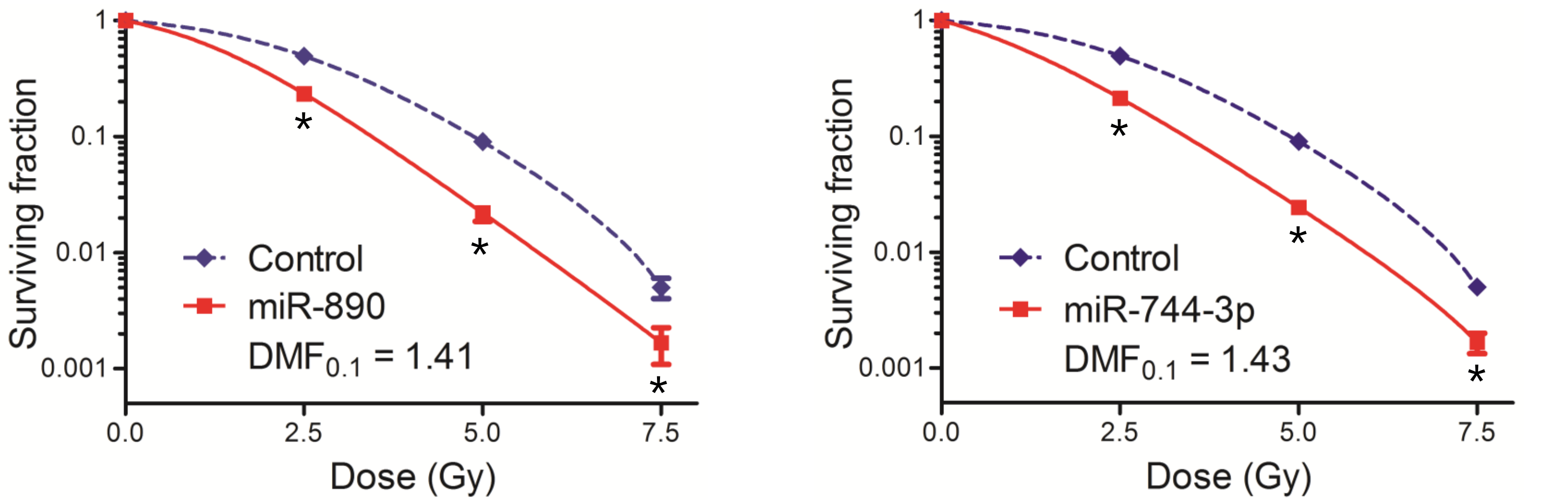

Supplementary Figure 5

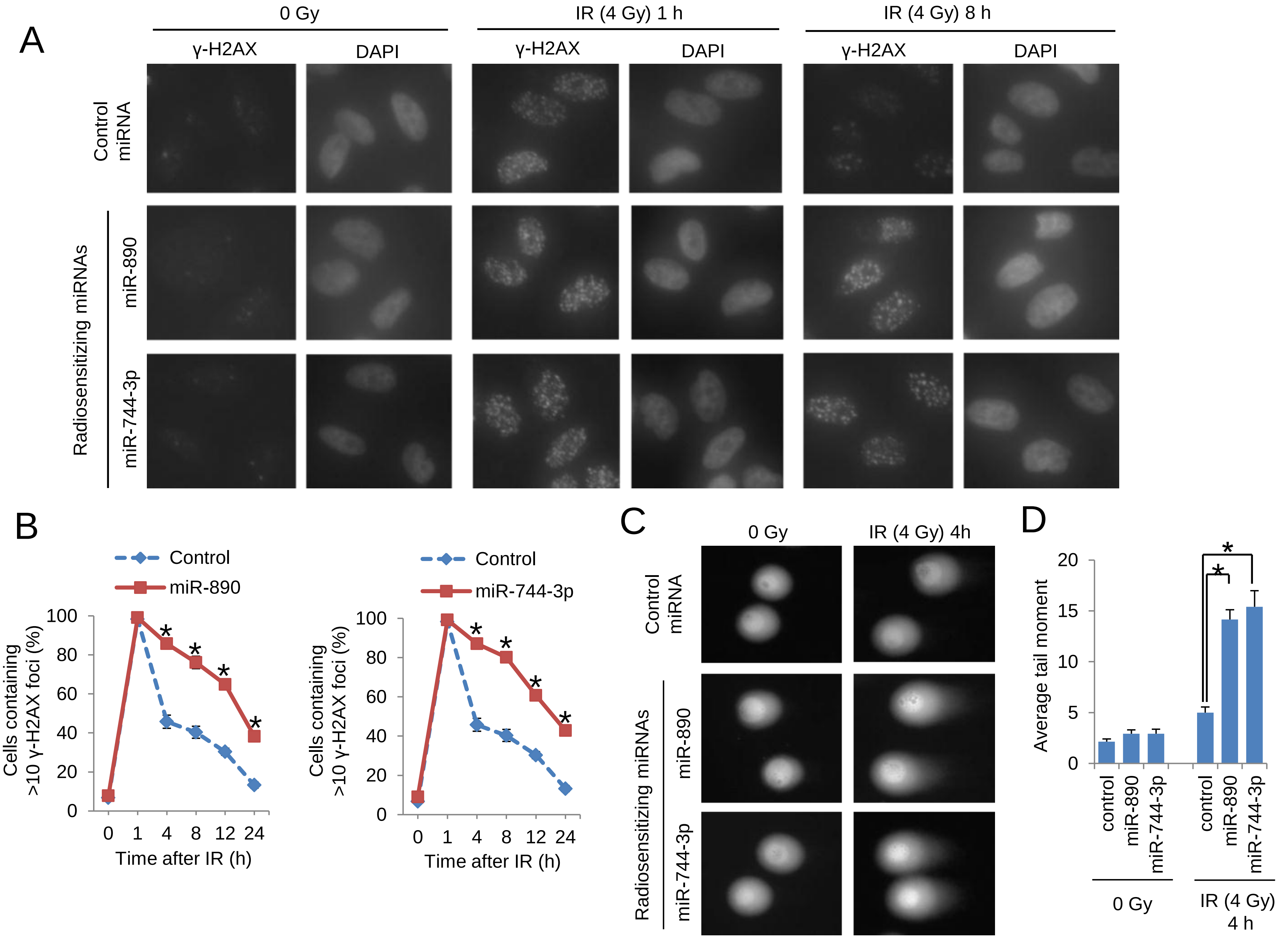

Supplementary Figure 6

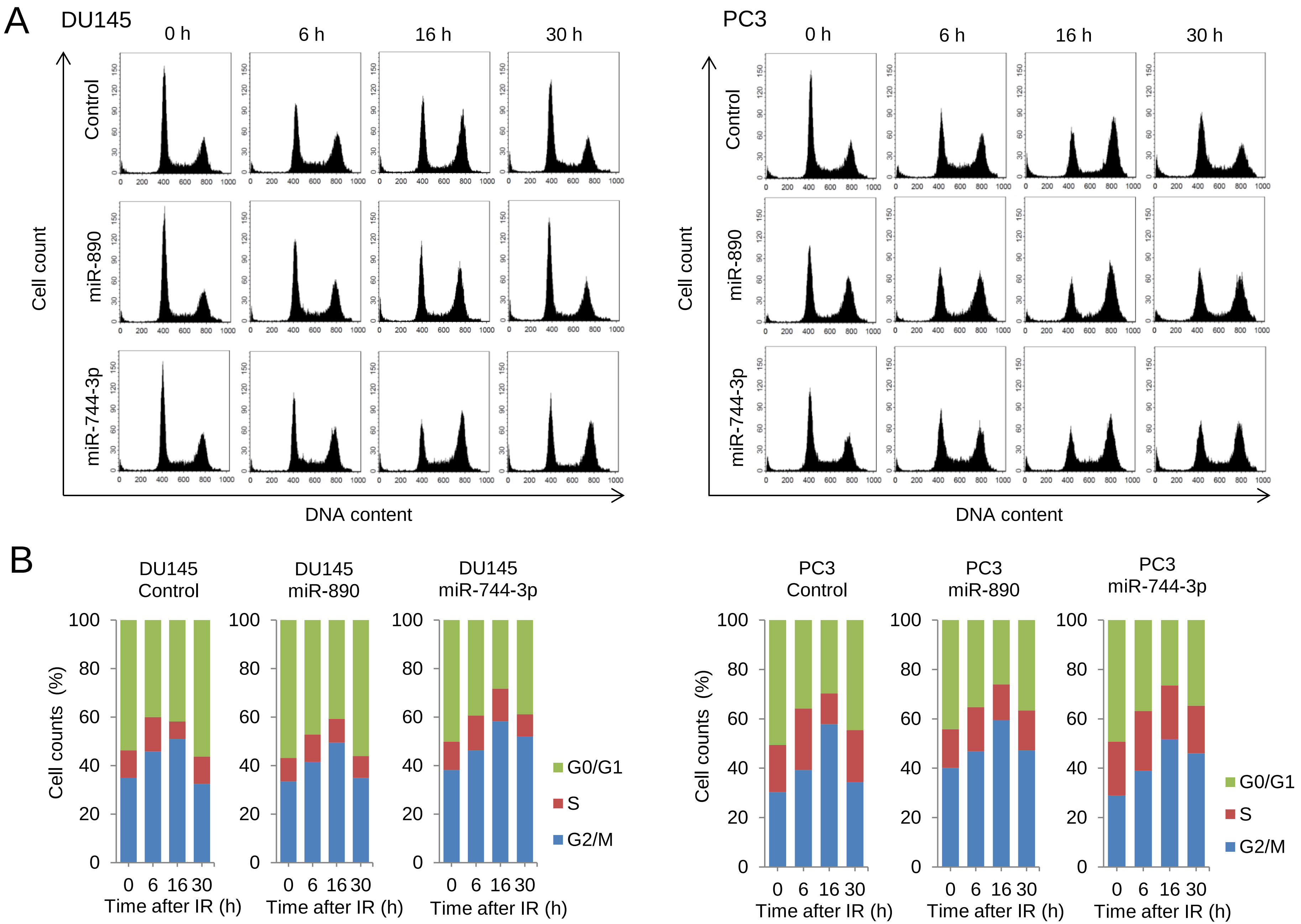

Supplement Figure 7

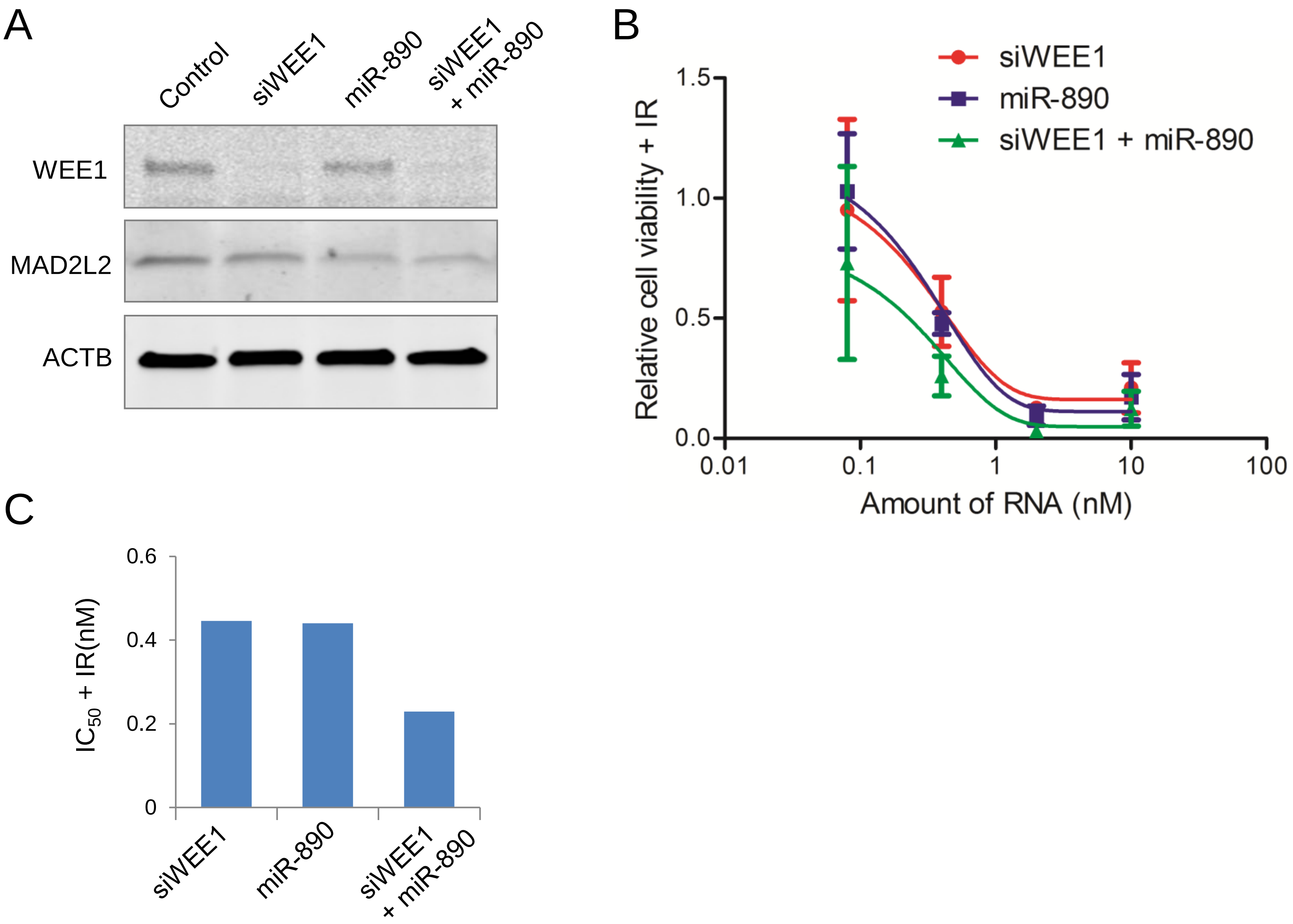

Supplementary Figure 8

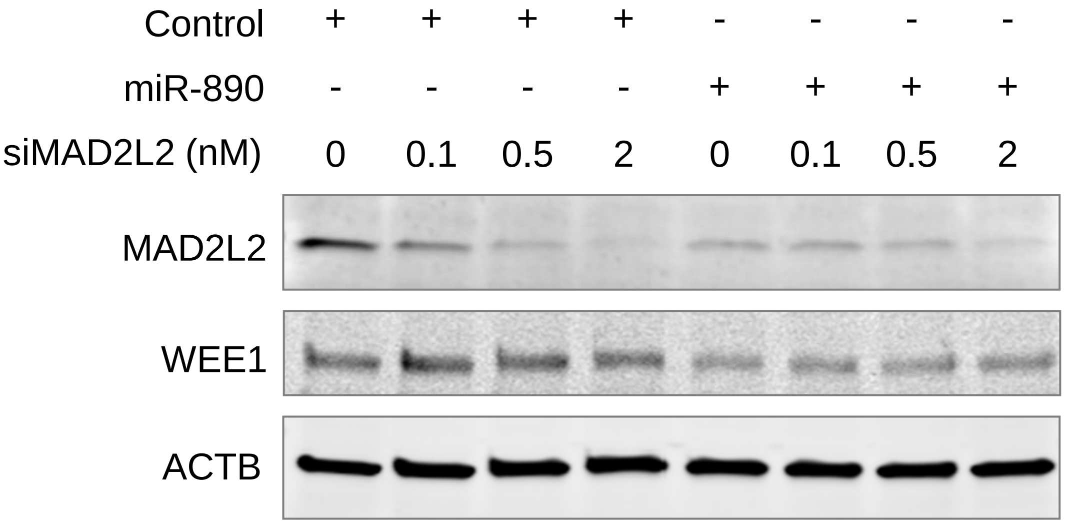

Supplementary Figure 9

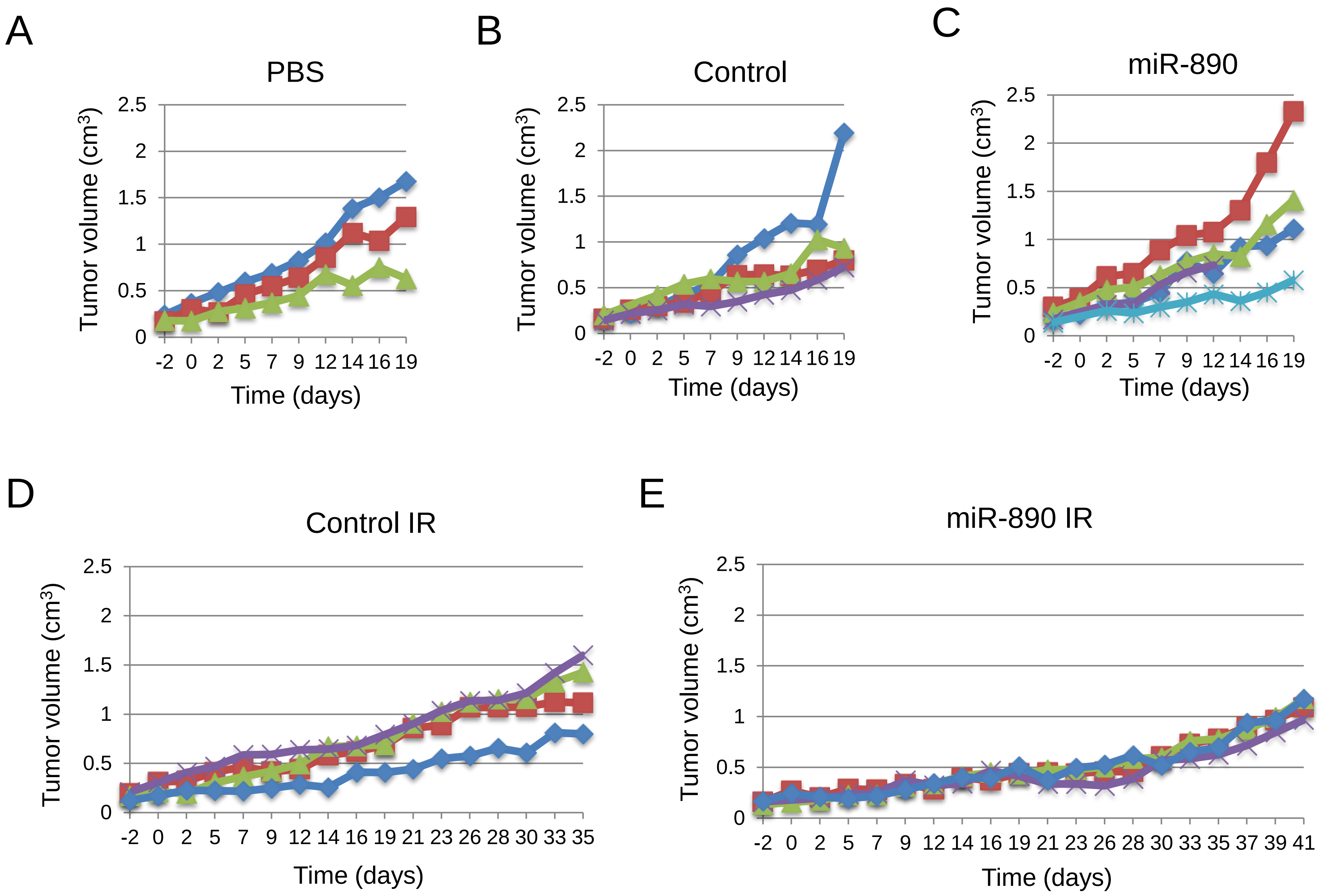

Supplementary Table 1. The sequence of siRNAs used in this study.

siRNA		
Name	sense	anti-sense
DNAPK	UUCGGCUAACUCGCCAGUUUA	UAAACUGGCGAGUUAGCCGAA
MAD2L2	CACCCGGAGCUGAAUCAGUAU	AUACUGAUUCAGCUCCGGGUG
WEE1	CACTGGTAAAGCATTCAGTAT	ATACTGAATGCTTTACCAGTG

Supplementary Table 2. The sequence of primers used in this study.

Primers		
Primers for 3'UTR cloning		
Name	Forward	Reverse
MAD2L2 3'UTR	GGACTAGTCACCCCACTGATGCCCAAAC	CCCAAGCTTGGGGCCCCTTTATTGAAAC
RAD23B 3'UTR	GGACTAGTAAATCAGCTTTTGCAGGTC	CCCAAGCTTGCCTCCCACCCCAAGTATTTA
WEE1 3'UTR	GGACTAGTTTCTGGTAATGTCCTTCCCGGA	CCCAAGCTTGCTCAGAGTGACTTTTAATATGCCA
XPC 3'UTR	GGACTAGTTCATCTGTCCGACAAGTTCA	CCCAAGCTTGCTCCTTAGTACAGAGAGCTTTATAC
KU80 3'UTR	GGACTAGTTTTCTGTGGTCTTACTGATC	CCCAAGCTTGAGGCAGAGTAATGTGGTAAC
XLF 3'UTR	GGACTAGTGGTCCCACCCCATTGTTGTC	CCCAAGCTTGGCAAAGCCTTGTCTCCACA
MCL1 3'UTR	GGACTAGTTTGACTTTTAACCAACCACCACCA	CCCAAGCTTGACTGGCCACTTTCCTGTTCTCA
Primers for mutant pMIR-3'UTR reporter vector		
Name	Forward	Reverse
Mut-MAD2L2	CTGCATGGCTGCCCTGATTTAGAGTGCTCTTATCGC CTC	GAGGCGATAAGAGCACTCTAAATCAGGGCAGCCATGCA G
Mut-RAD23B	CTAGAATTTACAGTCTCTGTTTCATGTAGACACTGGA TAATGGCTTTGTG	CACAAAGCCATTATCCAGTGTCTACATGAAACAGAGACT GTAAATTCTAG

Supplementary Table 3. Summary of the results of the high-throughput miRNA screening for cell growth and IR response. 810 miRNA mimetics were transfected into LNCaP-MLuc cells. After two days, cells were irradiated or non-irradiated, and the relative number of surviving cells was quantified by MLuc assay on day 11 post IR. The MLuc activity, representing relative cell viability, for cells transfected with each miRNA was normalized to those transfected with a control miRNA. Results are categorized as cell growth, for non-irradiated samples, and radiation sensitization, for irradiated samples. Anti-proliferative miRNAs were defined as those which inhibited cell growth by over 50% without IR. IR sensitizing or IR protective miRNAs were defined as those which increased IR induced-cell death by over 50% or increased cell survival by over 2 fold, respectively, excluding anti-proliferative miRNAs.

MicroRNA	miRBase accession number	Sequence	Cell growth normalized to control		IR response normalized to control		Category
			Mean (n=4)	SEM	Mean (n=4)	SEM	
hsa-let-7a	MI0000060	UGAGGUAGUAGGUUGUAUAGUU	0.8041	0.0084	0.5011	0.0124	–
hsa-let-7a	MI0000062	UGAGGUAGUAGGUUGUAUAGUU	0.8539	0.0038	0.6082	0.0791	–
hsa-let-7a	MI0000061	UGAGGUAGUAGGUUGUAUAGUU	1.0826	0.0202	1.5569	0.0473	–
hsa-let-7a*	MI0000060	CUAUACAAUCUACUGUCUUUC	0.9774	0.0624	0.7869	0.0261	–
hsa-let-7a*	MI0000062	CUAUACAAUCUACUGUCUUUC	0.9802	0.0709	1.3474	0.1846	–
hsa-let-7b	MI0000063	UGAGGUAGUAGGUUGUGUGGUU	0.9439	0.0042	0.5533	0.0420	–
hsa-let-7b*	MI0000063	CUAUACAACCUACUGCCUUCCC	0.6899	0.0074	0.3826	0.0070	IR sensitizing
hsa-let-7c	MI0000064	UGAGGUAGUAGGUUGUAUGGUU	1.0407	0.0271	0.9334	0.0421	–
hsa-let-7c*	MI0000064	UAGAGUUACACCCUGGGAGUUA	0.7000	0.0034	0.6052	0.0900	–
hsa-let-7d	MI0000065	AGAGGUAGUAGGUUGCAUAGUU	0.7550	0.0179	0.2740	0.0161	IR sensitizing
hsa-let-7d*	MI0000065	CUAUACGACCUGCUGCCUUUCU	0.7337	0.0123	0.6885	0.1232	–
hsa-let-7e	MI0000066	UGAGGUAGGAGGUUGUAUAGUU	0.8641	0.0204	0.2998	0.0470	IR sensitizing
hsa-let-7e*	MI0000066	CUAUACGGCCUCCUAGCUUUCC	0.8050	0.0187	0.5513	0.0620	–
hsa-let-7f	MI0000067	UGAGGUAGUAGAUUGUAUAGUU	0.8799	0.0152	0.2869	0.0310	IR sensitizing
hsa-let-7f	MI0000068	UGAGGUAGUAGAUUGUAUAGUU	0.7732	0.0135	0.2891	0.0176	IR sensitizing
hsa-let-7f-1*	MI0000067	CUAUACAAUCUAUUGCCUUCCC	0.6366	0.0256	0.1031	0.0117	IR sensitizing
hsa-let-7f-2*	MI0000068	CUAUACAGUCUACUGUCUUUCC	0.7062	0.0067	0.6177	0.0489	–
hsa-let-7g	MI0000433	UGAGGUAGUAGUUUGUACAGUU	0.7924	0.0041	0.2524	0.0251	IR sensitizing
hsa-let-7g*	MI0000433	CUGUACAGGCCACUGCCUUGC	0.1979	0.0551	0.0757	0.0055	Anti-proliferative
hsa-let-7i	MI0000434	UGAGGUAGUAGUUUGUGCUGUU	0.7524	0.0316	0.2350	0.0363	IR sensitizing
hsa-let-7i*	MI0000434	CUGCGCAAGCUACUGCCUUGCU	0.7183	0.0646	0.3437	0.0509	IR sensitizing
hsa-miR-1	MI0000437	UGGAAUGUAAAGAAGUAUGUAU	0.5377	0.0214	0.1247	0.0071	IR sensitizing
hsa-miR-1	MI0000651	UGGAAUGUAAAGAAGUAUGUAU	0.4341	0.0048	0.1000	0.0051	Anti-proliferative
hsa-miR-100	MI0000102	AACCCGUAGAUCCGAACUUGUG	0.8983	0.0174	0.3812	0.0137	IR sensitizing
hsa-miR-100*	MI0000102	CAAGCUUGUAUCUAUAGGUAUG	0.7503	0.0199	2.2564	0.2427	IR protective
hsa-miR-101	MI0000739	UACAGUACUGUGAUAACUGAA	0.6885	0.0202	0.1710	0.0131	IR sensitizing
hsa-miR-101	MI0000103	UACAGUACUGUGAUAACUGAA	0.6846	0.0044	0.4206	0.0536	IR sensitizing
hsa-miR-101*	MI0000103	CAGUUAUCACAGUGCUGAUGCU	0.4609	0.0499	0.0519	0.0042	Anti-proliferative
hsa-miR-103	MI0000109	AGCAGCAUUGUACAGGGCUAUGA	0.7369	0.0019	0.2020	0.0284	IR sensitizing
hsa-miR-103	MI0000108	AGCAGCAUUGUACAGGGCUAUGA	0.7492	0.0633	0.2078	0.0077	IR sensitizing
hsa-miR-105	MI0000112	UCAAAUGCUCAGACUCCUGUGGU	0.8355	0.0106	0.2478	0.0228	IR sensitizing
hsa-miR-105	MI0000111	UCAAAUGCUCAGACUCCUGUGGU	0.8607	0.0051	0.2534	0.0139	IR sensitizing
hsa-miR-105*	MI0000112	ACGGAUGUUUGAGCAUGUGCUA	0.5136	0.0268	0.1447	0.0222	IR sensitizing
hsa-miR-105*	MI0000111	ACGGAUGUUUGAGCAUGUGCUA	0.4246	0.0183	0.1631	0.0044	Anti-proliferative
hsa-miR-106a	MI0000113	AAAAGUGCUUACAGUGCAGGUAG	1.0258	0.0231	1.8400	0.0309	–
hsa-miR-106a*	MI0000113	CUGCAAUGUAAGCACUUCUUAC	0.4557	0.0070	0.0617	0.0040	Anti-proliferative
hsa-miR-106b	MI0000734	UAAAGUGCUGACAGUGCAGAU	1.0790	0.0177	3.9404	0.7860	IR protective
hsa-miR-106b*	MI0000734	CCGCACUGUGGGUACUUGCUGC	0.7654	0.0133	0.6435	0.0177	–
hsa-miR-107	MI0000114	AGCAGCAUUGUACAGGGCUAUCA	0.7571	0.0281	0.3698	0.0131	IR sensitizing
hsa-miR-10a	MI0000266	UACCCUGUAGAUCCGAAUUUGUG	0.7258	0.0527	0.1114	0.0082	IR sensitizing
hsa-miR-10a*	MI0000266	CAAAUUCGUAUCUAGGGGAAUA	0.6891	0.0139	0.3067	0.0652	IR sensitizing
hsa-miR-10b	MI0000267	UACCCUGUAGAACCGAAUUUGUG	0.7509	0.0367	0.4434	0.0913	IR sensitizing
hsa-miR-10b*	MI0000267	ACAGAUUCGAUUCUAGGGGAAU	0.7432	0.0079	0.2153	0.0147	IR sensitizing
hsa-miR-122*	MI0000442	AACGCCAUUAUCACACUAAAUA	0.7535	0.0198	0.3385	0.0118	IR sensitizing
hsa-miR-1224-3p	MI0003764	CCCCACCUCCUCUCUCCUCAG	1.0097	0.0043	2.0936	0.0223	IR protective
hsa-miR-1224-5p	MI0003764	GUGAGGACUCGGGAGGUGG	0.8604	0.0071	1.0604	0.0292	–
hsa-miR-1225-3p	MI0006311	UGAGCCCCUGUGCCGCCCCCAG	0.9533	0.0088	1.3683	0.2551	–
hsa-miR-1225-5p	MI0006311	GUGGGUACGGCCCAGUGGGGGG	0.9619	0.0151	1.7482	0.0148	–
hsa-miR-1226	MI0006313	UCACCAGCCCUGUGUUCCCUAG	0.8137	0.0061	0.1391	0.0102	IR sensitizing
hsa-miR-1226*	MI0006313	GUGAGGGCAUGCAGGCCUGGAUGGGG	0.8862	0.0269	0.4958	0.1015	IR sensitizing
hsa-miR-1227	MI0006316	CGUGCCACCCUUUUCCCCAG	0.9856	0.0040	0.8686	0.0522	–
hsa-miR-1228	MI0006318	UCACACCUGCCUCGCCCCCC	0.7914	0.0192	0.1245	0.0094	IR sensitizing
hsa-miR-1228*	MI0006318	GUGGGCGGGGGCAGGUGUGUG	0.8905	0.0076	1.4090	0.0255	–
hsa-miR-1229	MI0006319	CUCUCACCACUGCCCUCCCACAG	0.9422	0.0279	2.2888	0.0961	IR protective
hsa-miR-122a	MI0000442	UGGAGUGUGACAAUGGUGUUUG	0.3429	0.0173	0.0742	0.0069	Anti-proliferative

hsa-miR-1231	MI0006321	GUGUCUGGGCGGACAGCUGC	0.1999	0.0347	0.1333	0.0222	Anti-proliferative
hsa-miR-1233	MI0006323	UGAGCCCUGUCCUCCCGCAG	0.8152	0.0179	0.4917	0.0721	IR sensitizing
hsa-miR-1234	MI0006324	UCGGCCUGACCACCCACCCCAC	0.8020	0.0128	1.0739	0.0884	—
hsa-miR-1236	MI0006326	CCUCUUCCCCUUGUCUCUCCAG	0.9142	0.0065	2.4281	0.1518	IR protective
hsa-miR-1237	MI0006327	UCCUUCUGCUCCGUCCCCCAG	0.6807	0.0175	0.7607	0.0366	—
hsa-miR-1238	MI0006328	CUUCCUCGUCUGUCUGCCCC	0.7790	0.0123	0.2485	0.0357	IR sensitizing
hsa-miR-124*	MI0000445	CGUGUUCACAGCGGACCUUGAU	0.6167	0.0068	0.1184	0.0272	IR sensitizing
hsa-miR-124*	MI0000443	CGUGUUCACAGCGGACCUUGAU	0.7919	0.0243	0.1527	0.0025	IR sensitizing
hsa-miR-124*	MI0000444	CGUGUUCACAGCGGACCUUGAU	0.5555	0.0763	0.4234	0.0701	IR sensitizing
hsa-miR-124a	MI0000443	UAAGGCACGCGGUGAAUGCC	0.2259	0.0094	0.0813	0.0083	Anti-proliferative
hsa-miR-124a	MI0000445	UAAGGCACGCGGUGAAUGCC	0.2996	0.0328	0.0727	0.0058	Anti-proliferative
hsa-miR-124a	MI0000444	UAAGGCACGCGGUGAAUGCC	0.3045	0.0298	0.0734	0.0048	Anti-proliferative
hsa-miR-125a	MI0000469	UCCCUGAGACCCUUUAACCUGUGA	0.6538	0.0163	1.8782	0.0619	—
hsa-miR-125a-3p	MI0000469	ACAGGUGAGGUUCUUGGGAGCC	0.9197	0.1087	0.7256	0.1533	—
hsa-miR-125b	MI0000446	UCCCUGAGACCCUAACUUGUGA	0.6548	0.0378	1.1348	0.0734	—
hsa-miR-125b	MI0000470	UCCCUGAGACCCUAACUUGUGA	0.6563	0.0152	1.4880	0.2437	—
hsa-miR-125b-1*	MI0000446	ACGGGUUAGGCUCUUGGGAGCU	0.7647	0.0217	0.2458	0.0068	IR sensitizing
hsa-miR-125b-2*	MI0000470	UCACAAGUCAGGCUCUUGGGAC	0.9972	0.1009	4.6357	0.9417	IR protective
hsa-miR-126	MI0000471	UCGUACCGUGAGUAAUAAUGCG	1.0571	0.0117	0.5000	0.1012	IR sensitizing
hsa-miR-126*	MI0000471	CAUUAUUACUUUUGGUACGCG	0.7301	0.0119	0.4610	0.1073	IR sensitizing
hsa-miR-127	MI0000472	UCGGAUCCGUCUGAGCUUGGCU	0.4104	0.0265	0.1072	0.0047	Anti-proliferative
hsa-miR-127-5p	MI0000472	CUGAAGCUCAGAGGGCUCUGAU	0.5921	0.0562	0.2370	0.0483	IR sensitizing
hsa-miR-128	MI0000727	UCACAGUGAACCGGUCUCUUU	0.9270	0.0241	1.3883	0.1277	—
hsa-miR-128a	MI0000447	UCACAGUGAACCGGUCUCUUU	0.8806	0.0043	0.7198	0.0378	—
hsa-miR-129	MI0000473	CUUUUUGCGGUCUGGGCUUGC	0.4263	0.0907	0.1287	0.0145	Anti-proliferative
hsa-miR-129	MI0000252	CUUUUUGCGGUCUGGGCUUGC	0.4323	0.0808	0.0770	0.0096	Anti-proliferative
hsa-miR-129*	MI0000252	AAGCCCUUACCCCAAAAAGUAU	0.5045	0.0348	0.0749	0.0014	IR sensitizing
hsa-miR-129-3p	MI0000473	AAGCCCUUACCCCAAAAAGCAU	0.4807	0.0628	0.1701	0.0302	Anti-proliferative
hsa-miR-130a	MI0000448	CAGUGCAAUGUUAAAAGGGCAU	0.8722	0.0410	2.3713	0.2203	IR protective
hsa-miR-130a*	MI0000448	UUCACAUUGUGCUACUGUCUGC	0.7124	0.0034	0.6782	0.1228	—
hsa-miR-130b	MI0000748	CAGUGCAAUGAUGAAAGGGCAU	0.9770	0.0409	0.3931	0.0257	IR sensitizing
hsa-miR-130b*	MI0000748	ACUCUUUCCCUGUUGCACUAC	0.5371	0.0373	0.0483	0.0024	IR sensitizing
hsa-miR-132	MI0000449	UAACAGUCUACAGCCAUGGUCG	0.8417	0.0321	1.0809	0.1279	—
hsa-miR-132*	MI0000449	ACCGUGGCUUUCGAUUGUUACU	0.3933	0.1653	0.0476	0.0006	Anti-proliferative
hsa-miR-133a	MI0000450	UUUGGUCCCCUUCAACCAGCUG	0.3645	0.0344	0.0888	0.0017	Anti-proliferative
hsa-miR-133a	MI0000451	UUUGGUCCCCUUCAACCAGCUG	0.4087	0.0047	0.1003	0.0117	Anti-proliferative
hsa-miR-133b	MI0000822	UUUGGUCCCCUUCAACCAGCUA	0.2571	0.0057	0.0678	0.0082	Anti-proliferative
hsa-miR-134	MI0000474	UGUGACUGGUUGACCAGAGGGG	0.4863	0.0067	0.1321	0.0061	Anti-proliferative
hsa-miR-135a	MI0000453	UAUGGCUUUUUAUUCCUAUGUGA	0.6617	0.0191	0.0884	0.0055	IR sensitizing
hsa-miR-135a	MI0000452	UAUGGCUUUUUAUUCCUAUGUGA	0.3111	0.0178	0.0489	0.0033	Anti-proliferative
hsa-miR-135a*	MI0000452	UAUAGGGAUUGGAGCCGUGGCG	0.7943	0.0214	0.1906	0.0128	IR sensitizing
hsa-miR-135b	MI0000810	UAUGGCUUUUCAUUCCUAUGUGA	0.2949	0.0387	0.0670	0.0053	Anti-proliferative
hsa-miR-135b*	MI0000810	AUGUAGGGCUAAAAGCCAUGGG	0.3620	0.0599	0.0787	0.0117	Anti-proliferative
hsa-miR-136	MI0000475	ACUCCAUUUGUUUUGAUGAUGGA	0.7618	0.0299	0.2456	0.0668	IR sensitizing
hsa-miR-136*	MI0000475	CAUCAUCGUCUCAAAUGAGUCU	0.2166	0.0284	0.0838	0.0175	Anti-proliferative
hsa-miR-137	MI0000454	UUAUUGCUUAAGAAUACGCGUAG	0.8045	0.0194	0.3669	0.0318	IR sensitizing
hsa-miR-138	MI0000455	AGCUGGUGUUGUGAAUCAGGCCG	0.5387	0.0257	0.2812	0.0111	IR sensitizing
hsa-miR-138	MI0000476	AGCUGGUGUUGUGAAUCAGGCCG	0.4689	0.0079	0.2715	0.0191	Anti-proliferative
hsa-miR-138-1*	MI0000476	GCUACUUCACAACACCAGGGCC	0.9918	0.0800	2.6303	0.6601	IR protective
hsa-miR-138-2*	MI0000455	GCUAUUUCACGACACCAGGGUU	1.0048	0.0143	0.5456	0.0413	—
hsa-miR-139	MI0000261	UCUACAGUGCACGUGUCUCCAG	0.8448	0.0169	1.0647	0.1519	—
hsa-miR-139-3p	MI0000261	GGAGACGCGGCCCUGUUGGAGU	0.6889	0.0195	0.6288	0.1835	—
hsa-miR-140	MI0000456	CAGUGGUUUUACCCUAUGGUAG	0.8117	0.0198	0.2087	0.0146	IR sensitizing
hsa-miR-140-3p	MI0000456	UACCACAGGGUAGAACCACGG	1.1001	0.0102	1.4576	0.1591	—
hsa-miR-141	MI0000457	UAACACUGUCUGGUAAAGAUGG	0.7707	0.0306	0.6462	0.0853	—
hsa-miR-141*	MI0000457	CAUCUUCCAGUACAGUGUUGGA	0.8234	0.0148	0.2189	0.0160	IR sensitizing
hsa-miR-142-3p	MI0000458	UGUAGUGUUUCCUACUUUAUGGA	0.8772	0.0559	0.3713	0.0310	IR sensitizing
hsa-miR-142-5p	MI0000458	CAUAAAGUAGAAAGCACUACU	0.7510	0.0267	1.3318	0.0615	—
hsa-miR-143	MI0000459	UGAGAUGAAGCACUGUAGCUC	0.6571	0.0083	0.1961	0.0102	IR sensitizing
hsa-miR-143*	MI0000459	GGUGCAGUGCUGCAUCUCUGGU	0.8151	0.0034	1.0073	0.0480	—
hsa-miR-144	MI0000460	UACAGUAUAGAUGAUGUACU	0.7265	0.0192	0.4161	0.0165	IR sensitizing
hsa-miR-144*	MI0000460	GGAUAUCAUCAUAUACUGUAAG	0.6358	0.0753	0.2359	0.0087	IR sensitizing
hsa-miR-145	MI0000461	GUCCAGUUUUCCCAGGAAUCCCU	0.6547	0.0132	0.3191	0.0494	IR sensitizing
hsa-miR-145*	MI0000461	GGAUUCCUGGAAAUACUGUUCU	0.8760	0.1298	4.8001	1.5232	IR protective
hsa-miR-146a	MI0000477	UGAGAACUGAAUUCCAUGGGUU	0.7096	0.0566	0.2440	0.0409	IR sensitizing
hsa-miR-146a*	MI0000477	CCUCUGAAAUUCAGUUCUUCAG	1.0002	0.0788	2.2984	0.0838	IR protective
hsa-miR-146b	MI0003129	UGAGAACUGAAUUCCAUAGGCU	0.9191	0.0056	0.2987	0.0301	IR sensitizing
hsa-miR-146b-3p	MI0003129	UGCCCUGUGGACUCAGUUCUGG	0.6385	0.0100	1.6192	0.0912	—

hsa-miR-147	MI0000262	GUGUGUGGAAAUGCUUCUGC	0.7001	0.0178	1.1967	0.0906	—
hsa-miR-147b	MI0005544	GUGUGCGGAAAUGCUUCUGCUA	0.8968	0.0155	0.5854	0.0693	—
hsa-miR-148a	MI0000253	UCAGUGCACUACAGAACUUUGU	0.9991	0.0228	0.9673	0.1019	—
hsa-miR-148a*	MI0000253	AAAGUUCUGAGACACUCCGACU	0.7722	0.0040	0.6838	0.0426	—
hsa-miR-148b	MI0000811	UCAGUGCAUCACAGAACUUUGU	0.8526	0.0268	0.5226	0.0392	—
hsa-miR-148b*	MI0000811	AAGUUCUGUUAUACACUCAGGC	0.5011	0.0154	0.1069	0.0075	IR sensitizing
hsa-miR-149	MI0000478	UCUGGCUCCGUGUCUUCACUCCC	0.8050	0.0072	0.5235	0.0808	—
hsa-miR-149*	MI0000478	AGGGAGGGACGGGGGCUGUGC	0.5380	0.0521	0.2459	0.0263	IR sensitizing
hsa-miR-150	MI0000479	UCUCCCAACCCUUGUACCAGUG	0.7342	0.0352	0.8894	0.1537	—
hsa-miR-150*	MI0000479	CUGGUACAGGCCUGGGGGACAG	0.9845	0.0794	2.6782	0.1093	IR protective
hsa-miR-151	MI0000809	CUAGACUGAAGCUCCUUGAGG	0.7966	0.0158	0.2138	0.0147	IR sensitizing
hsa-miR-151-5p	MI0000809	UCGAGGAGCUCACAGUCUAGU	0.3911	0.0753	0.4896	0.1060	Anti-proliferative
hsa-miR-152	MI0000462	UCAGUGCAUGACAGAACUUGG	0.7439	0.0249	0.5946	0.0321	—
hsa-miR-153	MI0000463	UUGCAUAGUCACAAAAGUGAUC	0.3778	0.0468	0.0520	0.0031	Anti-proliferative
hsa-miR-153	MI0000464	UUGCAUAGUCACAAAAGUGAUC	0.3808	0.0082	0.0443	0.0022	Anti-proliferative
hsa-miR-154	MI0000480	UAGGUUAUCCGUGUUGCCUUCG	0.6328	0.0572	0.1059	0.0180	IR sensitizing
hsa-miR-154*	MI0000480	AAUCAUACACGGUUGACCUAUU	0.7065	0.0268	0.3843	0.0240	IR sensitizing
hsa-miR-155	MI0000681	UUAAUGCUAAUCGUGAUAGGGGU	0.6851	0.0262	0.5770	0.0160	—
hsa-miR-155*	MI0000681	CUCCUACAUAUUAGCAUUAACA	0.7936	0.0132	1.4928	0.0445	—
hsa-miR-15a	MI0000069	UAGCAGCACAUAAUGGUUUGUG	0.3351	0.0077	0.0535	0.0024	Anti-proliferative
hsa-miR-15a*	MI0000069	CAGGCCAUAUUGUGCUGCCUCA	0.6242	0.0152	0.3361	0.0504	IR sensitizing
hsa-miR-15b	MI0000438	UAGCAGCACAUCAUGGUUUACA	0.3339	0.0021	0.0671	0.0088	Anti-proliferative
hsa-miR-15b*	MI0000438	CGAAUCAUUAUUUGCUGCUCUA	1.0552	0.0401	3.2178	0.3787	IR protective
hsa-miR-16	MI0000115	UAGCAGCACGUAAAUAUUGGCG	0.6786	0.1051	0.0842	0.0093	IR sensitizing
hsa-miR-16	MI0000070	UAGCAGCACGUAAAUAUUGGCG	0.2514	0.0840	0.0384	0.0022	Anti-proliferative
hsa-miR-16-1*	MI0000070	CCAGUAUUAACUGUGCUGCUGA	0.6827	0.0119	1.1763	0.2622	—
hsa-miR-16-2*	MI0000115	CCAAUAUUACUGUGCUGCUUUA	0.7163	0.0051	0.4185	0.0152	IR sensitizing
hsa-miR-17-3p	MI0000071	ACUGCAGUGAAGGCACUUGUAG	0.7322	0.0196	0.4307	0.0465	IR sensitizing
hsa-miR-17-5p	MI0000071	CAAAGUGCUUACAGUGCAGGUAG	0.8932	0.0157	1.1356	0.2616	—
hsa-miR-181a	MI0000289	AACAUUCAACGCUGUCGGUGAGU	0.8346	0.0322	0.1738	0.0177	IR sensitizing
hsa-miR-181a	MI0000269	AACAUUCAACGCUGUCGGUGAGU	0.8435	0.0215	0.2398	0.0116	IR sensitizing
hsa-miR-181a-2*	MI0000269	ACCACUGACCGUUGACUGUACC	0.7755	0.0073	1.2687	0.1001	—
hsa-miR-181b	MI0000270	AACAUUCAUUGCUGUCGGUGGGU	0.7842	0.0052	0.5033	0.0442	—
hsa-miR-181b	MI0000683	AACAUUCAUUGCUGUCGGUGGGU	0.7746	0.0150	0.5445	0.0288	—
hsa-miR-181c	MI0000271	AACAUUCAACCUGUCGGUGAGU	0.6438	0.0365	0.2587	0.0165	IR sensitizing
hsa-miR-181c*	MI0000271	AACCAUCGACCGUUGAGUGGAC	0.2524	0.0437	0.2070	0.0393	Anti-proliferative
hsa-miR-181d	MI0003139	AACAUUCAUUGUUGUCGGUGGGU	0.8900	0.0094	0.6879	0.1043	—
hsa-miR-182	MI0000272	UUUGGCAAUGGUAGAACUCACACU	0.7557	0.0060	0.7695	0.0280	—
hsa-miR-182*	MI0000272	UGGUUCUAGACUUGCCAACUA	0.2430	0.0335	0.0757	0.0060	Anti-proliferative
hsa-miR-183	MI0000273	UAUGGCACUGGUAGAAUUCACU	0.2110	0.0294	0.0658	0.0046	Anti-proliferative
hsa-miR-183*	MI0000273	GUGAAUUACCGAAGGGCCAUAA	0.6406	0.0484	0.2805	0.0473	IR sensitizing
hsa-miR-184	MI0000481	UGGACGGAGAACUGAUAAGGGU	0.2049	0.0186	0.0548	0.0043	Anti-proliferative
hsa-miR-185	MI0000482	UGGAGAGAAAGGCAGUUCCUGA	0.1253	0.0106	0.0522	0.0029	Anti-proliferative
hsa-miR-185*	MI0000482	AGGGGCUGGCUUUCCUCUGGUC	0.2913	0.0318	0.0734	0.0040	Anti-proliferative
hsa-miR-186	MI0000483	CAAAGAAUUCUCCUUUUGGGCU	0.7772	0.0138	0.6923	0.0313	—
hsa-miR-186*	MI0000483	GCCCAAAGGUGAAUUUUUUGGG	0.7949	0.0225	1.6348	0.2281	—
hsa-miR-187	MI0000274	UCGUGUCUUGUGUUGCAGCCGG	0.7899	0.0055	0.5550	0.0248	—
hsa-miR-187*	MI0000274	GGCUACAACACAGGACCCGGGC	0.8707	0.0190	1.6240	0.1864	—
hsa-miR-188	MI0000484	CAUCCCUUGCAUGGUGGAGGG	0.8175	0.0086	0.7912	0.0285	—
hsa-miR-188-3p	MI0000484	CUCCCACAUGCAGGGUUUGCA	0.7766	0.0070	1.0087	0.1290	—
hsa-miR-189	MI0000080	UGCCUACUGAGCUGAUAUCAGU	0.6842	0.0038	0.5770	0.1366	—
hsa-miR-18a	MI0000072	UAAGGUGCAUCUAGUGCAGAUAG	0.8138	0.0101	0.3623	0.0374	IR sensitizing
hsa-miR-18a*	MI0000072	ACUGCCCUAAGUGCUCCUUCUGG	0.7276	0.0041	0.6274	0.0584	—
hsa-miR-18b	MI0001518	UAAGGUGCAUCUAGUGCAGUUAG	0.7583	0.0023	0.3197	0.0129	IR sensitizing
hsa-miR-18b*	MI0001518	UGCCCUAAAUGCCCCUUCUGGC	0.6900	0.0183	0.7755	0.1860	—
hsa-miR-190	MI0000486	UGAUAUGUUUGAUAUAUUAGGU	0.6262	0.0414	0.1423	0.0067	IR sensitizing
hsa-miR-190b	MI0005545	UGAUAUGUUUGAUAUUGGGUU	0.6573	0.0498	0.2189	0.0382	IR sensitizing
hsa-miR-191	MI0000465	CAACGGAAUCCCAAAAGCAGCUG	0.7225	0.0109	0.4522	0.0129	IR sensitizing
hsa-miR-191*	MI0000465	GCUGCGCUUGGAUUUCGUCCCC	1.1864	0.0273	3.2168	0.6197	IR protective
hsa-miR-192	MI0000234	CUGACCUAUGAAUUGACAGCC	0.9028	0.0381	0.3292	0.0140	IR sensitizing
hsa-miR-192*	MI0000234	CUGCCAAUUCCAUAGGUCACAG	0.7635	0.0169	0.4231	0.1182	IR sensitizing
hsa-miR-193a	MI0000487	AACUGGCCUACAAAGUCCCAGU	0.1215	0.0321	0.0461	0.0067	Anti-proliferative
hsa-miR-193a-5p	MI0000487	UGGGUCUUUGCGGGCGAGAUGA	0.5396	0.0384	0.2588	0.0436	IR sensitizing
hsa-miR-193b	MI0003137	AACUGGCCCUCAAAGUCCCGCU	0.4375	0.0343	0.1433	0.0455	Anti-proliferative
hsa-miR-193b*	MI0003137	CGGGGUUUUGAGGGCGAGAUGA	0.2389	0.0341	0.1880	0.0249	Anti-proliferative
hsa-miR-194	MI0000732	UGUAACAGCAACUCCAUGUGGA	0.7185	0.0080	0.6237	0.0234	—
hsa-miR-194	MI0000488	UGUAACAGCAACUCCAUGUGGA	0.8403	0.0172	0.7118	0.1351	—
hsa-miR-194*	MI0000732	CCAGUGGGGCUGCUGUUAUCUG	0.6732	0.0151	0.5545	0.0093	—

hsa-miR-195	MI0000489	UAGCAGCACAGAAAUAUUGGC	0.1668	0.0159	0.0442	0.0026	Anti-proliferative
hsa-miR-195*	MI0000489	CCAAUAUUGGCUGUGCUGCUCC	0.9332	0.0378	2.1295	0.4623	IR protective
hsa-miR-196a	MI0000279	UAGGUAGUUUCAUGUUGUUGGG	0.6660	0.0378	0.1354	0.0164	IR sensitizing
hsa-miR-196a	MI0000238	UAGGUAGUUUCAUGUUGUUGGG	0.5845	0.0247	0.1661	0.0127	IR sensitizing
hsa-miR-196a*	MI0000279	CGGCAACAAGAAACUGCCUGAG	0.7403	0.0076	0.1755	0.0166	IR sensitizing
hsa-miR-196b	MI0001150	UAGGUAGUUUCCUGUUGUUGGG	0.5182	0.0334	0.1262	0.0059	IR sensitizing
hsa-miR-197	MI0000239	UUCACCACCUUCUCCACCCAGC	0.5603	0.0530	0.1521	0.0164	IR sensitizing
hsa-miR-198	MI0000240	GGUCCAGAGGGGAGAUAGGUUC	0.7814	0.0131	0.6914	0.0389	—
hsa-miR-199a	MI0000242	CCCAGUGUUCAGACUACCUGUUC	0.8547	0.0077	0.3023	0.0440	IR sensitizing
hsa-miR-199a	MI0000281	CCCAGUGUUCAGACUACCUGUUC	0.8296	0.0149	0.4195	0.0587	IR sensitizing
hsa-miR-199a*	MI0000242	ACAGUAGUCUGCACAUUGGUUA	0.6963	0.0108	0.1556	0.0325	IR sensitizing
hsa-miR-199a*	MI0000281	ACAGUAGUCUGCACAUUGGUUA	0.6344	0.0055	0.1793	0.0183	IR sensitizing
hsa-miR-199b	MI0000282	CCCAGUGUUUAGACUAUCUGUUC	0.9481	0.0170	0.8395	0.2199	—
hsa-miR-199b-3p	MI0000282	ACAGUAGUCUGCACAUUGGUUA	0.6038	0.0069	0.1767	0.0046	IR sensitizing
hsa-miR-19a	MI0000073	UGUGCAAAUCUAUGCAAAACUGA	1.1559	0.0166	2.1580	0.1567	IR protective
hsa-miR-19a*	MI0000073	AGUUUUGCAUAGUUGCACUACA	0.7516	0.0060	0.1632	0.0471	IR sensitizing
hsa-miR-19b	MI0000075	UGUGCAAAUCCAUGCAAAACUGA	0.7908	0.0033	0.5063	0.0641	—
hsa-miR-19b	MI0000074	UGUGCAAAUCCAUGCAAAACUGA	1.1556	0.0158	1.7764	0.0951	—
hsa-miR-19b-1*	MI0000074	AGUUUUGCAGGUUUGCAUCCAGC	0.3064	0.0389	0.0762	0.0171	Anti-proliferative
hsa-miR-19b-2*	MI0000075	AGUUUUGCAGGUUUGCAUUUCA	0.7974	0.0117	0.2561	0.0341	IR sensitizing
hsa-miR-200a	MI0000737	UAACACUGUCUGGUAACGAUGU	0.7191	0.0029	0.8654	0.0582	—
hsa-miR-200a*	MI0000737	CAUCUUACCGGACAGUGCUGGA	0.5586	0.0108	0.1723	0.0154	IR sensitizing
hsa-miR-200b	MI0000342	UAAUACUGCCUGGUAAUGAUGA	0.6465	0.0129	1.1028	0.1877	—
hsa-miR-200b*	MI0000342	CAUCUUACUGGGCAGCAUUGGA	1.0400	0.0244	1.0418	0.3028	—
hsa-miR-200c	MI0000650	UAAUACUGCCGGGUAAUGAUGGA	0.7223	0.0103	0.6592	0.0337	—
hsa-miR-200c*	MI0000650	CGUCUUACCCAGCAGUGUUUGG	0.7621	0.0340	0.5579	0.0416	—
hsa-miR-202	MI0003130	AGAGGUAUAGGGCAUGGGAA	0.7818	0.0070	0.8630	0.2861	—
hsa-miR-202*	MI0003130	UUCCUAUGCAUAUACUUCUUUG	0.7548	0.0287	3.0340	0.4219	IR protective
hsa-miR-203	MI0000283	GUGAAAUGUUUAGGACCACUAG	0.8830	0.0041	0.4633	0.0239	IR sensitizing
hsa-miR-204	MI0000284	UUCCCUUUGUCAUCCUAUGCCU	0.6626	0.0227	0.3020	0.0141	IR sensitizing
hsa-miR-205	MI0000285	UCCUUCAUUCCACCGGAGUCUG	0.7803	0.0066	1.6478	0.0849	—
hsa-miR-206	MI0000490	UGGAAUGUAAGGAAGUGUGUGG	0.4253	0.0190	0.1063	0.0066	Anti-proliferative
hsa-miR-208	MI0000251	AUAAGACGAGCAAAAAGCUUGU	0.5488	0.0272	0.1825	0.0082	IR sensitizing
hsa-miR-208b	MI0005570	AUAAGACGAACAAAAGGUUUGU	0.2667	0.0027	0.0636	0.0043	Anti-proliferative
hsa-miR-20a	MI0000076	UAAAGUGCUUAUAGUGCAGGUAG	1.1360	0.0162	3.8424	0.4255	IR protective
hsa-miR-20a*	MI0000076	ACUGCAUUAUGAGCACUUAAAG	0.6851	0.0125	1.3882	0.0696	—
hsa-miR-20b	MI0001519	CAAAGUGCUCAUAGUGCAGGUAG	1.0941	0.0300	4.0617	0.8014	IR protective
hsa-miR-20b*	MI0001519	ACUGUAGUAUGGGCACUUCCAG	0.5879	0.0178	0.2601	0.0359	IR sensitizing
hsa-miR-21	MI0000077	UAGCUUAUCAGACUGAUGUUGA	1.0929	0.0223	2.8244	0.0922	IR protective
hsa-miR-21*	MI0000077	CAACACCAGUCGAUGGGCUGU	0.5042	0.0336	0.2441	0.0448	IR sensitizing
hsa-miR-210	MI0000286	CUGUGCGUGUGACAGCGGCUGA	0.7864	0.0474	0.4375	0.0263	IR sensitizing
hsa-miR-211	MI0000287	UUCCCUUUGUCAUCCUUCGCCU	0.8553	0.0486	0.6871	0.1291	—
hsa-miR-212	MI0000288	UAACAGUCUCCAGUCACGGCC	0.8810	0.0179	0.4752	0.0426	IR sensitizing
hsa-miR-213	MI0000289	ACCAUCGACCGUUGAUUGUACC	0.9688	0.0245	2.2163	0.2105	IR protective
hsa-miR-214	MI0000290	ACAGCAGGCACAGACAGGCAGU	0.6902	0.0142	0.2366	0.0664	IR sensitizing
hsa-miR-214*	MI0000290	UGCCUGUCUACACUUGCUGUGC	0.1295	0.0073	0.0768	0.0065	Anti-proliferative
hsa-miR-215	MI0000291	AUGACCUAUGAAUUGACAGAC	0.6567	0.0137	0.2648	0.0078	IR sensitizing
hsa-miR-216	MI0000292	UAAUCUCAGCUGGCAACUGUGA	0.8020	0.0089	0.3836	0.0289	IR sensitizing
hsa-miR-216b	MI0005569	AAAUCUCUGCAGGCAAAUGUGA	0.7647	0.0067	0.2287	0.0250	IR sensitizing
hsa-miR-217	MI0000293	UACUGCAUCAGGAACUGAUUGGA	0.5067	0.0140	0.1320	0.0261	IR sensitizing
hsa-miR-218	MI0000295	UUGUGCUUGAUCUAACCAUGU	0.8539	0.0240	0.5689	0.0309	—
hsa-miR-218	MI0000294	UUGUGCUUGAUCUAACCAUGU	0.6356	0.0189	0.4202	0.0102	IR sensitizing
hsa-miR-218-1*	MI0000294	AUGGUUCCGUCAAGCACCAUGG	0.6901	0.0238	0.2256	0.0307	IR sensitizing
hsa-miR-218-2*	MI0000295	CAUGGUUCUGUCAAGCACCGCG	0.7913	0.0028	0.2207	0.0329	IR sensitizing
hsa-miR-219	MI0000740	UGAUUGUCCAAACGCAAUUCU	0.6526	0.0099	0.2798	0.0035	IR sensitizing
hsa-miR-219	MI0000296	UGAUUGUCCAAACGCAAUUCU	0.7938	0.0245	0.3055	0.0325	IR sensitizing
hsa-miR-219-1-3p	MI0000296	AGAGUUGAGUCUGGACGUCCCG	0.1430	0.0109	0.1022	0.0047	Anti-proliferative
hsa-miR-219-2-3p	MI0000740	AGAAUUGUGGCUGGACAUCUGU	0.8604	0.0581	0.7319	0.1978	—
hsa-miR-22	MI0000078	AAGCUGCCAGUUGAAGAACUGU	0.3114	0.0920	0.1175	0.0106	Anti-proliferative
hsa-miR-22*	MI0000078	AGUUCUUCAGUGGCAAGCUUUA	0.5484	0.0061	0.2162	0.0522	IR sensitizing
hsa-miR-220	MI0000297	CCACACCGUAUCUGACACUUU	0.9293	0.0278	3.2974	0.6668	IR protective
hsa-miR-220b	MI0005529	CCACCACCGUGUCUGACACUU	0.9062	0.0086	2.7272	0.4429	IR protective
hsa-miR-220c	MI0005536	ACACAGGGCUGUUGUGAAGACU	0.6362	0.0409	0.0798	0.0107	IR sensitizing
hsa-miR-221	MI0000298	AGCUACAUUGUCUGCUGGGUUUC	0.6896	0.0074	0.6464	0.0328	—
hsa-miR-221*	MI0000298	ACCUGGCAUACAAUGUAGAUUU	0.5800	0.0598	0.1428	0.0052	IR sensitizing
hsa-miR-222	MI0000299	AGCUACAUCUGGCUACUGGGU	0.7901	0.0055	0.5577	0.0257	—
hsa-miR-222*	MI0000299	CUCAGUAGCCAGUGUAGAUCCU	0.4253	0.0291	0.0922	0.0017	Anti-proliferative
hsa-miR-223	MI0000300	UGUCAGUUUGUCAAAUACCCCA	0.7472	0.0028	0.5337	0.0551	—

hsa-miR-223*	MI0000300	CGUGUAUUUGACAAGCUGAGUU	0.6696	0.0031	0.1172	0.0158	IR sensitizing
hsa-miR-224	MI0000301	CAAGUCACUAGUGGUUCCGUU	0.7441	0.0265	0.3164	0.0334	IR sensitizing
hsa-miR-23a	MI0000079	AUCACAUUGCCAGGGAUUUCC	1.0995	0.0133	3.2699	0.3492	IR protective
hsa-miR-23a*	MI0000079	GGGGUUCCUGGGGAUGGGAUUU	0.8400	0.0076	2.0516	0.4321	IR protective
hsa-miR-23b	MI0000439	AUCACAUUGCCAGGGAUUACC	0.6320	0.0190	1.1405	0.0289	—
hsa-miR-23b*	MI0000439	UGGGUUCCUGGCAUGCUGAUUU	0.5624	0.0226	0.1454	0.0332	IR sensitizing
hsa-miR-24	MI0000081	UGGCUCAGUUCAGCAGGAACAG	0.6465	0.1565	0.1272	0.0088	IR sensitizing
hsa-miR-24	MI0000080	UGGCUCAGUUCAGCAGGAACAG	0.4347	0.0211	0.0988	0.0049	Anti-proliferative
hsa-miR-24-2*	MI0000081	UGCCUACUGAGCUGAAACACAG	0.7707	0.0094	0.4175	0.1048	IR sensitizing
hsa-miR-25	MI0000082	CAUUGCACUUGUCUCGGUCUGA	0.9853	0.0157	0.7765	0.0214	—
hsa-miR-25*	MI0000082	AGGCGGAGACUUGGGCAAUUG	0.1479	0.0294	0.0311	0.0005	Anti-proliferative
hsa-miR-26a	MI0000750	UUCAAGUAAUCCAGGAUAGGCU	0.3712	0.0566	0.0602	0.0013	Anti-proliferative
hsa-miR-26a	MI0000083	UUCAAGUAAUCCAGGAUAGGCU	0.4137	0.0124	0.1526	0.0255	Anti-proliferative
hsa-miR-26a-1*	MI0000083	CCUAUUCUUGGUUACUUGCACG	0.8842	0.0107	3.8351	0.5408	IR protective
hsa-miR-26a-2*	MI0000750	CCUAUUCUUGAUUACUUGUUUC	0.9425	0.0240	3.5034	0.7091	IR protective
hsa-miR-26b	MI0000084	UUCAAGUAAUUCAGGAUAGGU	0.7972	0.0182	0.3590	0.0238	IR sensitizing
hsa-miR-26b*	MI0000084	CCUGUUCUCCAUUACUUGGCUC	0.7031	0.0184	0.3014	0.0156	IR sensitizing
hsa-miR-27a	MI0000085	UUCACAGUGGCUAAGUUCCGC	0.8556	0.0157	0.4383	0.0254	IR sensitizing
hsa-miR-27a*	MI0000085	AGGGCUUAGCUGCUUGUGAGCA	0.5316	0.0114	0.0932	0.0064	IR sensitizing
hsa-miR-27b	MI0000440	UUCACAGUGGCUAAGUUCUGC	0.6795	0.0270	0.2697	0.0285	IR sensitizing
hsa-miR-27b*	MI0000440	AGAGCUUAGCUGAUUGGUGAAC	1.0592	0.0147	0.6064	0.0457	—
hsa-miR-28	MI0000086	AAGGAGCUCACAGUCUAUUGAG	0.6853	0.0033	0.1220	0.0147	IR sensitizing
hsa-miR-28-3p	MI0000086	CACUAGAUUGUGAGCUCCUGGA	0.4953	0.0614	0.1655	0.0028	Anti-proliferative
hsa-miR-296	MI0000747	AGGGCCCCCCCUCAAUCCUGU	0.5186	0.0124	0.0988	0.0070	IR sensitizing
hsa-miR-296-3p	MI0000747	GAGGGUUGGGUGGAGGCUCUCC	0.7414	0.0187	0.7000	0.0628	—
hsa-miR-297	MI0005775	AUGUAUGUGUGCAUGUGCAUG	0.5704	0.0056	0.0825	0.0047	IR sensitizing
hsa-miR-298	MI0005523	AGCAGAAGCAGGGAGGUUCUCCCA	0.8404	0.0139	0.3962	0.0450	IR sensitizing
hsa-miR-299-3p	MI0000744	UAUGUGGGAUGGUAAACCGCUU	0.1463	0.0319	0.0570	0.0065	Anti-proliferative
hsa-miR-299-5p	MI0000744	UGGUUUACCGUCCCACAUACAU	0.3067	0.0187	0.0869	0.0044	Anti-proliferative
hsa-miR-29a	MI0000087	UAGCACCAUCUGAAAUCGGUUA	0.4487	0.0227	0.0832	0.0126	Anti-proliferative
hsa-miR-29a*	MI0000087	ACUGAUUUCUUUUGGUGUUCAG	0.8005	0.0248	0.7675	0.0362	—
hsa-miR-29b	MI0000105	UAGCACCAUUUGAAAUCAGUGUU	0.8178	0.0055	1.8440	0.0362	—
hsa-miR-29b	MI0000107	UAGCACCAUUUGAAAUCAGUGUU	0.7936	0.0088	1.8495	0.0260	—
hsa-miR-29b-1*	MI0000105	GCUGGUUUCAUAUGGUGGUUUAGA	0.7185	0.0237	0.3630	0.0345	IR sensitizing
hsa-miR-29b-2*	MI0000107	CUGGUUUCACAUGGUGGCUUAG	0.7176	0.0179	0.2194	0.0309	IR sensitizing
hsa-miR-29c	MI0000735	UAGCACCAUUUGAAAUCGGUUA	0.3554	0.0177	0.0598	0.0066	Anti-proliferative
hsa-miR-29c*	MI0000735	UGACCGAUUUCUCCUGGUGUUC	0.6483	0.0026	0.2151	0.0364	IR sensitizing
hsa-miR-300	MI0005525	UAUACAAGGGCAGACUCUCUCU	0.7892	0.0117	0.8655	0.2202	—
hsa-miR-301	MI0000745	CAGUGCAAUAGUAUUGUCAAAGC	0.7906	0.0278	0.1390	0.0044	IR sensitizing
hsa-miR-301b	MI0005568	CAGUGCAAUGAUAUUGUCAAAGC	0.9298	0.0053	0.4716	0.0266	IR sensitizing
hsa-miR-302a	MI0000738	UAAGUGCUUCCAUGUUUUGGUGA	0.7384	0.0423	1.1324	0.0790	—
hsa-miR-302a*	MI0000738	ACUUAAACGUGGAUGUACUUGCU	0.9927	0.0200	2.1893	0.0699	IR protective
hsa-miR-302b	MI0000772	UAAGUGCUUCCAUGUUUUAGUAG	0.7365	0.0035	0.9753	0.0055	—
hsa-miR-302b*	MI0000772	ACUUUAACAUGGAAGUGCUUUC	0.9703	0.0214	2.0736	0.0901	IR protective
hsa-miR-302c	MI0000773	UAAGUGCUUCCAUGUUUCAGUGG	0.7387	0.0094	0.9733	0.0115	—
hsa-miR-302c*	MI0000773	UUUAACAUGGGGGUACCUGCUG	0.7561	0.0067	1.1662	0.0394	—
hsa-miR-302d	MI0000774	UAAGUGCUUCCAUGUUUGAGUGU	0.7358	0.0040	0.9863	0.0740	—
hsa-miR-302d*	MI0000774	ACUUUAACAUGGAGGCACUUGC	0.8092	0.0071	0.5702	0.0112	—
hsa-miR-30a-3p	MI0000088	CUUUCAGUCGGAUGUUUGCAGC	0.8890	0.0120	0.2354	0.0667	IR sensitizing
hsa-miR-30a-5p	MI0000088	UGUAAACAUCCUCGACUGGAAG	0.3342	0.0422	0.0940	0.0070	Anti-proliferative
hsa-miR-30b	MI0000441	UGUAAACAUCCUACACUCAGCU	0.4822	0.0016	0.1182	0.0083	Anti-proliferative
hsa-miR-30b*	MI0000441	CUGGGAGGUGGAUGUUUACUUC	0.1087	0.0184	0.0585	0.0061	Anti-proliferative
hsa-miR-30c	MI0000736	UGUAAACAUCCUACACUCUCAGC	0.9633	0.0112	3.1998	0.5900	IR protective
hsa-miR-30c	MI0000254	UGUAAACAUCCUACACUCUCAGC	0.4579	0.0718	0.1150	0.0123	Anti-proliferative
hsa-miR-30c-1*	MI0000736	CUGGGAGAGGGUUGUUUACUCC	0.2166	0.0078	0.0648	0.0044	Anti-proliferative
hsa-miR-30c-2*	MI0000254	CUGGGAGAAGGCUGUUUACUCU	0.2768	0.0148	0.0993	0.0331	Anti-proliferative
hsa-miR-30d	MI0000255	UGUAAACAUCCCCGACUGGAAG	0.5156	0.0186	0.2170	0.0104	IR sensitizing
hsa-miR-30d*	MI0000255	CUUUCAGUCAGAUGUUUGCUGC	0.7844	0.0264	0.2260	0.0453	IR sensitizing
hsa-miR-30e-3p	MI0000749	CUUUCAGUCGGAUGUUUACAGC	0.7817	0.0022	0.6186	0.0902	—
hsa-miR-30e-5p	MI0000749	UGUAAACAUCCUUGACUGGAAG	0.6875	0.0411	0.1747	0.0221	IR sensitizing
hsa-miR-31	MI0000089	AGGCAAGAUGCUGGCAUAGCU	0.7208	0.0090	0.5533	0.0919	—
hsa-miR-31*	MI0000089	UGCUAUGCCAACAUAUUGCCAU	0.5977	0.0065	2.3161	0.4223	IR protective
hsa-miR-32	MI0000090	UAUUGCACAUUACUAAGUUGCA	0.8066	0.0069	0.9308	0.0397	—
hsa-miR-32*	MI0000090	CAAUUUAGUGUGUGUGAUAUUU	0.5427	0.0096	0.0634	0.0029	IR sensitizing
hsa-miR-320	MI0000542	AAAAGCUGGGUUGAGAGGGCGA	0.6822	0.0025	0.5645	0.1116	—
hsa-miR-323	MI0000807	CACAUUACACGGUCGACCUCU	0.6604	0.0280	0.0424	0.0022	IR sensitizing
hsa-miR-323-5p	MI0000807	AGGUGGUCCGUGGCGCGUUCGC	0.2898	0.0288	0.1457	0.0357	Anti-proliferative
hsa-miR-324-3p	MI0000813	ACUGCCCCAGGUGCUGCUGG	0.6698	0.0102	0.6562	0.0889	—

hsa-miR-324-5p	MI0000813	CGCAUCCCCUAGGGCAUUGGUGU	1.0619	0.0339	2.6122	0.0979	IR protective
hsa-miR-325	MI0000824	CCUAGUAGGUGUCCAGUAAGUGU	1.0623	0.0178	2.9418	0.3339	IR protective
hsa-miR-326	MI0000808	CCUCUGGGCCCUUCCUCCAG	0.7853	0.0140	0.1356	0.0288	IR sensitizing
hsa-miR-328	MI0000804	CUGGCCCUCUCUGCCCUUCCGU	0.8115	0.0068	0.2910	0.0496	IR sensitizing
hsa-miR-329	MI0001726	AACACACCUGGUUAACCUCUUU	1.1717	0.0574	0.1373	0.0234	IR sensitizing
hsa-miR-329	MI0001725	AACACACCUGGUUAACCUCUUU	0.9473	0.0378	0.2880	0.0215	IR sensitizing
hsa-miR-33	MI0000091	GUGCAUUGUAGUUGCAUUGCA	0.6470	0.0034	0.4414	0.0691	IR sensitizing
hsa-miR-330	MI0000803	GCAAAGCACACGGCCUGCAGAGA	0.9970	0.0112	1.1123	0.2400	–
hsa-miR-330-5p	MI0000803	UCUCUGGGCCUGUGUCUUAGGC	0.5071	0.0536	0.1645	0.0253	IR sensitizing
hsa-miR-331	MI0000812	GCCCCUGGGCCUAUCCUAGAA	0.7634	0.0016	1.6484	0.0252	–
hsa-miR-331-5p	MI0000812	CUAGGUAUGGUCCCAGGGAUCC	0.9467	0.0235	0.8258	0.0365	–
hsa-miR-335	MI0000816	UCAAGAGCAAUAACGAAAAAUGU	0.8764	0.0068	1.9688	0.1764	–
hsa-miR-335*	MI0000816	UUUUUCAUUAUUGCUCCUGACC	0.7115	0.0192	0.1986	0.0168	IR sensitizing
hsa-miR-337	MI0000806	CUCCUAUAUGAUGCCUUUCUUC	0.7345	0.0181	0.9585	0.1114	–
hsa-miR-337-5p	MI0000806	GAACGGCUUCAUACAGGAGUU	0.8500	0.0075	0.7441	0.0795	–
hsa-miR-338	MI0000814	UCCAGCAUCAGUGAUUUUGUUG	0.7133	0.0172	0.3240	0.0858	IR sensitizing
hsa-miR-338-5p	MI0000814	AACAAUAUCCUGGUGCUGAGUG	0.8295	0.0077	1.4913	0.0986	–
hsa-miR-339	MI0000815	UCCCUGUCCUCCAGGAGCUCACG	0.8039	0.0098	0.4180	0.0150	IR sensitizing
hsa-miR-339-3p	MI0000815	UGAGCGCCUCGACGACAGAGCCG	0.4785	0.0148	0.2259	0.0177	Anti-proliferative
hsa-miR-33a*	MI0000091	CAAUGUUUCCACAGUGCAUCAC	0.8404	0.0405	0.4117	0.0195	IR sensitizing
hsa-miR-33b	MI0003646	GUGCAUUGCUGUUGCAUUGC	0.6425	0.0055	0.3032	0.0506	IR sensitizing
hsa-miR-33b*	MI0003646	CAGUGCCUCGGCAGUGCAGCCC	0.9768	0.0080	1.7499	0.1021	–
hsa-miR-340	MI0000802	UUAUAAAGCAAUGAGACUGAUU	0.6925	0.0137	0.2293	0.0398	IR sensitizing
hsa-miR-340*	MI0000802	UCCGUCUCAGUUACUUUAUAGC	0.6372	0.0056	0.1367	0.0135	IR sensitizing
hsa-miR-342	MI0000805	UCUCACACAGAAAUCGCACCCGU	0.7812	0.0182	1.6120	0.0474	–
hsa-miR-342-5p	MI0000805	AGGGGUGCUAUCUGUGAUUGA	0.0672	0.0024	0.1026	0.0143	Anti-proliferative
hsa-miR-345	MI0000825	GCUGACUCCUAGUCCAGGGCUC	1.0891	0.0598	5.5600	0.7885	IR protective
hsa-miR-346	MI0000826	UGUCUGCCCGCAUGCCUGCCUCU	0.8484	0.0198	0.3433	0.0612	IR sensitizing
hsa-miR-34a	MI0000268	UGGCAGUGUCUUAGCUGGUUGU	0.2667	0.0304	0.0891	0.0151	Anti-proliferative
hsa-miR-34a*	MI0000268	CAAUCAGCAAGUAUACUGCCCU	0.7196	0.0242	0.0680	0.0147	IR sensitizing
hsa-miR-34b	MI0000742	CAAUCACUAACUCCACUGCCAU	0.7942	0.0396	0.9920	0.1351	–
hsa-miR-34b*	MI0000742	UAGGCAGUGUCAUUAGCUGAUUG	0.1111	0.0293	0.0598	0.0018	Anti-proliferative
hsa-miR-34c	MI0000743	AGGCAGUGUAGUUAGCUGAUUGC	0.0338	0.0095	0.0307	0.0032	Anti-proliferative
hsa-miR-34c-3p	MI0000743	AAUCACUAACCACACGGCCAGG	0.8070	0.0194	1.1380	0.1890	–
hsa-miR-361	MI0000760	UUAUCAGAAUCUCCAGGGGUAC	0.7235	0.0102	1.0170	0.0720	–
hsa-miR-361-3p	MI0000760	UCCCCCAGGUGUGAUUCUGAUUU	0.7810	0.0129	0.8089	0.0388	–
hsa-miR-362	MI0000762	AAUCCUUGGAACCUAGGUGUGAGU	0.6802	0.0102	0.1091	0.0068	IR sensitizing
hsa-miR-362-3p	MI0000762	AACACACCUAUUCAAGGAUUCA	0.5987	0.0031	0.1801	0.0171	IR sensitizing
hsa-miR-363	MI0000764	AAUUGCACGGUAUCCAUCUGUA	1.0002	0.0246	1.7715	0.2485	–
hsa-miR-363*	MI0000764	CGGGUGGAUCACGAUGCAAUUU	0.3956	0.1047	0.0545	0.0038	Anti-proliferative
hsa-miR-365	MI0000769	UAAUGCCCCUAAAAAUCCUUAU	0.7540	0.0240	0.3213	0.0308	IR sensitizing
hsa-miR-365	MI0000767	UAAUGCCCCUAAAAAUCCUUAU	0.7982	0.0457	0.3419	0.1037	IR sensitizing
hsa-miR-367	MI0000775	AAUUGCACUUUAGCAAUGGUGA	0.7129	0.0064	1.2113	0.0143	–
hsa-miR-367*	MI0000775	ACUGUUGCUAAUAUGCAACUCU	0.6380	0.0116	0.0889	0.0126	IR sensitizing
hsa-miR-368	MI0000776	AACAUAGAGGAAAUUCCACGU	0.5459	0.0280	0.0483	0.0027	IR sensitizing
hsa-miR-369-3p	MI0000777	AAUAAUACAUGGUUGAUCUUU	0.7892	0.0383	1.0753	0.0353	–
hsa-miR-369-5p	MI0000777	AGAUCGACCGUGUUAUAUUCGC	1.1424	0.0157	4.2064	0.0948	IR protective
hsa-miR-370	MI0000778	GCCUGCUGGGGUGGAACCUGGU	0.6901	0.0086	0.7128	0.0607	–
hsa-miR-371	MI0000779	AAGUGCCGCCAUCUUUUGAGUGU	0.5925	0.0202	0.2163	0.0220	IR sensitizing
hsa-miR-371-5p	MI0000779	ACUCAAACUGUGGGGGCACU	0.9237	0.0105	2.4737	0.6890	IR protective
hsa-miR-372	MI0000780	AAAGUGCUGCGACAUUUGAGCGU	0.8707	0.0241	0.5414	0.0194	–
hsa-miR-373	MI0000781	GAAGUGCUUCGAUUUUGGGGUGU	0.7551	0.0391	0.4346	0.0055	IR sensitizing
hsa-miR-373*	MI0000781	ACUCAAAAUGGGGGCGCUUUCC	0.8890	0.0279	1.2388	0.0577	–
hsa-miR-374	MI0000782	UUAUAAUACAACCUGAUAAGUG	0.6917	0.0270	0.3513	0.0816	IR sensitizing
hsa-miR-374a*	MI0000782	CUUAUCAGAUUGUAUUGUAAUU	0.8159	0.0139	2.0573	0.2078	IR protective
hsa-miR-374b	MI0005566	AUAUAAUACAACCUGCUAAGUG	0.7118	0.0143	0.1467	0.0135	IR sensitizing
hsa-miR-374b*	MI0005566	CUUAGCAGGUUGUAUUAUCAUU	0.6648	0.0020	0.1282	0.0146	IR sensitizing
hsa-miR-375	MI0000783	UUUGUUCGUUCGGCUCGCGUGA	0.6818	0.0055	0.2955	0.0176	IR sensitizing
hsa-miR-376a	MI0000784	AUCAUAGAGGAAAAUCCACGU	0.6894	0.0300	0.1470	0.0282	IR sensitizing
hsa-miR-376a	MI0003529	AUCAUAGAGGAAAAUCCACGU	0.7697	0.0222	0.1548	0.0217	IR sensitizing
hsa-miR-376a*	MI0000784	GUAGAUUCUCCUUCUAUGAGUA	0.6199	0.0031	0.2053	0.0311	IR sensitizing
hsa-miR-376b	MI0002466	AUCAUAGAGGAAAAUCCAUGUU	0.6422	0.3699	0.5357	0.1057	–
hsa-miR-377	MI0000785	AUCACACAAAGGCAACUUUUGU	0.7637	0.0350	0.6916	0.0255	–
hsa-miR-377*	MI0000785	AGAGGUUGCCCUUGGUGAAUUC	0.4990	0.0197	0.1815	0.0261	Anti-proliferative
hsa-miR-378	MI0000786	CUCCUGACUCCAGGUCCUGUGU	0.2682	0.0122	0.0673	0.0131	Anti-proliferative
hsa-miR-379	MI0000787	UGGUAGACUAUGGAACGUAGG	0.7183	0.0142	0.2022	0.0107	IR sensitizing
hsa-miR-379*	MI0000787	UAUGUAACAUGGUCCACUAACU	0.7812	0.0178	1.1058	0.1554	–
hsa-miR-380-3p	MI0000788	UAUGUAAUAUGGUCCACAUCUU	0.7883	0.0064	0.2017	0.0162	IR sensitizing

hsa-miR-380-5p	MI0000788	UGGUUGACCAUAGAACAUGCGC	0.4649	0.0134	0.0705	0.0061	Anti-proliferative
hsa-miR-381	MI0000789	UAUACAAGGGCAAGCUCUCUGU	1.0668	0.0135	3.2926	0.4336	IR protective
hsa-miR-382	MI0000790	GAAGUUGUUCGUGGUGGAUUCG	0.8848	0.0124	0.5087	0.0527	–
hsa-miR-383	MI0000791	AGAUCAGAAGGUGAUUGUGGCU	0.9226	0.0053	0.1413	0.0182	IR sensitizing
hsa-miR-384	MI0001145	AUUCCUAGAAAUUGUUCAUA	0.8937	0.0117	1.1252	0.1474	–
hsa-miR-409-3p	MI0001735	GAAUGUUGCUCGGUGAACCCCU	0.8791	0.0288	0.7930	0.1774	–
hsa-miR-409-5p	MI0001735	AGGUUACCCGAGCAACUUUGCAU	0.8874	0.0264	1.3323	0.2365	–
hsa-miR-410	MI0002465	AAUAUAACACAGAUGGCCUGU	1.2396	0.0469	1.6677	0.1026	–
hsa-miR-411	MI0003675	UAGUAGACCGUAUAGCGUACG	0.6810	0.0156	0.1714	0.0257	IR sensitizing
hsa-miR-411*	MI0003675	UAUGUAACACGGUCCACUAACC	0.6897	0.0453	0.7192	0.0539	–
hsa-miR-412	MI0002464	ACUUCACCUGGUCCACUAGCCGU	1.1815	0.0210	3.2521	0.3003	IR protective
hsa-miR-421	MI0003685	AUCAACAGACAUUAAUUGGGCGC	0.6503	0.0206	0.1466	0.0044	IR sensitizing
hsa-miR-422a	MI0001444	ACUGGACUUAGGGUCAGAAGGC	0.7940	0.0321	0.2386	0.0391	IR sensitizing
hsa-miR-422b	MI0000786	ACUGGACUUGGAGUCAGAAGG	0.6788	0.0410	0.4399	0.0297	IR sensitizing
hsa-miR-423	MI0001445	AGCUCGGUCUGAGGCCCCUCAGU	0.6857	0.0423	0.3875	0.1104	IR sensitizing
hsa-miR-423-5p	MI0001445	UGAGGGGCAGAGAGCGAGACUUU	0.3975	0.0340	0.5479	0.0592	Anti-proliferative
hsa-miR-424	MI0001446	CAGCAGCAAUUCAUGUUUUGAA	0.2519	0.0209	0.1012	0.0055	Anti-proliferative
hsa-miR-424*	MI0001446	CAAAACGUGAGGCGCUGCUAU	0.6483	0.0584	0.2811	0.0099	IR sensitizing
hsa-miR-425	MI0001448	AUCGGGAAUGUCGUGUCCGCCC	0.8161	0.0175	0.6238	0.1147	–
hsa-miR-425-5p	MI0001448	AAUGACACGAUCACUCCCGUUGA	0.3544	0.0023	0.1502	0.0035	Anti-proliferative
hsa-miR-429	MI0001641	UAAUACUGUCUGGUAAAACCGU	0.8327	0.0057	1.9271	0.0480	–
hsa-miR-431	MI0001721	UGUCUUGCAGGCCGUCAUGCA	1.0240	0.0292	0.3906	0.0182	IR sensitizing
hsa-miR-431*	MI0001721	CAGGUCGUCUUGCAGGGCUUCU	0.7000	0.0064	0.3120	0.0525	IR sensitizing
hsa-miR-432	MI0003133	UCUUGGAGUAGGUCAUUGGGUGG	0.5607	0.3146	0.4984	0.1154	IR sensitizing
hsa-miR-432*	MI0003133	CUGGAUGGCUCCUCCAUGUCU	1.0524	0.0072	3.8939	0.0797	IR protective
hsa-miR-433	MI0001723	AUCAUGAUGGGCUCCUCGGUGU	0.9573	0.0150	1.5129	0.0896	–
hsa-miR-448	MI0001637	UUGCAUAUGUAGGAUGUCCCAU	0.9678	0.0083	0.1416	0.0112	IR sensitizing
hsa-miR-449	MI0001648	UGGCAGUGUAUUGUUAGCUGGU	0.1028	0.0304	0.0498	0.0017	Anti-proliferative
hsa-miR-449b	MI0003673	AGGCAGUGUAUUGUUAGCUGGC	0.0405	0.0070	0.0445	0.0015	Anti-proliferative
hsa-miR-450	MI0003187	UUUUGCGAUGUGUUCCUAAUAU	0.7110	0.0074	0.8752	0.1543	–
hsa-miR-450	MI0001652	UUUUGCGAUGUGUUCCUAAUAU	0.6683	0.0098	0.9284	0.1444	–
hsa-miR-450b-3p	MI0005531	UUGGGAUCAUUUUGCAUCCAUA	0.8059	0.0354	0.1829	0.0111	IR sensitizing
hsa-miR-450b-5p	MI0005531	UUUUGCAAUAUGUUCCUGAAUA	0.8114	0.0487	0.5723	0.0655	–
hsa-miR-451	MI0001729	AAACCGUUACCAUUACUGAGUU	0.9698	0.0437	1.4441	0.0927	–
hsa-miR-452	MI0001733	AACUGUUUGCAGAGGAAACUGA	0.7812	0.0112	0.8885	0.0764	–
hsa-miR-452*	MI0001733	CUCAUCUGCAAAGAAGUAAGUG	0.8246	0.0076	0.1723	0.0201	IR sensitizing
hsa-miR-453	MI0001727	AGGUUGUCCGUGGUGAGUUCGCA	0.6259	0.0588	0.1360	0.0214	IR sensitizing
hsa-miR-454-3p	MI0003820	UAGUGCAAUAUUGCUUAUAGGGU	0.8097	0.0045	0.2775	0.0278	IR sensitizing
hsa-miR-454-5p	MI0003820	ACCCUAUCAAUAUUGUCUCUGC	0.8259	0.0062	0.5963	0.0183	–
hsa-miR-455	MI0003513	UAUGUGCCUUUGGACUACAUCG	0.9443	0.0050	2.2518	0.0449	IR protective
hsa-miR-455-3p	MI0003513	GCAGUCCAUGGGCAUAUACAC	0.8003	0.0092	1.8879	0.3174	–
hsa-miR-483	MI0002467	UCACUCCUCUCCUCCCGUCUU	0.9341	0.0249	0.9446	0.2358	–
hsa-miR-483-5p	MI0002467	AAGACGGGAGGAAAGAAGGGAG	0.6595	0.0196	0.4892	0.0409	IR sensitizing
hsa-miR-484	MI0002468	UCAGGCUCAGUCCCCUCCCGAU	1.1001	0.0187	1.1478	0.1968	–
hsa-miR-485-3p	MI0002469	GUCAUACACGGCUCUCCUCUCU	1.0957	0.0099	0.7378	0.0493	–
hsa-miR-485-5p	MI0002469	AGAGGCUGGCCGUGAUGAAUUC	0.5343	0.0401	0.1239	0.0056	IR sensitizing
hsa-miR-486	MI0002470	UCCUGUACUGAGCUGCCCCGAG	1.0456	0.0204	1.5817	0.0471	–
hsa-miR-486-3p	MI0002470	CGGGGCAGCUCAGUACAGGAU	0.6170	0.0117	0.0967	0.0081	IR sensitizing
hsa-miR-487	MI0002471	AAUCAUACAGGGACAUCCAGUU	0.8891	0.0194	0.6297	0.0457	–
hsa-miR-487b	MI0003530	AAUCGUACAGGGUCAUCCACUU	0.8524	0.0117	0.2625	0.0465	IR sensitizing
hsa-miR-488	MI0003123	UUGAAAGGCUAUUUCUUGGUC	0.6810	0.0173	0.1839	0.0142	IR sensitizing
hsa-miR-488*	MI0003123	CCCAGAUAAUGGCACUCUCAA	0.5267	0.0432	0.0947	0.0084	IR sensitizing
hsa-miR-489	MI0003124	GUGACAUCACAUAUACGGCAGC	0.2339	0.0336	0.0664	0.0034	Anti-proliferative
hsa-miR-490	MI0003125	CAACCUGGAGGACUCCAUGCUG	0.8370	0.0103	0.5056	0.0296	–
hsa-miR-490-5p	MI0003125	CCAUGGAUCUCCAGGUGGGU	0.6066	0.0120	0.4238	0.0247	IR sensitizing
hsa-miR-491	MI0003126	AGUGGGGAACCCUUCCAUGAGG	0.6690	0.0282	0.1739	0.0428	IR sensitizing
hsa-miR-491-3p	MI0003126	CUUAUGCAAGAUUCCCUUCUAC	0.8107	0.0241	0.3172	0.0155	IR sensitizing
hsa-miR-492	MI0003131	AGGACCUGCGGGACAAGAUUCUU	0.8874	0.0186	0.5068	0.0741	–
hsa-miR-493	MI0003132	UUGUACAUGGUAGGCUUUCAUU	0.6948	0.0501	0.1850	0.0174	IR sensitizing
hsa-miR-493-3p	MI0003132	UGAAGGUCUACUGUGUGCCAGG	0.8973	0.0211	1.1629	0.1847	–
hsa-miR-494	MI0003134	UGAAACAUACACGGGAAACCUC	0.7433	0.0125	0.2947	0.0755	IR sensitizing
hsa-miR-495	MI0003135	AAACAAACAUGGUGCACUUCUU	0.8821	0.0174	4.2017	0.5922	IR protective
hsa-miR-496	MI0003136	UGAGUAUUACAUGGCCAAUCUC	0.6654	0.0318	0.2846	0.0470	IR sensitizing
hsa-miR-497	MI0003138	CAGCAGCACACUGUGGUUUGU	0.1585	0.0151	0.0746	0.0033	Anti-proliferative
hsa-miR-497*	MI0003138	CAAACCACACUGUGGUGUUAGA	0.7026	0.0210	0.5060	0.0582	–
hsa-miR-498	MI0003142	UUUCAAGCCAGGGGGCGUUUUUC	0.7432	0.0210	0.3820	0.0170	IR sensitizing
hsa-miR-499	MI0003183	UUAAGACUUGCAGUGAUGUUU	0.7906	0.0112	0.3561	0.0627	IR sensitizing
hsa-miR-499-3p	MI0003183	AACAUCACAGCAAGUCUGUGCU	0.4860	0.0443	0.0907	0.0072	Anti-proliferative

hsa-miR-500	MI0003184	UAAUCCUUGCUACCUGGGUGAGA	0.7435	0.0011	0.4037	0.0077	IR sensitizing
hsa-miR-500*	MI0003184	AUGCACCUGGGCAAGGAUUCUG	0.8974	0.0163	0.7622	0.0465	—
hsa-miR-501	MI0003185	AAUCCUUUGUCCCUGGGUGAGA	0.9441	0.0094	2.3920	0.0912	IR protective
hsa-miR-501-3p	MI0003185	AAUGCACCCGGGCAAGGAUUCU	0.7725	0.0093	0.4388	0.0085	IR sensitizing
hsa-miR-502	MI0003186	AUCCUUGCUAUCUGGGUGCUA	0.9502	0.0164	2.5154	0.0730	IR protective
hsa-miR-502-3p	MI0003186	AAUGCACCUGGGCAAGGAUUCA	0.6584	0.0301	0.1901	0.0122	IR sensitizing
hsa-miR-503	MI0003188	UAGCAGCGGGAACAGUUCUGCAG	0.9832	0.0119	2.4523	0.0649	IR protective
hsa-miR-504	MI0003189	AGACCCUGGUCUGCACUCUAUC	1.0856	0.0029	1.6433	0.3473	—
hsa-miR-505	MI0003190	CGUCAACACUUGCUGGUUUCCU	0.8878	0.0095	0.8676	0.0373	—
hsa-miR-505*	MI0003190	GGGAGCCAGGAAGUAUUGAUGU	0.8983	0.0079	0.2728	0.0321	IR sensitizing
hsa-miR-506	MI0003193	UAAGGCACCCUUCUGAGUAGA	0.5543	0.0333	0.0803	0.0045	IR sensitizing
hsa-miR-507	MI0003194	UUUUGCACCUUUUGGAGUGAA	0.7825	0.0055	0.3769	0.0166	IR sensitizing
hsa-miR-508	MI0003195	UGAUUGUAGCCUUUUGGAGUAGA	0.8740	0.0057	1.4288	0.1845	—
hsa-miR-508-5p	MI0003195	UACUCCAGAGGGCGUCACUCAUG	0.7548	0.0074	0.4269	0.0123	IR sensitizing
hsa-miR-509	MI0003196	UGAUUGGUACGUCUGUGGGUAG	0.6686	0.0364	0.1631	0.0229	IR sensitizing
hsa-miR-509-3-5p	MI0005717	UACUGCAGACGUGGCAAUCAUG	0.3154	0.0213	0.0711	0.0047	Anti-proliferative
hsa-miR-509-3p	MI0005530	UGAUUGGUACGUCUGUGGGUAG	0.6987	0.0088	0.0829	0.0072	IR sensitizing
hsa-miR-509-3p	MI0005717	UGAUUGGUACGUCUGUGGGUAG	0.4994	0.0241	0.0912	0.0139	Anti-proliferative
hsa-miR-509-5p	MI0003196	UACUGCAGACAGUGGCAAUCA	0.6565	0.0099	0.1109	0.0049	IR sensitizing
hsa-miR-509-5p	MI0005530	UACUGCAGACAGUGGCAAUCA	0.6132	0.0135	0.2031	0.0241	IR sensitizing
hsa-miR-510	MI0003197	UACUCAGGAGAGUGGCAAUCAC	0.3797	0.0141	0.0941	0.0279	Anti-proliferative
hsa-miR-511	MI0003128	GUGUCUUUUGCUCUGCAGUCA	0.8739	0.0117	0.5457	0.0225	—
hsa-miR-511	MI0003127	GUGUCUUUUGCUCUGCAGUCA	1.0914	0.0120	2.8442	0.1821	IR protective
hsa-miR-512-3p	MI0003140	AAGUGCUGUCAUAGCUGAGGUC	0.8856	0.0061	0.6092	0.0360	—
hsa-miR-512-3p	MI0003141	AAGUGCUGUCAUAGCUGAGGUC	0.8972	0.0110	0.6399	0.0279	—
hsa-miR-512-5p	MI0003141	CACUCAGCCUUGAGGGCACUUUC	1.0416	0.0055	3.3262	0.1569	IR protective
hsa-miR-512-5p	MI0003140	CACUCAGCCUUGAGGGCACUUUC	0.5616	0.0650	0.1510	0.0048	IR sensitizing
hsa-miR-513	MI0003191	UUCACAGGGAGGUGUCAU	0.8605	0.0186	0.5410	0.1058	—
hsa-miR-513	MI0003192	UUCACAGGGAGGUGUCAU	0.7976	0.0375	0.8446	0.1911	—
hsa-miR-513a-3p	MI0003192	UAAAUUUCACCUUUCUGAGAAGG	0.5170	0.0117	0.0816	0.0023	IR sensitizing
hsa-miR-513a-3p	MI0003191	UAAAUUUCACCUUUCUGAGAAGG	0.6303	0.0384	0.1115	0.0187	IR sensitizing
hsa-miR-513b	MI0006648	UUCACAAGGAGGUGUCAUUUAU	0.9137	0.0137	1.7566	0.0194	—
hsa-miR-513c	MI0006649	UUCUCAAGGAGGUGUCGUUUAU	1.0313	0.0723	0.4095	0.0132	IR sensitizing
hsa-miR-514	MI0003199	AUUGACACUUCUGUGAGUAGA	0.7565	0.0036	1.6949	0.2566	—
hsa-miR-514	MI0003200	AUUGACACUUCUGUGAGUAGA	0.7103	0.0119	2.4265	0.3499	IR protective
hsa-miR-514	MI0003198	AUUGACACUUCUGUGAGUAGA	0.8253	0.0087	3.7086	0.5119	IR protective
hsa-miR-515-3p	MI0003147	GAGUGCCUUCUUUUGGAGCGUU	0.7779	0.0138	0.5092	0.0793	—
hsa-miR-515-3p	MI0003144	GAGUGCCUUCUUUUGGAGCGUU	0.7533	0.0073	0.6548	0.1033	—
hsa-miR-515-5p	MI0003147	UUCUCCAAAAGAAAGCACUUUCUG	0.5560	0.3078	0.9750	0.0343	—
hsa-miR-515-5p	MI0003144	UUCUCCAAAAGAAAGCACUUUCUG	0.8860	0.0181	0.4687	0.1195	IR sensitizing
hsa-miR-516-3p	MI0003180	UGCUUCCUUUCAGAGGGU	0.8932	0.0036	0.6242	0.0528	—
hsa-miR-516-3p	MI0003172	UGCUUCCUUUCAGAGGGU	0.5669	0.3135	2.5551	0.1291	IR protective
hsa-miR-516-3p	MI0003167	UGCUUCCUUUCAGAGGGU	0.8529	0.0210	0.2033	0.0126	IR sensitizing
hsa-miR-516-3p	MI0003181	UGCUUCCUUUCAGAGGGU	0.6805	0.0713	0.2239	0.0069	IR sensitizing
hsa-miR-516-5p	MI0003172	AUCUGGAGGUAAGAAGCACUUU	0.2144	0.0145	0.1426	0.0253	Anti-proliferative
hsa-miR-516-5p	MI0003167	AUCUGGAGGUAAGAAGCACUUU	0.3409	0.0538	0.1123	0.0218	Anti-proliferative
hsa-miR-516a-5p	MI0003180	UUCUCGAGGAAAGAAGCACUUUC	0.7827	0.0085	0.8819	0.2022	—
hsa-miR-516a-5p	MI0003181	UUCUCGAGGAAAGAAGCACUUUC	0.7006	0.0074	0.3516	0.0708	IR sensitizing
hsa-miR-517*	MI0003165	CCUCUAGAUGGAAGCACUGUCU	0.9449	0.0156	0.7267	0.1038	—
hsa-miR-517*	MI0003174	CCUCUAGAUGGAAGCACUGUCU	0.9309	0.0248	0.8625	0.0561	—
hsa-miR-517*	MI0003161	CCUCUAGAUGGAAGCACUGUCU	0.9436	0.0104	1.0990	0.0562	—
hsa-miR-517a	MI0003161	AUCGUGCAUCCCUUUAGAGUGU	0.7709	0.0488	0.3399	0.0559	IR sensitizing
hsa-miR-517b	MI0003165	UCGUGCAUCCCUUUAGAGUGUU	0.9737	0.0309	0.1876	0.0104	IR sensitizing
hsa-miR-517c	MI0003174	AUCGUGCAUCCUUUUAGAGUGU	0.6778	0.0619	0.1651	0.0137	IR sensitizing
hsa-miR-518a	MI0003173	GAAAGCGCUUCCCUUUGCUGGA	0.5982	0.0336	0.2504	0.0507	IR sensitizing
hsa-miR-518a	MI0003170	GAAAGCGCUUCCCUUUGCUGGA	0.6013	0.0220	0.4107	0.0840	IR sensitizing
hsa-miR-518a-5p	MI0003170	CUGCAAAGGGAAGCCCUUUC	0.1745	0.0173	0.1342	0.0146	Anti-proliferative
hsa-miR-518a-5p	MI0003173	CUGCAAAGGGAAGCCCUUUC	0.2110	0.0103	0.1465	0.0210	Anti-proliferative
hsa-miR-518b	MI0003156	CAAAGCGCUCCCCUUUAGAGGU	0.5793	0.0042	0.1397	0.0149	IR sensitizing
hsa-miR-518c	MI0003159	CAAAGCGCUUCUCUUUAGAGUGU	0.6253	0.0183	0.4060	0.0622	IR sensitizing
hsa-miR-518c*	MI0003159	UCUCUGGAGGGAAGCACUUUCUG	0.9459	0.0129	0.3840	0.0260	IR sensitizing
hsa-miR-518d	MI0003171	CAAAGCGCUUCCCUUUGGAGC	0.5266	0.0046	0.1583	0.0073	IR sensitizing
hsa-miR-518d-5p	MI0003171	CUCUAGAGGGAAGCACUUUCUG	0.8675	0.0075	1.3506	0.2692	—
hsa-miR-518e	MI0003169	AAAGCGCUUCCCUUCAGAGUG	0.7421	0.0067	0.7594	0.1167	—
hsa-miR-518e*	MI0003169	CUCUAGAGGGAAGCGCUUUCUG	0.8026	0.0229	0.9902	0.2155	—
hsa-miR-518f	MI0003154	GAAAGCGCUUCUCUUUAGAGG	0.7276	0.0270	1.2259	0.1963	—
hsa-miR-518f*	MI0003154	CUCUAGAGGGAAGCACUUUCUC	0.8940	0.0024	1.2722	0.1915	—
hsa-miR-519a	MI0003178	AAAGUGCAUCCUUUUAGAGUGU	0.8310	0.0043	0.8045	0.1187	—

hsa-miR-519a	MI0003182	AAAGUGCAUCCUUUUAGAGUGU	0.9624	0.0040	1.2077	0.1815	–
hsa-miR-519a*	MI0003178	CUCUAGAGGGAAGCGCUUUCUG	0.7510	0.0092	0.7342	0.0275	–
hsa-miR-519b	MI0003151	AAAGUGCAUCCUUUUAGAGGUU	0.9189	0.0113	0.7252	0.1405	–
hsa-miR-519b-5p	MI0003151	CUCUAGAGGGAAGCGCUUUCUG	0.7920	0.0042	0.9310	0.0826	–
hsa-miR-519c	MI0003148	AAAGUGCAUCUUUUUAGAGGAU	0.9693	0.0130	0.5905	0.0473	–
hsa-miR-519d	MI0003162	CAAAGUGCCUCCCUUUAGAGUG	0.8220	0.0202	2.0405	0.3401	IR protective
hsa-miR-519e	MI0003145	AAGUGCCUCCUUUUAGAGUGUU	0.6499	0.0062	0.3304	0.0517	IR sensitizing
hsa-miR-519e*	MI0003145	UUCUCCAAAAGGGAGCACUUUC	0.8457	0.0089	0.3640	0.0469	IR sensitizing
hsa-miR-520a	MI0003149	AAAGUGCUUCCCUUUGGACUGU	0.8745	0.0012	0.7234	0.0544	–
hsa-miR-520a*	MI0003149	CUCCAGAGGGAAGUACUUUCU	0.7696	0.0110	0.4450	0.0159	IR sensitizing
hsa-miR-520b	MI0003155	AAAGUGCUUCCUUUUAGAGGG	0.8910	0.0112	0.8880	0.0533	–
hsa-miR-520c	MI0003158	AAAGUGCUUCCUUUUAGAGGGU	0.7876	0.0048	0.9885	0.1667	–
hsa-miR-520c-5p	MI0003158	CUCUAGAGGGAAGCACUUUCUG	0.8440	0.0019	1.4779	0.0571	–
hsa-miR-520d	MI0003164	AAAGUGCUUCUCUUUGGUGGGU	0.9032	0.0444	1.2926	0.1928	–
hsa-miR-520d*	MI0003164	CUACAAAGGGAAGCCCUUUC	0.7608	0.0240	0.1496	0.0244	IR sensitizing
hsa-miR-520e	MI0003143	AAAGUGCUUCCUUUUUGAGGG	0.9157	0.0439	0.6997	0.0412	–
hsa-miR-520f	MI0003146	AAGUGCUUCCUUUUAGAGGGUU	0.8903	0.0068	0.6164	0.0233	–
hsa-miR-520g	MI0003166	ACAAAGUGCUUCCCUUUAGAGUGU	0.9687	0.0017	1.3169	0.0865	–
hsa-miR-520h	MI0003175	ACAAAGUGCUUCCCUUUAGAGU	0.9646	0.0093	1.6814	0.0516	–
hsa-miR-521	MI0003163	AACGCACUUCCCUUUAGAGUGU	0.7223	0.0267	0.2880	0.0129	IR sensitizing
hsa-miR-521	MI0003176	AACGCACUUCCCUUUAGAGUGU	0.7187	0.0149	0.2977	0.0275	IR sensitizing
hsa-miR-522	MI0003177	AAAAUGGUUCCCUUUAGAGUGU	0.0629	0.0050	0.0537	0.0088	Anti-proliferative
hsa-miR-522*	MI0003177	CUCUAGAGGGAAGCGCUUUCUG	0.7823	0.0121	0.8822	0.0178	–
hsa-miR-523	MI0003153	GAACGCGCUUCCCUAUAGAGGGU	0.9401	0.0230	3.4475	0.4857	IR protective
hsa-miR-523*	MI0003153	CUCUAGAGGGAAGCGCUUUCUG	0.7568	0.0188	1.2392	0.0438	–
hsa-miR-524	MI0003160	GAAGGCGCUUCCCUUUGGAGU	0.8323	0.0173	0.4250	0.0094	IR sensitizing
hsa-miR-524*	MI0003160	CUACAAAGGGAAGCACUUUCUC	0.4239	0.2354	0.0898	0.0023	Anti-proliferative
hsa-miR-525	MI0003152	CUCCAGAGGGAUGCACUUUCU	0.6866	0.0198	0.2732	0.0258	IR sensitizing
hsa-miR-525*	MI0003152	GAAGGCGCUUCCCUUUAGAGCG	0.8352	0.0156	0.7248	0.1128	–
hsa-miR-526a	MI0003157	CUCUAGAGGGAAGCACUUUCUG	0.8959	0.0102	1.8187	0.2582	–
hsa-miR-526a	MI0003168	CUCUAGAGGGAAGCACUUUCUG	0.8567	0.0041	2.2646	0.3387	IR protective
hsa-miR-526b	MI0003150	CUCUUGAGGGAAGCACUUUCUGU	0.6406	0.0145	0.1201	0.0204	IR sensitizing
hsa-miR-526b*	MI0003150	GAAAGUGCUUCCUUUUAGAGGC	0.8523	0.0155	1.8542	0.2576	–
hsa-miR-526c	MI0003148	CUCUAGAGGGAAGCGCUUUCUG	0.8395	0.0103	1.0275	0.1531	–
hsa-miR-527	MI0003179	CUGCAAAGGGAAGCCCUUUC	0.3343	0.0352	0.1093	0.0217	Anti-proliferative
hsa-miR-532	MI0003205	CAUGCCUUGAGUGUAGGACCGU	0.6707	0.0056	0.0927	0.0076	IR sensitizing
hsa-miR-532-3p	MI0003205	CCUCCCACACCCAAGGCUUGCA	0.7441	0.0181	0.7944	0.0212	–
hsa-miR-539	MI0003514	GGAGAAAUUAUCCUUGGUGUGU	0.9824	0.0172	1.5754	0.2226	–
hsa-miR-541	MI0005539	UGGUGGGCACAGAAUCUGGACU	0.1194	0.0365	0.0337	0.0025	Anti-proliferative
hsa-miR-541*	MI0005539	AAAGGAUUCUGCUGUCGGUCCCACU	0.8635	0.0169	0.7416	0.0837	–
hsa-miR-542-3p	MI0003686	UGUGACAGAUUGAUAACUGAAA	0.7193	0.0082	0.1917	0.0233	IR sensitizing
hsa-miR-542-5p	MI0003686	UCGGGGAUCAUCAUGUCACGAGA	0.7996	0.0166	0.7941	0.1062	–
hsa-miR-543	MI0005565	AAACAUUCGCGGUGCACUUCUU	0.8208	0.0275	0.7029	0.0050	–
hsa-miR-544	MI0003515	AUUCUGCAUUUUUAGCAAGUUC	0.3391	0.0519	0.0860	0.0209	Anti-proliferative
hsa-miR-545	MI0003516	UCAGCAAACAUUUAUUGUGUGC	0.8838	0.0154	1.5556	0.0835	–
hsa-miR-545*	MI0003516	UCAGUAAAUGUUUAUUAGAUGA	0.8398	0.0070	1.7916	0.1651	–
hsa-miR-548a	MI0003612	CAAAACUGGCAAUUACUUUUGC	0.5615	0.0246	0.1504	0.0409	IR sensitizing
hsa-miR-548a	MI0003598	CAAAACUGGCAAUUACUUUUGC	0.7331	0.0052	0.2362	0.0290	IR sensitizing
hsa-miR-548a	MI0003593	CAAAACUGGCAAUUACUUUUGC	0.9207	0.0168	0.2621	0.0571	IR sensitizing
hsa-miR-548a-5p	MI0003612	AAAAGUAAUUGCGAGUUUUACC	0.8131	0.0236	3.0684	0.3806	IR protective
hsa-miR-548b	MI0003596	CAAGAACCUCAGUUGCUUUUGU	0.8633	0.0147	2.5990	0.2763	IR protective
hsa-miR-548b-5p	MI0003596	AAAAGUAAUUGUGGUUUUGGCC	0.7502	0.0191	1.3305	0.2143	–
hsa-miR-548c	MI0003630	CAAAAAUCUCAAUUACUUUUGC	0.7667	0.0076	1.9333	0.2176	–
hsa-miR-548c-5p	MI0003630	AAAAGUAAUUGCGGUUUUUGCC	0.7914	0.0063	2.9747	0.6421	IR protective
hsa-miR-548d	MI0003668	CAAAAACCACAGUUUCUUUUGC	0.8520	0.0036	0.5751	0.0556	–
hsa-miR-548d	MI0003671	CAAAAACCACAGUUUCUUUUGC	0.7955	0.0054	0.9400	0.0139	–
hsa-miR-548d-5p	MI0003671	AAAAGUAAUUGUGGUUUUUGCC	0.9354	0.0061	4.9504	0.7523	IR protective
hsa-miR-548d-5p	MI0003668	AAAAGUAAUUGUGGUUUUUGCC	0.9215	0.0113	5.3036	0.3361	IR protective
hsa-miR-549	MI0003679	UGACAACUAUGGAUGAGCUCU	0.8640	0.0312	1.2823	0.0319	–
hsa-miR-550	MI0003600	AGUGCCUGAGGGAGUAAGAGCCC	0.6743	0.0180	0.6896	0.0635	–
hsa-miR-550	MI0003601	AGUGCCUGAGGGAGUAAGAGCCC	0.7218	0.0446	0.8664	0.0597	–
hsa-miR-550	MI0003601	UGUCUUACUCCCUCAGGCACAU	0.6330	0.0177	0.3177	0.0879	IR sensitizing
hsa-miR-550	MI0003600	UGUCUUACUCCCUCAGGCACAU	0.6664	0.0163	0.3968	0.0551	IR sensitizing
hsa-miR-551a	MI0003556	GCGACCCACUCUUGGUUUCCA	0.5545	0.3069	1.2156	0.0853	–
hsa-miR-551b	MI0003575	GCGACCCAUACUUGGUUUCAG	1.1278	0.0089	4.0334	0.1548	IR protective
hsa-miR-551b*	MI0003575	GAAAUCAAGCGUGGGUGAGACC	0.8042	0.0185	1.9005	0.1411	–
hsa-miR-552	MI0003557	AACAGGUGACUGGUUAGACAA	0.4642	0.0147	0.0714	0.0055	Anti-proliferative
hsa-miR-553	MI0003558	AAAACGGUGAGAUUUUGUUUU	0.8194	0.0175	1.5763	0.0577	–

hsa-miR-554	MI0003559	GCUAGUCCUGACUCAGCCAGU	0.9186	0.0031	1.2594	0.0934	–
hsa-miR-555	MI0003561	AGGGUAAGCUGAACCUCUGAU	0.1162	0.0134	0.0879	0.0076	Anti-proliferative
hsa-miR-556	MI0003562	GAUGAGCUCAUUGUAAUAUGAG	0.8197	0.0145	1.7974	0.3142	–
hsa-miR-556-3p	MI0003562	AUAUUACCAUUAGCUCAUCUUU	0.7398	0.0212	0.3305	0.0264	IR sensitizing
hsa-miR-557	MI0003563	GUUUGCACGGGUGGGCCUUGUCU	0.7399	0.0086	0.4110	0.0303	IR sensitizing
hsa-miR-558	MI0003564	UGAGCUGCUGUACCAAAAU	0.6841	0.0507	0.1866	0.0166	IR sensitizing
hsa-miR-559	MI0003565	UAAAGUAAAUAUGCACCAAAA	0.9235	0.0211	1.9453	0.2155	–
hsa-miR-561	MI0003567	CAAAGUUUAAGAUCCUUGAAGU	0.8133	0.0008	0.2874	0.1278	IR sensitizing
hsa-miR-562	MI0003568	AAAGUAGCUGUACCAUUUGC	0.6971	0.0215	0.1619	0.0192	IR sensitizing
hsa-miR-563	MI0003569	AGGUUGACAUACGUUUCCC	0.3863	0.2158	0.0908	0.0027	Anti-proliferative
hsa-miR-564	MI0003570	AGGCACGGUGUCAGCAGGC	0.8192	0.0116	0.2706	0.0227	IR sensitizing
hsa-miR-566	MI0003572	GGGCGCCUGUGAUCCCAAC	1.0821	0.0056	2.8244	0.2211	IR protective
hsa-miR-567	MI0003573	AGUAUGUUCUUCCAGGACAGAAC	0.8262	0.0014	0.3297	0.0680	IR sensitizing
hsa-miR-568	MI0003574	AUGUAUAAAUGUAUACACAC	0.9697	0.0101	3.1237	0.2010	IR protective
hsa-miR-569	MI0003576	AGUUAAUGAAUCCUGGAAAGU	1.0259	0.0196	1.8841	0.3260	–
hsa-miR-570	MI0003577	CGAAAACAGCAAUUACCUUUGC	0.8139	0.0214	0.3634	0.0646	IR sensitizing
hsa-miR-571	MI0003578	UGAGUUGGCCAUCUGAGUGAG	0.5747	0.0629	0.1215	0.0034	IR sensitizing
hsa-miR-572	MI0003579	GUCCGCUCGGCGGUGGCCCA	0.9886	0.0078	1.9423	0.1689	–
hsa-miR-573	MI0003580	CUGAAGUGAUGUGUAACUGAUCAG	0.9939	0.0322	0.7947	0.0439	–
hsa-miR-574	MI0003581	CACGCUCAUGCACACACCCACA	0.8017	0.0360	0.6905	0.1058	–
hsa-miR-574-5p	MI0003581	UGAGUGUGUGUGUGUGAGUGUGU	0.6811	0.0222	0.4920	0.0911	IR sensitizing
hsa-miR-575	MI0003582	GAGCCAGUUGGACAGGAGC	0.6206	0.3550	0.6197	0.0790	–
hsa-miR-576	MI0003583	AUUCUAAUUUCUCCACGUCUUU	0.7617	0.0037	1.4493	0.2346	–
hsa-miR-576-3p	MI0003583	AAGAUGUGGAAAAAUUGGAAUC	0.2807	0.0166	0.1449	0.0236	Anti-proliferative
hsa-miR-577	MI0003584	UAGAUAAAAUAUUGGUACCUG	1.0014	0.0250	4.8169	0.7454	IR protective
hsa-miR-578	MI0003585	CUUCUUGUGCUCUAGGAUUGU	0.8284	0.0423	0.0951	0.0195	IR sensitizing
hsa-miR-579	MI0003586	UUCAUUUGGUAUAAACCGCGAUU	0.7297	0.0094	0.1653	0.0401	IR sensitizing
hsa-miR-580	MI0003587	UUGAGAAUGAUGAAUCAUUAGG	0.8543	0.0113	1.0707	0.1375	–
hsa-miR-581	MI0003588	UCUUGUGUUCUCUAGAUCAGU	0.8913	0.0332	1.2881	0.3055	–
hsa-miR-582	MI0003589	UUACAGUUGUUCAACCAGUUACU	0.8277	0.0316	1.1894	0.1452	–
hsa-miR-582-3p	MI0003589	UAACUGGUUGAACAACUGAACC	0.6629	0.0258	0.2721	0.0631	IR sensitizing
hsa-miR-583	MI0003590	CAAAGAGGAAGGUCCCAUUAC	0.8020	0.0215	0.3255	0.0267	IR sensitizing
hsa-miR-584	MI0003591	UUAUGGUUUGCCUGGGACUGAG	0.9960	0.0202	0.6305	0.1587	–
hsa-miR-585	MI0003592	UGGGCGUAUCUGUAUGCUA	0.6730	0.0421	0.0649	0.0256	IR sensitizing
hsa-miR-586	MI0003594	UAUGCAUUGUAUUUUUAGGUCC	0.8685	0.0187	1.2299	0.1391	–
hsa-miR-587	MI0003595	UUUCCAUAGGUGAUGAGUCAC	0.8276	0.0284	2.6334	0.1851	IR protective
hsa-miR-588	MI0003597	UUGGCCACAAUGGGUUAGAAC	0.5124	0.0164	0.2784	0.0566	IR sensitizing
hsa-miR-589	MI0003599	UGAGAACCACGUCUGCUCUGAG	0.6504	0.0510	0.4088	0.0425	IR sensitizing
hsa-miR-589*	MI0003599	UCAGAACAAAUGCCGGUUCCCAGA	0.9437	0.0105	1.2291	0.1367	–
hsa-miR-590	MI0003602	GAGCUUAUUCAUAAAAGUGCAG	0.7327	0.0198	1.0797	0.0457	–
hsa-miR-590-3p	MI0003602	UAAUUUUAUGUAUAAGCUAGU	0.7241	0.0287	0.8250	0.0788	–
hsa-miR-591	MI0003603	AGACCAUGGGUUCUCAUUGU	0.6342	0.0056	0.3093	0.0671	IR sensitizing
hsa-miR-592	MI0003604	UUGUGUCAAUAUGCGAUGAUGU	0.8591	0.0113	0.9833	0.2716	–
hsa-miR-593	MI0003605	UGUCUCUGCUGGGGUUUCU	0.5897	0.0218	0.1131	0.0270	IR sensitizing
hsa-miR-593*	MI0003605	AGGCACCAGCCAGGCAUUGCUCAGC	0.6574	0.0165	0.2943	0.0542	IR sensitizing
hsa-miR-595	MI0003607	GAAGUGUGCCGUGGUGUGUCU	0.8089	0.0082	0.4947	0.0571	IR sensitizing
hsa-miR-596	MI0003608	AAGCCUGCCCGGCUCCUCGGG	0.6315	0.0156	0.2701	0.0936	IR sensitizing
hsa-miR-597	MI0003609	UGUGUCACUCGAUGACCACUGU	0.6543	0.0206	1.2019	0.1515	–
hsa-miR-598	MI0003610	UACGUCAUCGUUGUCAUCGUCA	0.8818	0.0156	2.0214	0.4670	IR protective
hsa-miR-599	MI0003611	GUUGUGUCAGUUUAUCAAAC	0.8236	0.0071	0.8765	0.0618	–
hsa-miR-600	MI0003613	ACUUACAGACAAGAGCCUUGCUC	0.7186	0.0455	0.2020	0.0357	IR sensitizing
hsa-miR-601	MI0003614	UGGUCUAGGAUUGUUGGAGGAG	0.6073	0.0075	0.2373	0.0500	IR sensitizing
hsa-miR-602	MI0003615	GACACGGGCGACAGCUGCGGCCC	0.7976	0.0082	1.2706	0.0643	–
hsa-miR-603	MI0003616	CACACACUGCAAUUACUUUUGC	0.6861	0.0068	0.3129	0.0287	IR sensitizing
hsa-miR-604	MI0003617	AGGCUGCGGAAUUCAGGAC	0.1753	0.0127	0.1343	0.0359	Anti-proliferative
hsa-miR-605	MI0003618	UAAAUCCCAUGGUGCCUUCUCCU	0.7534	0.0176	0.3836	0.0676	IR sensitizing
hsa-miR-606	MI0003619	AAACUACUGAAAAUCAAAGAU	0.7274	0.0068	1.0708	0.2286	–
hsa-miR-607	MI0003620	GUUCAAAUCCAGAUCUAUAAC	0.7585	0.0071	0.5614	0.0617	–
hsa-miR-608	MI0003621	AGGGGUGGUGUUGGGACAGCUCCGU	0.2620	0.0099	0.0577	0.0085	Anti-proliferative
hsa-miR-609	MI0003622	AGGGUGUUUCUCUCAUCUCU	0.5675	0.0068	0.1130	0.0267	IR sensitizing
hsa-miR-610	MI0003623	UGAGCUAAAUGUGUGCUGGGA	0.9146	0.0125	1.3326	0.0994	–
hsa-miR-611	MI0003624	GCGAGGACCCCUCGGGGUCUGAC	0.8113	0.0244	3.1278	0.4857	IR protective
hsa-miR-612	MI0003625	GCUGGGCAGGGCUUCUGAGCUCCUU	0.7708	0.0091	0.2953	0.0192	IR sensitizing
hsa-miR-613	MI0003626	AGGAAUGUUCCUUCUUUGCC	0.4087	0.0740	0.1433	0.0242	Anti-proliferative
hsa-miR-614	MI0003627	GAACGCCUGUUCUUGCCAGGUGG	0.6889	0.0176	0.8320	0.0181	–
hsa-miR-615	MI0003628	UCCGAGCCUGGGUCUCCCUCUU	0.7846	0.0025	1.2496	0.1866	–
hsa-miR-615-5p	MI0003628	GGGGGUCCCCGGUGCUCGGAUC	0.7826	0.0126	3.2537	0.5938	IR protective
hsa-miR-616	MI0003629	AGUCAUUGGAGGGUUUGAGCAG	0.7105	0.0154	0.8165	0.0632	–

hsa-miR-616*	MI0003629	ACUCAAAACCCUUCAGUGACUU	0.7941	0.0126	1.9116	0.1611	—
hsa-miR-617	MI0003631	AGACUUCCCAUUUGAAGGUGGC	0.6074	0.0163	0.1731	0.0135	IR sensitizing
hsa-miR-618	MI0003632	AAACUCUACUUGUCCUUCUGAGU	0.6362	0.0250	0.4019	0.1124	IR sensitizing
hsa-miR-619	MI0003633	GACCUGGACAUGUUUGUGCCCAGU	0.6414	0.0214	0.3375	0.0715	IR sensitizing
hsa-miR-620	MI0003634	AUGGAGAUAGAUAUAGAAAU	0.7084	0.0167	0.5397	0.0243	—
hsa-miR-621	MI0003635	GGCUAGCAACAGCGCUUACCU	0.7510	0.0155	0.2068	0.0320	IR sensitizing
hsa-miR-622	MI0003636	ACAGUCUGCUGAGGUUGGAGC	0.9046	0.0134	2.7038	0.6465	IR protective
hsa-miR-623	MI0003637	AUCCCUUGCAGGGGCUGUUGGGU	0.8219	0.0397	2.2062	0.0574	IR protective
hsa-miR-624	MI0003638	CACAAGGUAUUGGUAUUACCU	0.6239	0.0114	0.4461	0.0622	IR sensitizing
hsa-miR-624*	MI0003638	UAGUACCAGUACCUUGUGUUCA	0.9655	0.0188	0.2953	0.0430	IR sensitizing
hsa-miR-625	MI0003639	AGGGGGAAAGUUCUAUAGUCC	0.2739	0.0345	0.1033	0.0304	Anti-proliferative
hsa-miR-625*	MI0003639	GACUAUAGAACUUUCCCCCUCA	0.7116	0.0143	0.2208	0.0136	IR sensitizing
hsa-miR-626	MI0003640	AGCUGUCUGAAAAUGUCUU	0.8906	0.0107	1.8490	0.0199	—
hsa-miR-627	MI0003641	GUGAGUCUCUAAGAAAAGAGGA	0.8221	0.0418	1.6907	0.1858	—
hsa-miR-628	MI0003642	UCUAGUAAGAGUGGCAGUCGA	0.6996	0.0051	0.9057	0.1626	—
hsa-miR-628-5p	MI0003642	AUGCUGACAUAUUUACUAGAGG	0.7459	0.0345	0.1549	0.0100	IR sensitizing
hsa-miR-629	MI0003643	UGGGUUUACGUUGGGAGAACU	0.5570	0.0196	0.2582	0.0310	IR sensitizing
hsa-miR-629*	MI0003643	GUUCUCCCAACGUAAGCCCAGC	0.6863	0.0072	0.0951	0.0062	IR sensitizing
hsa-miR-630	MI0003644	AGUAUUCUGUACCAGGGAAGGU	0.7773	0.0159	0.2863	0.0662	IR sensitizing
hsa-miR-631	MI0003645	AGACCUGGCCCAGACCUCAGC	0.1615	0.0060	0.0613	0.0077	Anti-proliferative
hsa-miR-632	MI0003647	GUGUCUGCUUCCUGUGGGA	0.9399	0.0300	1.2700	0.1292	—
hsa-miR-633	MI0003648	CUAAUAGUAUCUACCACAAUAAA	0.8992	0.0149	0.7176	0.0351	—
hsa-miR-634	MI0003649	AACCAGCACCCCAACUUUGGAC	0.6078	0.0142	0.1465	0.0325	IR sensitizing
hsa-miR-635	MI0003650	ACUUGGGCACUGAAACAAUGUCC	0.8528	0.0108	0.2309	0.0293	IR sensitizing
hsa-miR-636	MI0003651	UGUGCUUGCUCGUCCCGCCCGCA	0.7225	0.0062	1.7883	0.3065	—
hsa-miR-637	MI0003652	ACUGGGGGCUUUCGGGCUCUGCGU	0.2753	0.0997	0.0879	0.0162	Anti-proliferative
hsa-miR-638	MI0003653	AGGGAUCGCGGGCGGGUGGCGGCCU	0.8444	0.0269	2.1147	0.0481	IR protective
hsa-miR-639	MI0003654	AUCGCUGCGGUUGCGAGCGCUGU	0.6498	0.0083	1.5079	0.0576	—
hsa-miR-640	MI0003655	AUGAUCCAGGAACCUGCCUCU	0.6463	0.0554	0.3225	0.0444	IR sensitizing
hsa-miR-641	MI0003656	AAAGACAUAGGAUAGAGUCACCUC	0.9653	0.0093	2.2577	0.0540	IR protective
hsa-miR-642	MI0003657	GUCCCUCUCCAAAUGUGUCUUG	0.9330	0.0099	2.2167	0.0338	IR protective
hsa-miR-643	MI0003658	ACUUGUAUGCUAGCUCAGGUAG	0.8890	0.0140	1.9603	0.0840	—
hsa-miR-644	MI0003659	AGUGUGGCUUUCUUAGAGC	0.0608	0.0090	0.0480	0.0083	Anti-proliferative
hsa-miR-645	MI0003660	UCUAGGCUGGUACUGCUGA	0.6605	0.0112	0.5028	0.0292	—
hsa-miR-646	MI0003661	AAGCAGCUGCCUCUGAGGC	0.1223	0.0295	0.0642	0.0030	Anti-proliferative
hsa-miR-647	MI0003662	GUGGCUGCACUCACUUCCUUC	0.0688	0.0023	0.0996	0.0107	Anti-proliferative
hsa-miR-648	MI0003663	AAGUGUGCAGGGCACUGGU	0.7487	0.0033	0.1664	0.0020	IR sensitizing
hsa-miR-649	MI0003664	AAACCUGUGUUGUUCAAGAGUC	0.7175	0.0030	1.1632	0.0345	—
hsa-miR-650	MI0003665	AGGAGGCAGCGCUCUCAGGAC	0.2105	0.0215	0.1011	0.0059	Anti-proliferative
hsa-miR-651	MI0003666	UUUAGGAUAAGCUUGACUUUUG	0.7502	0.0072	0.9620	0.0514	—
hsa-miR-652	MI0003667	AAUGGCGCCACUAGGGUUGUG	0.7709	0.0078	0.6366	0.1118	—
hsa-miR-653	MI0003674	GUGUUGAAACAAUCUCUACUG	0.7949	0.0019	1.4973	0.2517	—
hsa-miR-654	MI0003676	UGGUGGGCCGCAGAACAUGUGC	0.1101	0.0041	0.0489	0.0030	Anti-proliferative
hsa-miR-654-3p	MI0003676	UAUGUCUGCUGACCAUCACCUU	0.8659	0.0122	1.2998	0.1577	—
hsa-miR-655	MI0003677	AUAAUACAUGGUUAACCUCUUU	0.2905	0.0420	0.0589	0.0022	Anti-proliferative
hsa-miR-656	MI0003678	AAUAUUAUACAGUCAACCUCU	0.6830	0.0023	0.2882	0.0292	IR sensitizing
hsa-miR-657	MI0003681	GGCAGGUUCUCACCCUCUCUAGG	0.4618	0.0162	0.1648	0.0010	Anti-proliferative
hsa-miR-658	MI0003682	GGCGGAGGGAAGUAGGUCCGUUGGU	0.8937	0.0230	1.8790	0.1013	—
hsa-miR-659	MI0003683	CUUGGUUCAGGGAGGGUCCCCA	0.6451	0.0122	0.6915	0.0781	—
hsa-miR-660	MI0003684	UACCCAUUGCAUAUCGGAGUUG	0.8994	0.0159	0.2500	0.0016	IR sensitizing
hsa-miR-661	MI0003669	UGCCUGGGUCUCUGGCCUGCGCGU	0.5180	0.0122	0.1486	0.0055	IR sensitizing
hsa-miR-662	MI0003670	UCCCACGUUGUGGCCCAGCAG	0.8326	0.0290	1.6693	0.0469	—
hsa-miR-663	MI0003672	AGGCGGGGCGCCGCGGGACCGC	0.6184	0.0008	0.2509	0.0108	IR sensitizing
hsa-miR-665	MI0005563	ACCAGGAGGCUGAGGCCCCU	0.2821	0.0050	0.1051	0.0027	Anti-proliferative
hsa-miR-668	MI0003761	UGUCACUCGGCUCGGCCCACUAC	0.8461	0.0164	0.7148	0.0135	—
hsa-miR-671	MI0003760	AGGAAGCCCUGGAGGGGCUGGAG	0.7896	0.0102	0.4862	0.0196	IR sensitizing
hsa-miR-671-3p	MI0003760	UCCGGUUCUCAGGGCUCCACC	0.1364	0.0115	0.0474	0.0021	Anti-proliferative
hsa-miR-675	MI0005416	UGGUGCGGAGAGGGCCCACAGUG	0.6181	0.0192	0.5753	0.1506	—
hsa-miR-7	MI0000264	UGGAAGACUAGUGAUUUUGUUGU	0.1714	0.0120	0.0911	0.0155	Anti-proliferative
hsa-miR-7	MI0000265	UGGAAGACUAGUGAUUUUGUUGU	0.2570	0.0275	0.0978	0.0075	Anti-proliferative
hsa-miR-7	MI0000263	UGGAAGACUAGUGAUUUUGUUGU	0.2629	0.0385	0.1305	0.0262	Anti-proliferative
hsa-miR-708	MI0005543	AAGGAGCUUACAAUCUAGCUGGG	0.3871	0.0055	0.0467	0.0063	Anti-proliferative
hsa-miR-708*	MI0005543	CAACUAGACUGUGAGCUUCUAG	0.9674	0.0174	0.1646	0.0068	IR sensitizing
hsa-miR-7-1*	MI0000263	CAACAAAUCACAGUCUGCCAUA	0.9904	0.0335	1.6279	0.0521	—
hsa-miR-7-2*	MI0000264	CAACAAAUCCCAGUCUACCUAA	0.8262	0.0202	2.3763	0.4132	IR protective
hsa-miR-744	MI0005559	UGCGGGGCUAGGGCUAACAGCA	0.1974	0.0020	0.0251	0.0023	Anti-proliferative
hsa-miR-744*	MI0005559	CUGUUGCCACUAACCUCAACCU	0.5920	0.0078	0.0592	0.0072	IR sensitizing
hsa-miR-758	MI0003757	UUUGUGACCUGGUCCACUAACC	0.7816	0.0149	0.1768	0.0055	IR sensitizing

hsa-miR-760	MI0005567	CGGCUCUGGGUCUGUGGGGA	0.8509	0.0185	0.6354	0.0999	—
hsa-miR-765	MI0005116	UGGAGGAGAAGGAAGGUGAUG	0.0857	0.0068	0.0722	0.0142	Anti-proliferative
hsa-miR-766	MI0003836	ACUCCAGCCCCACAGCCUCAGC	0.5861	0.0154	0.1659	0.0319	IR sensitizing
hsa-miR-767-3p	MI0003763	UCUGCUCAUACCCCAUGGUUUCU	0.8636	0.0212	0.1810	0.0051	IR sensitizing
hsa-miR-767-5p	MI0003763	UGCACCAUGGUUGUCUGAGCAUG	0.8936	0.0262	0.2445	0.0181	IR sensitizing
hsa-miR-768-3p	MI0005117	UCACAAUGCUGACACUCAAACUGCUGAC	0.8040	0.0089	0.1908	0.0216	IR sensitizing
hsa-miR-768-5p	MI0005117	GUUGGAGGAUGAAAGUACGGAGUGAU	0.2207	0.0405	0.0926	0.0081	Anti-proliferative
hsa-miR-769-3p	MI0003834	CUGGGAUCUCCGGGGUCUUGGUU	0.8738	0.0325	0.3271	0.0594	IR sensitizing
hsa-miR-769-5p	MI0003834	UGAGACCUCUGGGUUCUGAGCU	0.6996	0.0082	0.2732	0.0441	IR sensitizing
hsa-miR-770-5p	MI0005118	UCCAGUACCACGUGUCAGGGCCA	0.7515	0.0363	0.1724	0.0130	IR sensitizing
hsa-miR-801	MI0005202	GAUUGCUCUGCGUGCGGAAUCGAC	0.7025	0.0048	0.1065	0.0138	IR sensitizing
hsa-miR-802	MI0003906	CAGUAACAAAGAUUCAUCCUUGU	0.7886	0.0276	0.3697	0.0506	IR sensitizing
hsa-miR-873	MI0005564	GCAGGAACUUGUGAGUCUCCU	0.6987	0.0062	0.2942	0.0211	IR sensitizing
hsa-miR-874	MI0005532	CUGCCCUGGCCCGAGGGACCGA	1.0246	0.0387	2.6905	0.0877	IR protective
hsa-miR-875-3p	MI0005541	CCUGGAAACACUGAGGUUGUG	0.8499	0.0092	1.5871	0.0540	—
hsa-miR-875-5p	MI0005541	UAUACCUCAGUUUUAUCAGGUG	0.7868	0.0206	0.6278	0.0628	—
hsa-miR-876-3p	MI0005542	UGGUGGUUUACAAAGUAAUUCA	0.2734	0.0087	0.0578	0.0043	Anti-proliferative
hsa-miR-876-5p	MI0005542	UGGAUUUCUUUGUGAAUCACCA	0.7955	0.0084	0.6314	0.0123	—
hsa-miR-877	MI0005561	GUAGAGGAGAUGGCGCAGGG	0.9912	0.0158	1.9139	0.0225	—
hsa-miR-877*	MI0005561	UCCUCUUCUCCCUCCUCCCAG	0.8140	0.0146	0.4357	0.0581	IR sensitizing
hsa-miR-885-3p	MI0005560	AGGCAGCGGGGUGUAGUGGAUA	0.2602	0.0445	0.0882	0.0098	Anti-proliferative
hsa-miR-885-5p	MI0005560	UCCAUUACACUACCCUGCCUCU	0.6969	0.0265	0.1925	0.0095	IR sensitizing
hsa-miR-886-3p	MI0005527	CGCGGGUGCUUACUGACCCUU	0.4644	0.0676	0.0382	0.0058	Anti-proliferative
hsa-miR-886-5p	MI0005527	CGGGUCGGAGUUAGCUCAAGCGG	0.7484	0.0032	2.3542	0.2416	IR protective
hsa-miR-887	MI0005562	GUGAACGGGCGCCAUCCCGAGG	0.7336	0.0269	0.3010	0.0417	IR sensitizing
hsa-miR-888	MI0005537	UACUCAAAAAGCUGUCAGUCA	1.0749	0.0147	2.6597	0.0495	IR protective
hsa-miR-888*	MI0005537	GACUGACACCUCUUUGGGUGAA	0.9271	0.0135	0.1030	0.0091	IR sensitizing
hsa-miR-889	MI0005540	UUAAUAUCGGACAACCAUUGU	0.6494	0.0133	0.1319	0.0088	IR sensitizing
hsa-miR-890	MI0005533	UACUUGGAAAGGCAUCAGUUG	0.8707	0.0390	0.0533	0.0057	IR sensitizing
hsa-miR-891a	MI0005524	UGCAACGAACCUGAGCCACUGA	0.7373	0.0084	0.5143	0.1170	—
hsa-miR-891b	MI0005534	UGCAACUUACCUGAGUCAUUGA	0.2264	0.0268	0.0583	0.0051	Anti-proliferative
hsa-miR-892a	MI0005528	CACUGUGUCCUUUCUGCGUAG	0.6746	0.0066	0.1790	0.0126	IR sensitizing
hsa-miR-892b	MI0005538	CACUGGCUCCUUUCUGGGUAGA	0.8056	0.0304	0.1421	0.0140	IR sensitizing
hsa-miR-9	MI0000467	UCUUUGGUUAUCUAGCUGUAUGA	0.5891	0.0321	0.0715	0.0059	IR sensitizing
hsa-miR-9	MI0000466	UCUUUGGUUAUCUAGCUGUAUGA	0.6023	0.0130	0.2033	0.0356	IR sensitizing
hsa-miR-9	MI0000468	UCUUUGGUUAUCUAGCUGUAUGA	0.6973	0.0338	0.2387	0.0371	IR sensitizing
hsa-miR-9*	MI0000466	AUAAAGCUAGAUAACCGAAAGU	0.6705	0.0431	0.0735	0.0094	IR sensitizing
hsa-miR-9*	MI0000467	AUAAAGCUAGAUAACCGAAAGU	0.4757	0.0058	0.0357	0.0084	Anti-proliferative
hsa-miR-9*	MI0000468	AUAAAGCUAGAUAACCGAAAGU	0.4899	0.0497	0.0455	0.0017	Anti-proliferative
hsa-miR-92	MI0000093	UAUUGCACUUGUCCCGGCCUGU	0.7725	0.0135	1.0849	0.1993	—
hsa-miR-92	MI0000094	UAUUGCACUUGUCCCGGCCUGU	0.6688	0.0046	2.0861	0.2660	IR protective
hsa-miR-920	MI0005712	GGGGAGCUGUGGAAGCAGUA	0.8542	0.0030	0.9291	0.0372	—
hsa-miR-921	MI0005713	CUAGUGAGGGACAGAACCAGGAUUC	0.7283	0.0074	0.2628	0.0113	IR sensitizing
hsa-miR-922	MI0005714	GCAGCAGAGAAUAGGACUACGUC	0.6947	0.0064	0.3676	0.0405	IR sensitizing
hsa-miR-923	MI0005715	GUCAGCGGAGGAAAAGAAACU	0.8434	0.0046	1.3210	0.0718	—
hsa-miR-924	MI0005716	AGAGUCUUGUGAUGUCUUGC	0.6991	0.0101	0.2422	0.0118	IR sensitizing
hsa-miR-92a-1*	MI0000093	AGGUUGGGAUCGGUUGCAAUGCU	0.2934	0.0522	0.0998	0.0209	Anti-proliferative
hsa-miR-92a-2*	MI0000094	GGGUGGGGAUUUGUUGCAUUAC	0.8557	0.0415	0.2502	0.0235	IR sensitizing
hsa-miR-92b	MI0003560	UAUUGCACUCGUCCCGGCCUCC	0.6330	0.0051	1.6427	0.2615	—
hsa-miR-92b*	MI0003560	AGGGACGGGACGCGGUGCAGUG	0.5920	0.0166	0.5061	0.0583	—
hsa-miR-93	MI0000095	CAAAGUGCUGUUCGUGCAGGUAG	0.8551	0.0077	1.0794	0.0701	—
hsa-miR-93*	MI0000095	ACUGCUGAGCUAGCACUUCCCG	0.5672	0.0067	0.0845	0.0062	IR sensitizing
hsa-miR-933	MI0005755	UGUGCGCAGGGAGACCUCUCCC	0.7827	0.0042	0.5547	0.0200	—
hsa-miR-934	MI0005756	UGUCUACUACUGGAGACACUGG	0.3986	0.0491	0.1013	0.0094	Anti-proliferative
hsa-miR-935	MI0005757	CCAGUUACCGCUUCCGCUACCGC	0.7700	0.0054	0.8101	0.1698	—
hsa-miR-936	MI0005758	ACAGUAGAGGGAGGAAUCGCAG	0.7824	0.0160	0.4115	0.0577	IR sensitizing
hsa-miR-937	MI0005759	AUCCGCGCUCUGACUCUCUGCC	0.6994	0.0103	0.5768	0.0114	—
hsa-miR-938	MI0005760	UGCCCUUAAAGGUGAACCCAGU	0.7252	0.0085	0.8297	0.0190	—
hsa-miR-939	MI0005761	UGGGGAGCUGAGGCUCUGGGGGUG	0.8513	0.0178	0.9736	0.1125	—
hsa-miR-940	MI0005762	AAGGCAGGGCCCCCGCUCCCC	0.1697	0.0039	0.0642	0.0036	Anti-proliferative
hsa-miR-941	MI0005766	CACCCGGCUGUGUGCACAUGUGC	0.7536	0.0063	0.8579	0.0633	—
hsa-miR-941	MI0005764	CACCCGGCUGUGUGCACAUGUGC	0.7746	0.0053	0.8826	0.0352	—
hsa-miR-941	MI0005765	CACCCGGCUGUGUGCACAUGUGC	0.7438	0.0036	0.9375	0.0317	—
hsa-miR-941	MI0005763	CACCCGGCUGUGUGCACAUGUGC	0.7499	0.0133	1.0242	0.0256	—
hsa-miR-942	MI0005767	UCUUCUCUGUUUUGGCCAUGUG	0.8396	0.0154	0.3676	0.0681	IR sensitizing
hsa-miR-943	MI0005768	CUGACUGUUGCCGUCCUCCAG	0.5568	0.0045	0.1424	0.0055	IR sensitizing
hsa-miR-944	MI0005769	AAAUUAUUGUACAUCGGAUGAG	0.5740	0.0382	0.0681	0.0028	IR sensitizing
hsa-miR-95	MI0000097	UUCAACGGGUAUUUAUUGAGCA	0.9062	0.0245	1.4136	0.1202	—

hsa-miR-96	MI0000098	UUUGGCACUAGCACAUUUUUGCU	0.7501	0.0178	0.4100	0.0196	IR sensitizing
hsa-miR-96*	MI0000098	AAUCAUGUGCAGUGCCAAUAUG	0.6279	0.0026	0.3646	0.0352	IR sensitizing
hsa-miR-98	MI0000100	UGAGGUAGUAAGUUGUAUUGUU	0.7694	0.0314	0.4744	0.0755	IR sensitizing
hsa-miR-99a	MI0000101	AACCCGUAGAUCCGAUCUUGUG	0.7978	0.0412	0.2253	0.0222	IR sensitizing
hsa-miR-99a*	MI0000101	CAAGCUCGCUUCUAUGGGUCUG	0.8450	0.0018	0.5779	0.1044	—
hsa-miR-99b	MI0000746	CACCCGUAGAACCGACCUUGCG	0.6616	0.0779	0.1692	0.0056	IR sensitizing
hsa-miR-99b*	MI0000746	CAAGCUCGUGUCUGUGGGUCCG	0.8627	0.0318	0.7699	0.2160	—

Supplementary Table 4. Radiation sensitizing miRNAs predicted to target radiation sensitizing DDR pathway genes using *in silico* analysis, microRNA.org.

	DNAPK	MAD2L2	BRCA2	NBN	RAD23B	RAD54L
miR-890		-0.5108		-0.1413	-0.0121	
miR-744-3p			-0.1147	-0.0084	-0.7604	
miR-32-3p			-0.1559	-0.6390	-0.0488	
miR-130b-5p	-0.0444				-0.0029	-0.4047

The highlighted cells corresponding to the miRNAs with a mirSVR score < -0.5.

Supplementary Table 5. Potential DDR pathway genes predicted to be targeted by miR-890 or miR-744-3p using *in silico* analysis, microRNA.org, and corresponding mirSVR scores.

DDR pathway genes	miRNA	mirSVR score
WEE1	miR-890	-1.1805
XPC	miR-890	-0.9251
MAD2L2	miR-890	-0.5108
KU80	miR-890	-0.4843
XLF	miR-744-3p	-0.8632
RAD23B	miR-744-3p	-0.7604
MCL1	miR-744-3p	-0.6699
